# Global genome mining-driven discovery of an unusual biosynthetic logic for fungal polyketide–terpenoid hybrids†

**DOI:** 10.1039/d3sc06001b

**Published:** 2024-01-17

**Authors:** Dexiu Yan, Yudai Matsuda

**Affiliations:** a Department of Chemistry, City University of Hong Kong Tat Chee Avenue Kowloon Hong Kong SAR China ymatsuda@cityu.edu.hk

## Abstract

Genome mining has facilitated the efficient discovery of untapped natural products. We performed global genome mining in fungi and discovered a series of biosynthetic gene clusters (BGCs) that appeared to afford polyketide–terpenoid hybrids *via* a distinct biosynthetic mechanism from those adopted by known pathways. Characterization of one of the BGCs revealed that it yields the drimane–phthalide hybrid 1. During the biosynthesis of 1, the farnesyl group is unusually introduced by the dimethylallyltryptophan synthase-type prenyltransferase MfmD and is then cyclized by the Pyr4-family terpene cyclase MfmH. The replacement of MfmH with its homologue OcdTC gave another hybrid molecule with a monocyclic terpenoid moiety. Moreover, PsetPT, an MfmD homologue, was found to perform dimethylallylation and was then engineered to install a geranyl group. Our study unraveled an unusual biosynthetic mechanism for fungal phthalide–terpenoid hybrids and provided insights into how their structural diversification could be achieved.

## Abstract 2

Global genome mining resulted in the discovery of an unusual biosynthetic mechanism for fungal meroterpenoids, in which the dimethylallyltryptophan synthase-type prenyltransferase MfmD installs a farnesyl group for subsequent cyclization.

## Graphical abstract

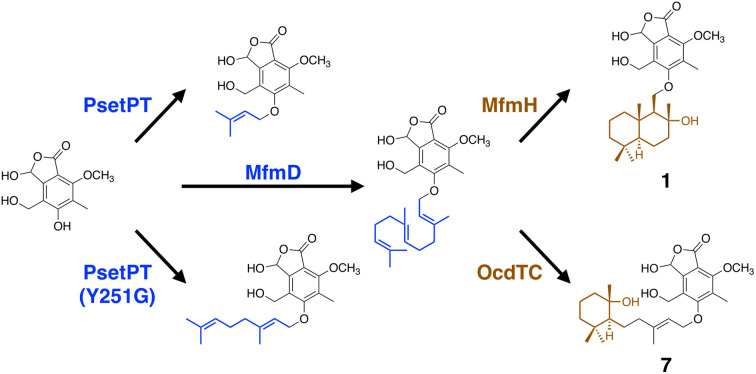

## Introduction

Genome mining-guided natural product discovery, which seeks to access unexploited natural products by utilizing genome sequence data, has proven to be useful and effective for more than a decade.^1–3^ Along with the rapid accumulation of genome sequences of a diverse range of organisms, an increasing number of natural products have been identified using genome mining approaches. Given that traditional methodologies often lead to the rediscovery of known natural products, genomic-based strategies can now be considered a promising solution for the continuous discovery of untapped natural products.^4,5^ Nevertheless, given the availability of a large number of genome sequences, it is critical to prioritize and selectively extract biosynthetic gene clusters (BGCs) for natural products to identify potentially novel metabolites.

To overcome such a challenge in genome mining, we recently developed a fungal genome mining tool that can readily extract BGCs satisfying user-defined criteria.^6^ We used our genome mining tool to mine approximately 2000 fungal genomes to extract BGCs that encode a homologue of Pyr4, which is the noncanonical terpene cyclase involved in the biosynthesis of the fungal meroterpenoid pyripyropene A.^7^ Although Pyr4 homologues have been found in diverse natural product pathways,^8^ the global genome mining process yielded several BGCs with unprecedented features.^6^ Further experimental characterization of selected BGCs resulted in the discovery of fungal onoceroid terpenoids, demonstrating the usefulness of our genome mining tool in discovering novel natural products. Interestingly, in addition to the onoceroid BGCs, many more BGCs that are somewhat different from known BGCs were extracted. Thus, further characterization of these untapped BGCs may result in the identification of previously unreported metabolites.

In this study, through reexamination of previously extracted BGCs, we discovered a series of BGCs for fungal polyketide–terpenoid hybrids that are distinct from previously characterized BGCs. Experimental characterization of one selected BGC designated as the *mfm* cluster identified a new meroterpenoid molecule synthesized by an unusual biosynthetic mechanism. Furthermore, we focused on two additional BGCs similar to the *mfm* cluster and achieved structural diversification by incorporating genes from these BGCs into the *mfm* pathway.

## Results and discussion

### Discovery of fungal meroterpenoid BGCs with unusual features

**Fig. 1:**
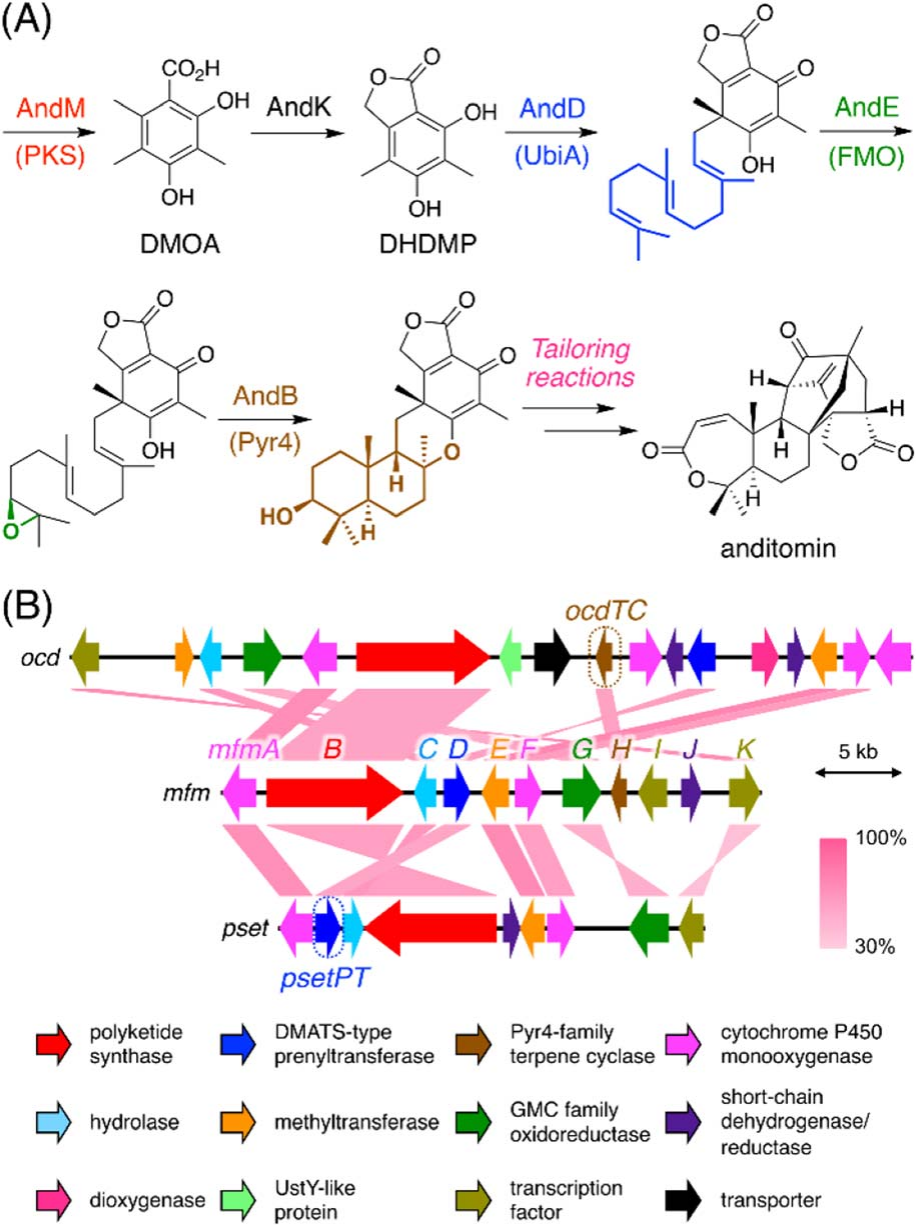
(A) Biosynthesis of anditomin^11^ as a typical biosynthetic model of fungal polyketide–terpenoid hybrids synthesized using a Pyr4-family terpene cyclase. PKS: polyketide synthase; UbiA: UbiA-like prenyltransferase; FMO: FAD-dependent monooxygenase; Pyr4: Pyr4-family terpene cyclase. (B) Schematic representations of the *mfm*, *ocd*, and *pset* clusters from *Annulohypoxylon moriforme* CBS 123579, *Colletotrichum orchidophilum* IMI 309357, and *Aspergillus pseudotamarii* CBS 117625, respectively, and BLASTp comparisons of each gene product.

Our previous genome mining study targeting BGCs encoding a Pyr4-family terpene cyclase resulted in the extraction of 182 BGCs encoding at least one polyketide synthase (PKS), which are apparently responsible for the biosynthesis of a polyketide–terpenoid hybrid.^6^ The biosynthesis of such polyketide–terpenoid hybrids can be generalized as follows (Fig. 1A).^9,10^ First, a polyketide molecule is formed by a PKS, which is followed by prenylation catalyzed by a membrane-embedded UbiA-like prenyltransferase. An FAD-dependent monooxygenase then epoxidizes one of the olefinic double bonds in the prenyl chain, and a Pyr4-family terpene cyclase subsequently protonates the epoxide to conduct a cyclization reaction. Additional reactions, such as phthalide formation in the anditomin pathway,^11^ may also occur during backbone synthesis. Further tailoring reactions yield the end pathway product. Intriguingly, we found that 24 (out of 182) BGCs encoded a PKS and a dimethylallyltryptophan synthase (DMATS)-type prenyltransferase but lacked a UbiA-like (or other types of) prenyltransferase gene. This observation indicated that the Pyr4-family terpene cyclases from these BGCs cyclize a prenyl moiety installed by the DMATS-type prenyltransferase; however, such a biosynthetic mechanism has never been reported. In addition, these clusters lacked an epoxidase gene, and therefore, cyclization by these Pyr4 homologues could occur without epoxidation, which is, however, highly unusual with only a few exceptions, such as the reaction catalyzed by MacJ during macrophorin biosynthesis.^12^ Notably, the Pyr4 homologues identified herein form a new clade in the phylogenetic analysis with characterized enzymes (Fig. S1†). Moreover, great diversity was observed within these BGCs (Fig. S1†), altogether suggesting that the BGCs are involved in the biosynthesis of a diverse range of previously uncharacterized meroterpenoids. It should be mentioned that these BGCs could have been discovered using antiSMASH^13^ or by standard BLAST search; however, antiSMASH does not recognize *pyr4* homologues as biosynthetic genes and classifies these BGCs as “T1PKS, indole” (type I PKS + indole), which might be misleading (Fig. S2†). The advantageous point of our methodology is that BGCs with a *pyr4* homologue (or another user-selected gene) can be automatically extracted in a single step.

### Characterization of the *mfm* cluster from *Annulohypoxylon moriforme*

To experimentally investigate whether the BGCs that we identified produce new meroterpenoid species, we focused on the BGC from *Annulohypoxylon moriforme* CBS 123579,^14^ designated as the *mfm* cluster, for further characterization (Fig. 1B and Table S2†). The *mfm* cluster encodes the non-reducing (NR)-PKS MfmB, the two cytochrome P450 monooxygenases MfmA and MfmF, the metallo-hydrolase MfmC, the DMATS-type prenyltransferase MfmD, the methyltransferase MfmE, the glucose–methanol–choline family oxidoreductase MfmG, the Pyr4-family terpene cyclase MfmH, and the short-chain dehydrogenase/reductase MfmJ, as well as the two transcription factors MfmI and MfmK. It should be noted that MfmA and MfmC are homologous to the N-terminal P450 domain and the C-terminal hydrolase domain of AndK,^11^ respectively, which transforms the aromatic polyketide 3,5-dimethylorsellinic acid (DMOA) into the phthalide 5,7-dihydroxy-4,6-dimethylphthalide (Fig. 1A).

**Fig. 2:**
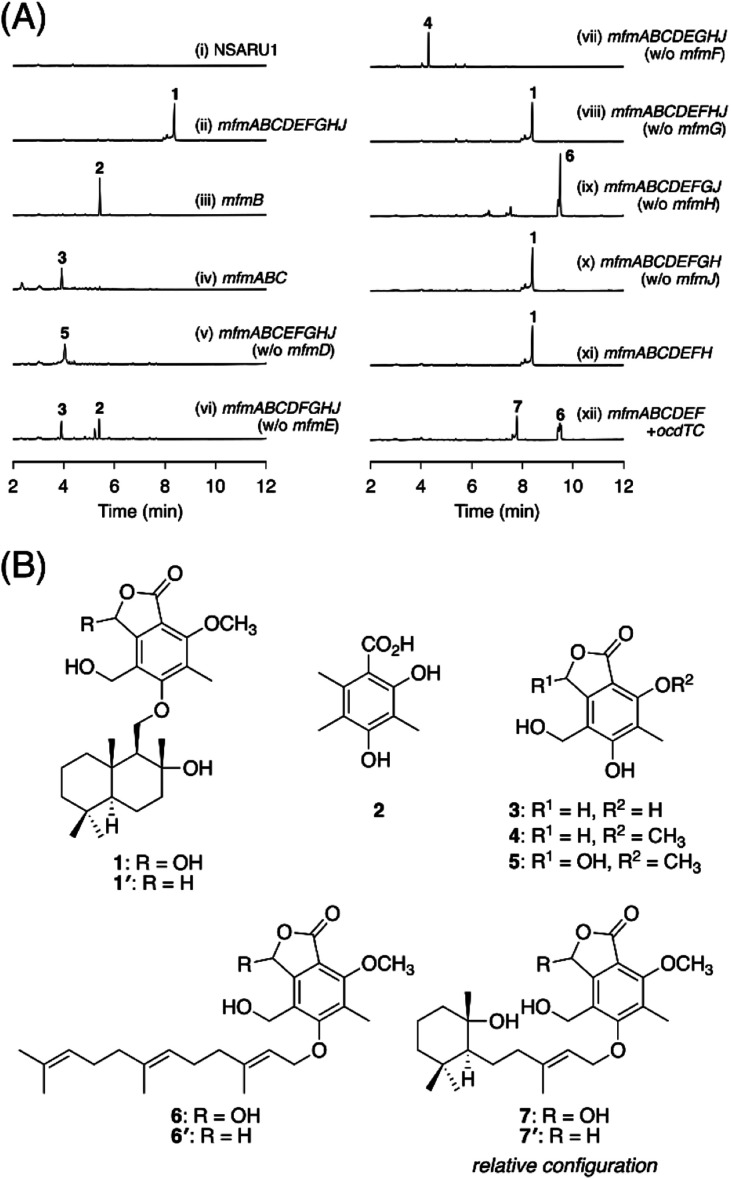
(A) HPLC profiles of the metabolites from *A. oryzae* transformants. The chromatograms were monitored at 254 nm. (B) Structures of compounds 1–7′. Note that the absolute configuration of 7 could not be established in this study.

To identify the metabolite derived from the *mfm* cluster, we first introduced the nine enzyme-encoding genes (*mfmA*, -*B*, -*C*, -*D*, -*E*, -*F*, -*G*, -*H*, and -*J*) into *Aspergillus oryzae* NSARU1.^15^ HPLC analysis of the metabolites from the *A. oryzae* transformant revealed the presence of a major product 1, which was not observed in the host strain (Fig. 2A, traces i and ii). HR-MS analysis indicated the molecular formula of 1 as C_26_H_38_O_7_ (Fig. S3†), which is reasonable for a polyketide–sesquiterpenoid hybrid molecule. We then sought to isolate and characterize 1, but its unstable nature hampered its structural determination. Fortunately, we found that 1 can be transformed into a stable compound 1′ with the molecular formula C_26_H_38_O_6_ by treatment with NaBH_4_, indicating that one oxygen atom was removed upon reduction. NMR analysis of 1′ revealed that 1′ is a drimane–phthalide hybrid (Fig. 2B and S9 to S15†), and its absolute structure was confirmed by single-crystal X-ray diffraction analysis with a Flack parameter of −0.06(8) (Fig. S4;† CCDC 2293718). Thus, we reasoned that 1 is the hydroxylated form of 1′, with hydroxylation at the C-3′ position (Fig. 2B), as a similar hydroxyfuranone has been reported to be reduced to furanone by NaBH_4_.^16^ Compound 1 is the 11′-*O*-desmethyl analogue of fendlerols A and B.^17^ This finding also confirmed that the *mfm* cluster is responsible for the biosynthesis of a polyketide–terpenoid hybrid *via* a different mechanism from those in characterized pathways.

Next, we sought to elucidate the biosynthetic pathway leading to 1 using the *A. oryzae* heterologous expression system. First, the PKS gene *mfmB* was solely expressed in *A. oryzae*, which resulted in the formation of DMOA^11^ (2) (Fig. 2A trace iii, S3 and S5†). The next biosynthetic steps were expected to involve the P450 MfmA and the hydrolase MfmC, which would convert 1 into a phthalide form. Thus, *mfmA* and *mfmC* were coexpressed with *mfmB*, and the resultant transformant yielded a new product, 3 (Fig. 2A, trace iv), which was characterized as 5,7-dihydroxy-4-(hydroxymethyl)-6-methylphthalide (Fig. 2B and S16 to S20†). The subsequent biosynthetic pathway was unpredictable at this point, and therefore, we constructed a series of eight gene-expressing transformants that lacked one of the biosynthetic genes. The transformant lacking the methyltransferase gene *mfmE* yielded 2 and 3, but no additional metabolite (Fig. 2A, trace vi), indicating that MfmE accepts 3 as its substrate. When the P450 gene *mfmF* was omitted, the methylated analogue of 3, 5-hydroxy-4-(hydroxymethyl)-7-methoxy-6-methylphthalide (4),^18^ was obtained (Fig. 2A trace vii, 2B, S21, and S23†). The oxidized product of 4, 5, was detected in the transformant missing the DMATS-type prenyltransferase gene *mfmD* (Fig. 2A, trace v). We found that 5 was unstable, just like 1, but could be reduced to 4 by NaBH_4_ (Fig. S22†). This observation indicated that 5 is cyclopolic acid,^19^ which is the C-3 hydroxy form of 4 (Fig. 2B). Finally, the transformant without the terpene cyclase gene *mfmH* afforded 6 (Fig. 2A, trace ix), the reduction of which gave the farnesylated form of 4, 6′ (Fig. 2B and S24 to S29†). Thus, it was deduced that 6 is 5-*O*-farnesylcyclopolic acid. Meanwhile, the removal of *mfmG* or *mfmJ* resulted in a similar metabolic profile to that of the transformant with all nine genes (Fig. 2A, traces viii and x), indicating that these two genes are not involved in the biosynthesis of 1. Indeed, the seven-gene-expression system without *mfmG* and *mfmJ* afforded 1 (Fig. 2A, trace xi).

**Scheme 1:**
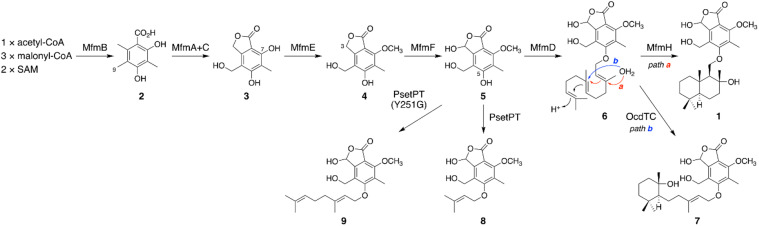
Proposed biosynthetic pathway of 1 and related metabolites.

On the basis of the experimental results described above, the biosynthetic route to 1 is proposed as follows (Scheme 1). First, the NR-PKS MfmB forms DMOA (2), which is then transformed into the phthalide 3 by the P450 MfmA and the hydrolase MfmC. MfmA and MfmC should work in an analogous manner to AndK,^11^ but MfmA performs an additional hydroxylation at C-9. Subsequently, the methyltransferase MfmE catalyzes 7-*O*-methylation to yield 4, which undergoes C-3 hydroxylation by the P450 MfmF. The resultant cyclopolic acid (5) is then farnesylated by the DMATS-type prenyltransferase MfmD to afford 6. Finally, the Pyr4-family terpene cyclase MfmH cyclizes the farnesyl moiety of 6 into a drimane-like structure, thus completing the biosynthesis of 1. Interestingly, 1 appears to be the precursor of some previously described meroterpenoids,^17,20^ such as fendlerinines, fendlerins, and fendlerols, although we did not identify the downstream metabolites of 1 in this study.

Biosynthetically, 1 is classified as a member of the DMOA-derived meroterpenoids, which include a large number of molecules with intriguing molecular architectures.^9,10^ The biosynthesis of DMOA-derived meroterpenoids has been intensively studied in the last decade;^11,15,21–29^ however, the biosynthesis of 1 is distinct from the biosynthesis of other DMOA-derived meroterpenoids. In previously described pathways, the farnesyl group is introduced only by UbiA-like prenyltransferases, which catalyze dearomatizing *C*-prenylation (Fig. 1A). In contrast, the DMATS-type prenyltransferase MfmD is responsible for farnesylation in the biosynthesis of 1 and catalyzes *O*-prenylation, instead of *C*-prenylation. Although DMATS-type prenyltransferases are widespread in natural product biosynthesis pathways, the majority of them use dimethylallyl pyrophosphate as their substrate, and farnesylation is rare among reactions catalyzed by DMATS-type prenyltransferases.^30,31^ Intriguingly, this is the first example in which a Pyr4-family terpene cyclase cyclizes the prenyl unit introduced by a DMATS-type prenyltransferase. In addition, it is noteworthy that, unlike other DMOA-derived meroterpenoids, the biosynthesis of 1 does not require epoxidation of the prenyl group prior to the cyclization event. Collectively, the biosynthetic pathway of 1 provides a new mechanism for DMOA-derived meroterpenoid biosynthesis. Meanwhile, 1 was not detected in the metabolites of *A. moriforme* CBS 123579 cultivated under several different conditions, suggesting that the *mfm* cluster is a cryptic BGC in this fungus.

### Diversity generation by terpene cyclases

It is well known that terpene cyclases play a critical role in the diversity generation of fungal meroterpenoids and that a common precyclized intermediate can be cyclized into different products.^8^ Thus, we next sought to obtain another molecule synthesized *via* a different cyclization mode from that of MfmH. To this end, we focused on the MfmH homologue from *Colletotrichum orchidophilum* IMI 309357,^32^ which displayed 56% protein sequence identity with MfmH and was tentatively designated as OcdTC (GenBank: XP_022481694.1) (Fig. 1B). To obtain the product of the terpene cyclase, *ocdTC* was coexpressed with the biosynthetic genes for 6 in *A. oryzae*. Consequently, the transformant with *ocdTC* yielded a new metabolite, 7 (Fig. 2A, trace xii), which was found to be an isomer of 1. NMR analysis of 7′, the reduced form of 7, revealed that 7′ possesses a monocyclic terpenoid moiety (Fig. 2B and S30 to S36†), thus confirming that OcdTC has a distinct cyclization activity from MfmH (Scheme 1). Interestingly, the *ocd* cluster encodes several tailoring enzymes, the homologues of which are not encoded by the *mfm* cluster. As 7 is apparently not a precursor of a known fungal natural product, further characterization of the *ocd* cluster may lead to the discovery of new meroterpenoid species.

### Identification and characterization of a prenyltransferase that incorporates a shorter prenyl chain

**Fig. 3:**
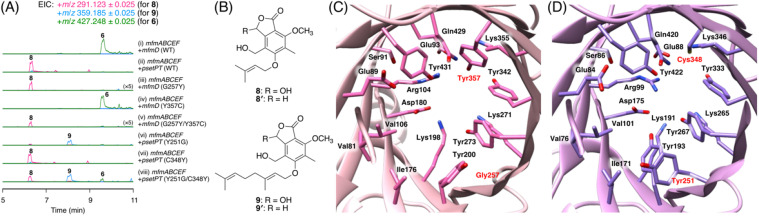
(A) LS-MS profiles of the metabolites from *A. oryzae* transformants. (B) Structures of compounds 8–9′. (C and D) Predicted substrate-binding sites of (C) MfmD and (D) PsetPT. Amino acid residues that are different from their corresponding residues are shown in red.

To further investigate the prevalence of BGCs homologous to the *mfm* cluster, we next performed a cblaster^33^ search using Mfm proteins as queries. Interestingly, we found that some fungi harbor BGCs that are somewhat similar to the *mfm* cluster but lack a terpene cyclase gene (Fig. S6†). One such example is the BGC from *Aspergillus pseudotamarii* CBS 117625,^34^ which we named the *pset* cluster (Fig. 1B). The *pset* cluster encodes homologues of MfmA–F, which are collectively required for the formation of the precyclized intermediate in the biosynthesis of 1. The DMATS-type prenyltransferase encoded by the *pset* cluster, tentatively designated as PsetPT (GenBank: XP_031913546.1), displays 54% amino acid sequence identity with MfmD, but given the lack of a terpene cyclase gene, we expected that PsetPT might introduce a shorter prenyl chain onto 5. To examine the function of PsetPT, *psetPT* was expressed along with *mfmA*, *-B*, -*C*, -*E*, and -*F*, in *A. oryzae*, and the transformant synthesized a new product, 8, which was not obtained from MfmD (Fig. 3A, traces i and ii). The reduced form of 8, 8′, was then characterized to be the dimethylallylated form of 4, and therefore, it was deduced that 8 is 5-*O*-dimethylallylcyclopolic acid (Fig. 3B and S37 to S42†), demonstrating that PsetPT indeed possesses a different prenyl donor specificity from MfmD (Scheme 1). It should be noted that 8 seems to be a precursor of the chromanol derivatives obtained from the fungus *Aspergillus duricaulis*.^35,36^

We next sought to clarify the factor(s) that affects the substrate specificity of the two prenyltransferases. To this end, we obtained the AlphaFold2 (ref. ^37^)-generated models of MfmD and PsetPT using ColabFold.^38^ The substrate-binding sites of MfmD and PsetPT were then predicted based on the crystal structure of the DMATS FgaPT2 in the complex with dimethylallyl *S*-thiolodiphosphate (DMSPP)^39^ (Fig. S7†). A comparison of the predicted substrate-binding sites of MfmD and PsetPT revealed that Gly257 and Tyr357 in MfmD are substituted by Tyr251 and Cys348 in PsetPT, respectively (Fig. 3C, D, and S8†). To examine the importance of these residues in determining substrate specificity, we introduced a series of variants of the two enzymes into the *A. oryzae* strain synthesizing 5. Although the Y357C mutation in MfmD did not cause an obvious change in enzyme activity, both the G257Y and G257Y/Y357C variants of MfmD lost their ability to farnesylate 5 but yielded the dimethylallylated product 8 (Fig. 3A, traces iii to v). Interestingly, the Y251G variant of PsetPT afforded a new metabolite, 9 (Fig. 3A, trace iv), of which the reduced product 9′ was determined to be the geranylated form of 4 (Fig. 3B and S43 to S48†). Furthermore, the Y251G/C348Y variant yielded three differently prenylated products, 6, 8, and 9, whereas the C348Y single mutation apparently had no effect on substrate selectivity (Fig. 3A, traces vii and viii). Collectively, these findings indicate that a single mutation can alter the prenyl donor specificity of the two enzymes, and Gly257 in MfmD and Tyr251 in PsetPT play key roles in substrate selection (Scheme 1).

Attempts have been made to engineer the functions of DMATS-type (or ABBA-type) prenyltransferases.^30,31^ The prenyl donor selectivity of some of these enzymes has been successfully altered by site-directed mutagenesis. For example, Met328 of FgaPT2, which is a C4-DMATS from *Aspergillus fumigatus*, has been found to be critical for its prenyl donor specificity, and several variants of Met328 exhibit a higher preference toward geranyl pyrophosphate and farnesyl pyrophosphate.^40^ Similar mutagenesis has been performed on TleC and MpnD, both of which use the same prenyl acceptor molecule but display different prenyl donor specificities, identifying key amino acid residues for their substrate preferences.^41^ Interestingly, Gly257 in MfmD (and Tyr251 in PsetPT) does not correspond to the residues previously identified to be important for prenyl donor specificity, indicating that different enzymes adopt residues at different positions to control their product selectivity.

## Conclusions

In this study, we discovered fungal meroterpenoid BGCs through a global genome mining approach. We initially focused on the *mfm* cluster and found that this BGC is responsible for the biosynthesis of the drimane–phthalide hybrid 1. Although the molecular scaffold of 1 is not novel, its biosynthetic mechanism is distinct from the current consensus on the biosynthesis of fungal meroterpenoids synthesized by the involvement of a Pyr4-family cyclase. The pathway leading to 1 involves the DMATS-type prenyltransferase MfmD for farnesylation, which is typically performed by UbiA-type prenyltransferases. Our study indicated that BGCs homologous to the *mfm* cluster are widespread in fungi and are involved in the biosynthesis of a diverse range of molecules. Indeed, we showed that the biosynthesis of 1 can be branched by utilizing a homologous terpene cyclase or prenyltransferase from different fungi, providing insights into how diversity generation is achieved in the biogenesis of this class of natural products. Furthermore, we successfully engineered the DMATS-type prenyltransferase PsetPT into a geranyltransferase. This work provides a foundation for further genome mining and engineered biosynthesis of fungal phthalide–terpenoid derivatives. In addition, our study demonstrated the utility of the *A. oryzae* expression system for the characterization of fungal orphan BGCs. Notably, biosynthetic genes from two sordariomycetes fungi (*i.e.*, *Annulohypoxylon* and *Colletotrichum*), which are somewhat distantly related to *A. oryzae* (eurotiomycetes),^42^ were successfully expressed, providing a sufficient amount of metabolites for structural characterization.

In conclusion, we demonstrated the usefulness and efficacy of global genome mining in identifying novel biosynthetic mechanisms, even when focusing on a well-characterized family of enzymes. Global genome mining is expected to further facilitate the discovery of unexploited natural products and biosynthetic reactions.

## Data availability

Crystallographic data for compound 1′ has been deposited at the CCDC under 2293718. The other datasets supporting this article have been uploaded as part of the ESI Material.†

## Author contributions

Y. M. designed the research and conducted the bioinformatic analysis. D. Y. performed the experiments. Both authors analyzed the data and co-wrote the manuscript.

## Conflicts of interest

There are no conflicts to declare.

## Supplementary Material

SC-015-D3SC06001B-s001

SC-015-D3SC06001B-s002
