# Supplementary material for: Global genome mining-driven discovery of an unusual biosynthetic logic for fungal polyketide–terpenoid hybrids
Source: Chem Sci. 2024 Jan 17;15(8):3011–7. doi: 10.1039/d3sc06001b (PMC10882540; doi:10.1039/d3sc06001b)
Supplement: SC-015-D3SC06001B-s001 [file SC-015-D3SC06001B-s001.pdf]

Electronic Supplementary Material (ESI) for Chemical Science.
This journal is © The Royal Society of Chemistry 2024

Supporting Information for

Global genome mining-driven discovery of an unusual biosynthetic logic for fungal polyketide–terpenoid hybrids

Dexiu Yan and Yudai Matsuda*

Department of Chemistry, City University of Hong Kong, Tat Chee Avenue, Kowloon, Hong Kong SAR, China

*Correspondence should be addressed to Yudai Matsuda (ymatsuda@cityu.edu.hk).

Table of Contents

Supplementary Materials and Methods **S2-S7**

Supplementary Tables S1-S6 **S8-S12**

Supplementary Figures S1-S48 **S13-S43**

Supplementary Data **S44-S47**

Supplementary References **S48**

Supplementary Materials and Methods

General experimental procedures

Organic solvents were purchased from Anaqua (Hong Kong) Co. Ltd., and other chemicals were purchased from Wako Chemicals Ltd., Thermo Fisher Scientific, and J&K Scientific Ltd. unless noted otherwise. Oligonucleotide primers (Table S4) were purchased from Beijing Genomics Institute. PCR was performed using a T100™ Thermal Cycler (Bio-Rad Laboratories, Inc.) with Phanta Max Super-Fidelity DNA Polymerase (Vazyme Biotech Co., Ltd). Preparative HPLC was performed on a Waters 1525 Binary HPLC pump with a 2998 photodiode array detector (Waters Corporation). Flash chromatography was performed using an Isolera Spektra One flash purification system (Biotage). NMR spectra were obtained at 600 MHz (^{1}H)/150 MHz (^{13}C) or 400 MHz (^{1}H)/100 MHz (^{13}C) with a Bruker Ascend Avance III HD spectrometer or Bruker Avance III spectrometer, and chemical shifts were recorded with reference to solvent signals (^{1}H NMR: $CDCl_3$ 7.26 ppm, CD_3OD 3.31 ppm, acetone-d_6 2.05 ppm, DMSO-d_6 2.50 ppm; ^{13}C NMR: $CDCl_3$ 77.0 ppm, CD_3OD 49.0 ppm, acetone-d_6 29.8 ppm, DMSO-d_6 39.5 ppm). HR-ESI-MS spectra were obtained with SCIEX X500R Q-TOF mass spectrometer. Samples for LC-MS analysis were injected into a SCIEX ExionLC AD System with a SCIEX X500R Q-TOF mass spectrometer, using a Luna® Omega C18 column (1.6 µm, 100 Å, 2.1 x 100 mm; Phenomenex). Optical rotations were measured with a P-2000 Digital Polarimeter (JASCO Corporation).

Strains

Annulohypoxylon moriforme CBS 123579 and *Aspergillus pseudotamarii* CBS 117625 were purchased from the Westerdijk Fungal Biodiversity Institute, whereas *Colletotrichum orchidophilum* IMI 309357 was obtained from the Centre for Agriculture and Bioscience International. *Aspergillus oryzae* NSARU1 (*niaD$^-$, sC$^-$, ΔargB, adeA$^-$, pyrG$^-$*)[1] was utilized as the fungal heterologous expression host. Standard DNA engineering was performed with *Escherichia coli* DH5α (Takara Bio Inc).

Phylogenetic analysis of Pyr4 homologues

The sequences of known Pyr4-family terpene cyclases and their homologues identified in this study were first aligned using MUSCLE[2] (version 5.1), and the conserved sequences were extracted with Gblocks[3] (version 0.91b). A maximum likelihood (ML) phylogenetic tree was generated with FastTree[4] (version 2.1.11), and the resultant phylogeny was used as a starting tree to infer an ML tree using RaxML[5] (version 8.0.0) under the LG+G+F model, as identified by ProtTest[6] (version 3.4.2). The Pyr4 homologues of bacterial origin were used as outgroups. The phylogenetic tree was visualized with Geneious Prime 2023.2.1 (https://www.geneious.com).

Construction of fungal transformation plasmids

To construct fungal expression plasmids for *A. oryzae*, each gene in the *mfm* cluster, *ocdTC*, or *psetPT* was first amplified from the genomic DNA (gDNA) of *A. moriforme* CBS 123579, *C. orchidophilum* IMI 309357, or *A. pseudotamarii* CBS 117625, respectively, with the primers described in Table S4 and Table S5. Each amplified DNA fragment was then introduced into the pTAex3 vector[7] except for *mfmA*, whereas *mfmA* was ligated into the pPyrG vector,[1] using a ClonExpress Ultra One Step Cloning Kit (Vazyme Biotech Co., Ltd). Subsequently, DNA fragments harboring the *amyB* promoter (*PamyB*) and the *amyB* terminator (*TamyB*) were amplified from the pTAex3-based plasmids, and further introduced into the already constructed single gene-containing vector or another vector, pAdeA,[8] and pAdeA-HR,[9] The expression plasmids for the *mfmD* and *psetPT* variants were created by PCR method with the mutation primers, using an In-Fusion® Snap Assembly Master Mix (Takara Bio Inc). Detailed methods for the construction of the plasmids used in this study are summarized in Table S5.

Fungal transformation

For the heterologous reconstitution of the *mfm* pathway, *Aspergillus oryzae* NSARU1 was first transformed with pTAex3-mfmB by the previously reported protoplast–polyethylene glycol method.[10] The resultant transformant was further transformed twice using the two plasmids, pPyrG-mfmA+C+D+E and pAdeA-mfmF+G+H+J, yielding the nine gene-expressing *A. oryzae* strain. When constructing the strains lacking one or more genes, plasmids with a fewer number of genes were used.

For the characterization of *ocdTC*, the *A. oryzae* strain with *mfmB* was transformed twice using the two plasmids, pPyrG-mfmA+C+E+F and pAdeA-mfmD+ocdTC.

To analyze the functions of PsetPT, as well as the MfmD and PsetPT variants, the *A. oryzae* strain with *mfmB* was initially transformed using pPyrG-mfmA+C+E+F, and the resultant five gene-expressing strain was transformed using a pAdeA-HR-based plasmid harboring either a wild-type or variant prenyltransferase gene by the protoplast–polyethylene glycol method coupled with CRISPR-Cas9-guided homologous recombination.[1, 9, 11]

The transformants created in this study and the plasmids used for the transformation are given in Table S6.

LC-MS analysis of metabolites derived from *A. oryzae* transformants

To analyze the metabolites produced by each *A. oryzae* transformant, the transformants were cultivated on a DPY agar plate [2% dextrin, 1% hipolypepton (Nihon Pharmaceutical Co., Ltd.), 0.5% yeast extract, 0.5% KH_2PO_4, 0.05% $MgSO_4$•$7H_2O$, and 1.5% agar] for around one week at 30 °C. A small piece of fungal mycelia and agar was cut from the plate, soaked in ethyl acetate, and extracted using an ultrasonic bath. The ethyl acetate layer was transferred to a new tube, and the solvent was removed using nitrogen gas flow. The residue was dissolved in methanol or acetonitrile and analyzed by LC-MS, with a solvent system of 20 mM formic acid (solvent A) and acetonitrile containing 20 mM formic acid (solvent B), at a flow rate of 0.4 mL/min and a

column temperature of 40 °C. HPLC separation was performed using a linear gradient from 10:90 (solvent B/solvent A) to 100:0 for 10 min, 100:0 for the following 3 min, and a linear gradient from 100:0 to 10:90 within the following 2.0 min, and then 10:90 for 2.5 min of equilibrium.

Isolation of each metabolite from *A. oryzae* transformants

To isolate each metabolite, *A. oryzae* transformants were cultivated on DPY agar plates (the volume of medium in one plate is *ca.* 20 mL) for around one week at 30 °C. The resulting fungal cultures, including agar medium, were crushed into small pieces, soaked in ethyl acetate, and extracted twice using an ultrasonic bath. After filtration, ethyl acetate was removed in vacuo. The resultant crude extract was fractionated by flash chromatography or open silica gel chromatography. The fractions containing the targeted compound were subjected to a reduction reaction and/or were further purified by preparative HPLC or open-column chromatography. For preparative HPLC, a COSMOSIL $5C_{18}$-AR-II column (10 i.d. x 250 mm, Nacalai Tesque, Inc) was used for the purification of **4** (which was obtained by the reduction of **5**), **8′**, and **9′**, whereas an XBridge BEH C18 OBD Prep Column (100 Å, 5 µm, 19 i.d. x 250 mm; Waters Corporation) for the purification of **3** and **4** (which was isolated from the *A. oryzae* transformant).

For the reduction reaction, the partially purified substrate was initially dissolved in methanol on ice. Subsequently, an excess amount of sodium borohydride ($NaBH_4$) was gradually added to the solution until the reaction was completed. The pH value of the reaction mixture was then adjusted to 7 using hydrochloric acid. The solvent was removed in vacuo, and the resulting residue was dissolved in water and extracted using ethyl acetate. The reduced product was finally purified by open-column chromatography or preparative HPLC. Purification methods for each compound are described in detail below.

Purification condition for compound **1′**:

The extract of *A. oryzae*/*mfmBACDEFGHJ* cultivated on 150 DPY agar plates (1.8 g) was subjected to open silica gel chromatography and eluted stepwise using hexane:acetone gradient (6:1 to 2:1). Fractions containing **1** (184.0 mg) were then concentrated and reduced using 0.4 g of $NaBH_4$. The reaction product was further purified by open silica gel chromatography (hexane: acetone 12:1) to yield 63.2 mg of **1′**.

Purification condition for 5,7-dihydroxy-4-(hydroxymethyl)-6-methylphthalide (**3**):

The extract of *A. oryzae*/*mfmBAC* cultivated on 150 DPY agar plates (0.89 g) was subjected to flash chromatography and eluted stepwise using dichloromethane:ethyl acetate gradient (100:0 to 0:100). Fractions that contained **3** (43.3 mg) were then purified by reverse-phase preparative HPLC (13% aqueous acetonitrile, 8.0 mL/min) to yield 11.2 mg of **3**.

Purification condition for 5-hydroxy-4-(hydroxymethyl)-7-methoxy-6-methylphthalide (**4**):

The extract of *A. oryzae*/*mfmBACE* cultivated on 60 DPY agar plates (0.86 g) was subjected to flash chromatography and eluted stepwise using dichloromethane:ethyl acetate gradient (100:0 to 0:100). Fractions that contained **4** (126.5 mg) were then purified by reverse-phase preparative HPLC (19% aqueous acetonitrile, 8.0 mL/min) to yield of 35.9 mg of **4**.

In addition, **4** was also obtained from the reduction of **5**. The extract of *A. oryzae*/*mfmBACEF* cultivated on 100 DPY agar plates (0.76 g) was subjected to open silica gel chromatography and eluted stepwise using hexane:acetone gradient (4:1 to 2:1). Fractions containing **5** (29.5 mg) were then concentrated and reduced using 0.1 g of $NaBH_4$. The reaction product was further purified by reverse-phase preparative HPLC (20% aqueous acetonitrile, 3.0 mL/min) to yield of 4.5 mg of **4**. The ^{1}H NMR spectrum of the reduced product is identical to that of **4** isolated from the *A. oryzae* transformant (Figure S22).

Purification condition for compound **6′**:

The extract of *A. oryzae*/*mfmBACDEFGJ* cultivated on 100 DPY agar plates (0.47 g) was subjected to open silica gel chromatography and eluted stepwise using hexane:acetone gradient (9:1 to 7:1). Fractions containing **6** (96.0 mg) were then concentrated and reduced using 1.0 g of $NaBH_4$. The reaction product was further purified by open silica gel chromatography (hexane aacetone15:1) to yield 6.8 mg of **6′**.

Purification condition for compound **7′**:

The extract of *A. oryzae*/*mfmBACEFD+ocdTC* cultivated on 200 DPY agar plates (2.4 g) was subjected to open silica gel chromatography and eluted stepwise using hexane:acetone gradient (9:1 to 2:1). Fractions containing **7** (82.3 mg) were then concentrated and reduced using 0.2 g of $NaBH_4$. The reaction product was further purified by open silica gel chromatography (hexane acacetone:1) to yield 19.2 mg of **7′**.

Purification condition for compound **8′**:

The extract of *A. oryzae*/*mfmBACEF+psetPT* cultivated on 100 DPY agar plates (0.47 g) was subjected to open silica gel chromatography and eluted stepwise using hexane:acetone gradient (9:1 to 1:1). Fractions containing **8** (33.0 mg) were then concentrated and reduced using 0.6 g of $NaBH_4$. The reaction product was further purified by reverse-phase preparative HPLC (40% aqueous acetonitrile, 3.0 mL/min) to yield 2.9 mg of **8′**.

Purification condition for compound **9′**:

The extract of *A. oryzae*/*mfmBACEF+psetPT* (Y251G+C348Y) cultivated on 150 DPY agar plates (1.38 g) was subjected to open silica gel chromatography and eluted stepwise using hexane:acetone gradient (9:1 to 1:1). Fractions containing **9** (25.0 mg) were then concentrated and reduced using 0.4 g of $NaBH_4$. The reaction product was further purified by reverse-phase preparative HPLC (56% aqueous acetonitrile, 3.0 mL/min) to

yield 2.5 mg of **9′**.

X-ray crystallographic analysis

A single crystal of **1′** was grown in acetonitrile by a slow evaporation process at 4 °C. Single crystal X-ray diffraction measurement was performed on a Bruker D8 Venture diffractometer using Cu Kα radiation at 193 K. The data collection was performed with the APEX3 program, and cell refinement and data reduction were carried out using the SAINT program. The structure of **1′** was solved by direct method with the SHELXT program and refined using the SHELXL program. All non-hydrogen atoms were refined anisotropically, whereas hydrogen atoms were placed by geometrical calculations. The absolute configuration of **1′** was determined by the Flack parameter.

Analytical data

Compound 1′. Colorless crystal; $[\alpha]^{24}_{D}$ +40.4 (*c* 1.0, $CHCl_3$); for NMR data, see Figure S9 to Figure S15; HRMS (ESI) *m/z*: $[M + H]^+$ Calcd for $C_{26}H_{39}O_6$ 447.2741; Found 447.2748.

5,7-Dihydroxy-4-(hydroxymethyl)-6-methylphthalide (3). White amorphous solid; for NMR data, see Figure S16 to Figure S20; HRMS (ESI) *m/z*: $[M + H]^+$ Calcd for $C_{10}H_{11}O_5$ 211.0601; Found 211.0599.

5-Hydroxy-4-(hydroxymethyl)-7-methoxy-6-methylphthalide (4). White amorphous solid; ^1H NMR (CD_3OD, 400 MHz): δ 5.29 (s, 2H), 4.74 (s, 2H), 3.94 (s, 3H), 2.15 (s, 3H); $^{13}C\{^1H\}$ NMR (CD_3OD, 100 MHz): δ 171.7, 162.1, 158.6, 147.6, 120.1, 117.4, 109.2, 69.9, 62.5, 58.8, 8.8; for NMR spectra, see Figure S21 and Figure S23; HRMS (ESI) *m/z*: $[M + H]^+$ Calcd for $C_{11}H_{13}O_5$ 225.0757; Found 225.0753. The NMR data are in good agreement with the reported data.[12]

Compound 6′. Colorless oil; for NMR data, see Figure S24 to Figure S29; HRMS (ESI) *m/z*: $[M + H]^+$ Calcd for $C_{26}H_{37}O_5$ 429.2636; Found 429.2624.

Compound 7′. Colorless oil; $[\alpha]^{23}_{D}$ +11.2 (*c* 1.0, $CHCl_3$); for NMR data, see Figure S30 to Figure S36; HRMS (ESI) *m/z*: $[M + Na]^+$ Calcd for $C_{26}H_{38}O_6Na$ 469.2561; Found 469.2567.

Compound 8′. White amorphous solid; for NMR data, see Figure S37 to Figure S42; HRMS (ESI) *m/z*: $[M + H]^+$ Calcd for $C_{16}H_{21}O_5$ 293.1384; Found 293.1384.

Compound 9′. White amorphous solid; for NMR data, see Figure S43 to Figure S48; HRMS (ESI) *m/z*: $[M + H]^+$ Calcd for $C_{21}H_{29}O_5$ 361.2010; Found 361.2020.

*Crystallographic data for **1′**.* $C_{26}H_{38}O_6$, M = 446.56, a = 9.061(12) Å, b = 11.978(12) Å, c = 12.657(16) Å, α = 107.62(4)°, β = 110.74(4)°, γ = 93.69(4)°, V = 1202(3)Å^3, T = 193(2) K, space group $P1$, Z = 2, μ(Cu Kα) = 0.697 mm^{-1}, 39 390 reflections measured, 9406 independent reflections (R_{int} = 0.0629). The final R_1 values were 0.0454 ($I > 2\sigma(I)$). The final $wR(F^2)$ values were 0.1164 ($I > 2\sigma(I)$). The final R_1 values were 0.0498 (all data). The final $wR(F^2)$ values were 0.1216 (all data). The goodness of fit on F^2 was 1.051. Flack parameter –0.06(8). The crystallographic information file (CIF) for this crystal structure was submitted to The Cambridge Crystallographic Data Centre (CCDC) under reference number 2293718.

Table S1. Terpene cyclase homologues identified in this study and used to create the phylogenetic tree in Figure S1.

**Locus tag**<br>**Accession**<br>**Origin**	**Protein sequence**
F5B19DRAFT_81170<br>KAI1087245.1<br>*Rostrohypoxylon terebratum* CBS 119137	MLSNFHLPLNPIDKVNQPPVSYLQVQDGLILCSGLCWTIAYILYIRQAYRDKSYGMPLVCLCANIAWEFVFGAAIPESAAQVV<br>SFFPWFVIDIGIVYTTWKFGREQWKHAPLVAQNLGWILLGGITGMLVMFWTFLKTYNNRYEAGFYLAWTDQIIVSTTSVAQL<br>MSRNNTSGHSWGIWFTRWIGSVFAELIFVWRYWNYPESYPVAATHVTIFLFVVTELADLTYPFVYASLDKKEKKKLK*
F4806DRAFT_52672<br>KAI0896154.1<br>*Annulohypoxylon nitens* CBS 120705	MLSNFHLPLNPIDRVNQPPVSYLQVQDGLILCSGLCWTTAYILYIRQAYRDKSYGMPLVCLCANIAWEFVFGAAIPESAAQV<br>VSFFPWFVIDIGIVYTTWKFGREQWKHAPVVAQNLGWILIGGITGMLVLFWTFLKTYDNRYEAGFYLAWTDQIIVSTTSVAQ<br>LMSRNNTSGHSWGIWFTRWIGSVFAELIFVWRYWNYPESYPVAATHVTIFLFVVTELADLTYPFVYASLEKQEKKKLK*
F4805DRAFT_30178 (MfmH)<br>KAI1452419.1<br>*Annulohypoxylon moriforme* CBS 123579	MFSNLHLPLNPIDKVNQPPVYYLQVQDGLIICSGLCWTTAYILYIRQAYRDKSYGMPLVCLCANIAWEFLFGAAIPESAAQVV<br>SFFPWFVIDIGIVYTTWKFGREQWKHAPLVAQNLGWILLGGIAGMLVMFWAFLKTYDNRYEAGFYLAWTDQIIVSTTSVAQL<br>MSRNNTSGHSWGIWFTRWIGSVFAELIFVWRYWNYPESYPVAATHVTIFLFIVTEVADLTYPFVYASLDKKEKKKLK*
GGS22DRAFT_19057<br>XP_047817924.1<br>*Annulohypoxylon maeteangense* CBS 123835	MFSNLHLPLNPIDKVNQPPVSYLQVQDGLIICSGLCWTTAYILYIRQAYRDKSYGMPLVCLCANIAWEFLFGAAIPESAAQVV<br>SFFPWFVIDIGIVYTTWKFGREQWKHAPLVARNLGWILLGGIAGMLVMFWAFLKTYDNRYEAGFYLAWTDQIIVSTTSVAQL<br>MSRNNTSGHSWGIWFTRWIGSVFAELIFVWRYWNYPESYPVAATHFTIFLFVLTEVADLTYPFVYASLEKKEKKKLK*
F4807DRAFT_439860<br>XP_047850900.1<br>*Annulohypoxylon truncatum* CBS 140777	MLSDFHLPLNPIDKVNQPPVHYLQVQDGLILCSGLCWTTAYILYIRQAYRDKSYGMPLVCLCANIAWEFLFGAAIPESAAQV<br>VSFFPWFVIDIGIVYTTWKFGREQWKHAPLVAQNLGWILLGGITGMLVMFWAFLKTYDNQYEAGFYLAWTDQIIVSTTSVAQ<br>LMSRNNTSGHSWGIWFTRWIGSVFAELIFVWRYLNYPESYPVAATHMTIFLFVVTEVADLTYPFVYASLERKEKKKLK*
F4781DRAFT_40845<br>KAI2465131.1<br>*Annulohypoxylon bovei var. microspora* CBS 124037	MLSNFHLPLNPIDKANQPPVHYLQVQDGLIISSGLCWTTAYILYIRQAYRDKSYGMPLVCLCANIAWEFLFGAVIPDSAAQVV<br>SFFPWFVIDVGIVYTTWKFGRDQWKHAPLVAQNLGWILVGGIIGMLVMFWTFLKTYDNQYEAGFYLAWTDQLVVSTTSVAH<br>LMSRNSTSGHSWGIWFARWIGSVFAELVFVWRYVNYPESYPVAATHVTIFLFVMTEVVDLTYPFVYASLEKREKIKLK*
F4776DRAFT_677579<br>KAI0139589.1<br>*Hypoxylon* sp. NC0597	MLSEFHLPLNPIDKVNQPPVSYLQVQDSLIISSGLCWTTAYVLYIRQAYRDKSYGMPLVCLCANIAWEFLFGAVIPDSAAQVV<br>SFFPWFVIDVGIVYTTWKFGREQWKHAPLVAQNLGFILIGGIVGMLVMFWTFIKTYDNLYEAGFYLAWTDQLVVSTTSVAHL<br>MSRHNTSGHSWGIWFARWIGSVFAELVFIWRYVNYPESYPVVATPFTVFLFVMTEVVDLTYPFVYASLQKKEKGKLN*
F5Y13DRAFT_183420<br>KAI1407369.1<br>*Hypoxylon* sp. FL1857	MLSEFHLPLNPIDRVNQPPVSYLQIQDGLIIASGLCWTTAYILYIRQAYRDKSYGMPLVCLCANIAWEFLFGAVIPDSAAQVVS<br>FFPWFVIDVGIVYSTWKFGREQWKHAPFVAQNLGFILIGGIVGMLGMFWTFIKTYDNLYEAGFYLAWTDQLVVSTTSVAQL<br>MSRHNTSGHSWGIWLARWIGSVFAELVFVWRYVNYPESYPVAAAPFTVFLFVMTEVADLTYPFVYASLQKKEKNKLN*
HO133_005438<br>XP_037148330.1<br>*Letharia lupina* WasteWater1	MPFHLPLSPLDRRNQPPVYYLQVQDALILISSFLWTIAYALYVRQAFRDKSYGMPLLCLWANIAWEFLFGVVAPTSAAQTIAF<br>VPWLLIDVAIVYTTWRFGPDQWKRSPLVANNMGWIVTSGTILMTGLFWTFIKTVGVEAASFYIGYGDQLLISITSVAQLMSR<br>NNTSGHSLGIWFYRASGTCLTILLFAWRYWHYADSYPRVAQPTTLFLFVGSEVVDSIYPMVYVFLEKKAKKKGT*
G7Y79_00010g027750<br>KAG7007274.1<br>*Physcia stellaris* C0375214F	MPYHLPLGPVDRVNQPPVYYLQVQDSLILISSWLWTVAYVLYVRQAFRDKSYGMPLLCLWANIAWEFLFGVWAPTSAAQT<br>VAFVPWLIIDIGIVYTTWKFGPEQFKAAPMVANNMGAVVFGGTAAMTVLFWTFIKTFGVDNASFYIGYGDQLLISITSVAQLM<br>SRHNTSGHSLGIWFCRASGTFLTILLFGWRYWHYAESYPRVAYPTTLFLFAAAEVVDGFVYPMVYFRLENGKKSKRKIK*
B0I35DRAFT_436133<br>KAH7313938.1<br>*Stachybotrys elegans* MPI-CAGE-CH-0235	MQLHLPLSPVDTKNQPPLYYLQVQDGLILLQSVLWTIAYILYVRQGFIDSSYGMPIVCLCANIAWEFLFGLVVPVSAAQTVAF<br>VPWLIIDIGIVYTTWKYGKHQWKSSPKIANNLGIILTFGIWFMIVCFWCLIKTIGLNASSFYLGYGLQLLIGTSSVAQLLSRNNT<br>SGHSWGIWFTRATGTFFTIVLFGWRYAHYPDSYPRVAEPMTLFLFGTSELFDVIYPFVYYCVDGTKKDKTKRG*
OIDMADRAFT_101952<br>KIN05208.1<br>*Oidiodendron maius* Zn	MALHLPLNPIDRANRPPAYYLTVQDGLILLSSALWTIAYVLYVRQGLLDRSYGMPIVPLCADIVWEFLFGLIVPTSVAQVVAFT<br>PWLIIDIGIVYTTYQFGPEQWAHAPLVAENLGWILILGVLFTMALFWAIIKTIGVDNSSFYIAYVVQLVISSYSVAQLISRGNTSG<br>HSWSIWFFRTTGTAATIAQFIWKYSHYPKSYPRVARPLTQFLFISCTMIDLIYPFVYMAVEQREGRNLKL*
CI238_03399[a]<br>KZL80443.1<br>*Colletotrichum incanum* MAFF 238704	MGFHLPLNRIDKANQPPLYYLQVQDGLILSVSIFWTIAYVLYIRQGLRDKSYGMPLFALCANIAWEFLFGVVMPTSAAQVVAF<br>VPWLVIDIGIIYTTWKYGPQEWKHSPIVARNLGWILSLGVVTMIGAFWAFIDTVGIDPASFYLGYSDQFLISCTSLVQLLRRNS<br>TAGHSWGIWFNRTFGTFLSMVLFAWRYAFYPGSYPRVAQPIVVFFFVASEVLDVAYAFVYSHIAAQERLKQK*
SETTUDRAFT_36580<br>XP_008021319.1<br>*Exserohilum turcica* Et28A	MGFHLPLNKIDQVNQPPLYFLQVQDGLILSVSISWTIAYILYIRQGLRDKSYGMPLFALCGNVAWELLFGVVMPTSAAQVVAF<br>VPWLAIDVGIIYTTWKFGPEEWKNSPLVARNLRWILLLGIAITTGAFWAFIKTVGIDPASFYLGYSDQFLISCTSLVQILRRGST<br>AGHSWGIWFYRTFGTFLSIVLFAWRYYFYPQSYPRVAEPIVVFFFFASEVLDVSYAFVYYHIEKQNKLKKN*
J7T55_011203<br>XP_052988876.1<br>*Diaporthe amygdali* CAA958	MSYHLPLSPVDRAIQPPVYYLQVQDFLILNVSIFWTIAYVLYVRQGYRDKSYGMPLFALSGNIAWEFLFGVAMPTSVAQVVC<br>FVPWLVIDIFIIHTTWKYGPHQWKQSPVVANNIGSILVFGVLFMTGAFYFFIKTVGLDAASFYLGYSDQLLISATSLAQLLRRN<br>STSGHSWGIWFCRTFGTFLSIVLFSWRYYHYPGSYPRVAEPIVVFFFVASEVIDISYAFVYAHIDRKEKAALKEKSKRK*
CORC01_00031[a] (OcdTC)<br>XP_022481694.1<br>*Colletotrichum orchidophilum* IMI 309357	MPYHLPLSPIDRAIQPPVYYLQVQDSLILSVSVFWTIAYVLYVRQGYRDKSYGMPLFALAGNIAWEFLFGVAMPTSVAQVVC<br>FVPWLVIDVFIVHTTWKYGARQFKQSPVVAKNLGLVLVFGVSFVTASFYFFIKTVGLDAASFYLGYSDQLLISITSVAQLLRRN<br>NTLGHSWGIWFCRTFGTFLSIVLFCWRYFHYPHSYPRVAEPIVVFFFVASEVFDVLYAFIYAHIERVEKGAIEEKSR*
F5883DRAFT_475157<br>KAH8746268.1<br>*Diaporthaceae* sp. PMI_573	MPYHLPLSPVDRAIQPPVYYLQVQDSLILSVSIFWTIAYVLYVRQGYRDKSYGMPLFALAGNIAWEFLFGVAMPTSVAQVVC<br>FVPWLVIDVFIVHTTWKYGGRQFKQSPVVANNLGLVLVFGVAFVTAAFYFFIKTVGLEAASFYLGYSDQLLISITSLAQLLRRN<br>NTLGHSWGIWFCRTFGTFLSIVLFCWRYFHYPDSYPRVAEPIVVFFFATSEVFDILYAFVYAHIARKEKAAVKEKSK*
INS49_009288[a]<br>XP_043024587.1<br>*Diaporthe citri* NFHF-8-4	MPYHLPLSPVDRAIQPPVYYLQVQDSLILSVSIFWTIAYVLYVRQGYRDKSYGMPLFALAGNIAWEFLFGVAMPTSVAQVVC<br>FVPWLVIDVFIVHTTWKYGARQFKQSPVVANNIGLVLAFGVAFVTASFYFFIKTVGLDAASFYLGYSDQLLISITSLAQLLRRN<br>NTLGHSWGIWFCRTFGTFLSIVLFCWRYFHYPDSYPRVAEPIVVFFFAASEVFDILYAFVYAHIERKEKAAVKEKSR*
KVR01_002063[a]<br>XP_044647078.1<br>*Diaporthe batatas* CRI 302-4	MPYHLPLSPVDRAIQPPVYYLQVQDSLILSVSIFWTIAYILYVRQGYRDQSYGMPLFALAGNIAWEFLFGVAMPTSVAQVVC<br>FVPWLVIDVFIVHTTWKYGARQFKNSPVVANNIGLLLAFGVAFVTGSFYFFIKTVGLDAASFYLGYSDQLLISITSVAQLLRRN<br>NTLGHSWGIWFCRFFGTFLSIVLFCWRYFHYPDSYPRVAEPIVVFFFAASEVFDILYAFVYAHIERTEKAAIKGKSKQK*
MMC15_002493[a]<br>MCJ1317170.1<br>*Xylographa vitiligo* T1866	MQLHLPLSPTDKVLQPPASYLIVQDWTLLYCMFGWTVSYILYVRQAFRDKSYGMPIANVCANIAFEFIFGVVIPSSVAQVVC<br>FVPWLVIDVGIVYSVVTFGPNVWKASPLVANNLGVIMAAGILSCMVMFWTVIETMGKDDAAFYLAYGDELVISTLSIAHLLKR<br>GNTDGHSLGIWFWRATATIVAVFAFEWRYWHYPEAHARVGQPFSTFLFVSFTLLDVIYLVLYLSLERKRKSKMK*
F4822DRAFT_399218<br>XP_051315447.1<br>*Hypoxylon trugodes* CBS 135444	MGLHLPLSPNDRVLRPPLYYLQVQDGLILGSGAMWTITYILYVVQAYRDKSYGMPLVSLCANISWELLYGVVYPTSVGQFV<br>AFTSWLITDIGIVYVTIKFGPSQWKQAPMVANNLGLLLTAGIGTMIAMHLAFIKQIPSLDECALWSAFVCQVLLSGASVLHLM<br>CRDNTSGHSWTIWLIRWLGTVLVIGLFVWRWVHYPETYPITAKPVTVFLFVLTEIADIIYPFVYYAVEKKEKLRKQVKQVKSK*
RAG0_16103[a]<br>CZT12178.1<br>*Rhynchosporium agropyri* 04CH-RAC-A.6.1	MVRAYLSPVDKVNKPSLRYLQVQDFFVLGSGIPWTIAYILYARQANIDKSYGMPLIPLCANIAWEFIYGVIHPNSLGQVISFVP<br>WLIADVPIVYWTLKHGPSKWEQAPLVADNLGLILTVGIAMMLAMHLAFRRSCKNIEDGPFWSAWVCQLLISCGSVMHLMCR<br>NETSGHSWGIWVCRWLGTLSAISLFAWRYAHYPKDYPMAAKPMALFIFIVSEAADLIYPFVYASLESKHGIV*
RCO7_07690[a]<br>CZT12485.1<br>*Rhynchosporium commune* UK7	MVRVYLSPVDKVNKPSLRYLQVQDFFVLGSGIPWTFAYILYARQANIDKSYGMPLIPLCANIAWEFIYGVIHPNSLGQVISFV<br>PWLIADVPIVYWTLKHGPSKWEQAPLVADNLGLILAVGIAMMLAMHLAFRRSCKNIEDGPFWSAWVCQLLISCGSVMHLM<br>CRNETSGHSWGIWVCRWLGTLSAISLFAWRYAHYPKDYPMAAKPMALFIFIVSEAADLIYPFVYASLESKHGIV*
RSE6_10008[a]<br>CZT49202.1<br>*Rhynchosporium secalis* 02CH4-6a.1	MVRVYLSPVDKINKPSLRYLQVQDFFVLGSGIPWTIAYILYARQANIDKSYGMPLIPLCANIAWEFIYGVIHPNSLGQVISFVP<br>WLIADVPIVYWTLKHGPSKWEQAPLVADNLGLILAVGIAMMLAMHLAFRRSCKNIEDGPFWSAWVCQLLISCGSVMHLMC<br>RNETSGHSWGIWVCRWLGTLSAISLFAWRYAHYPKDYPMAAKPMALFIFIVSEVADLIYPFVYASLESKHGIV*

[a]These sequences were manually revised in this study.

Table S2. Annotation of each gene in the *mfm* cluster from *Annulohypoxylon moriforme* CBS 123579, as well as *ocdTC* and *psetPT*.

The *mfm* cluster from *Annulohypoxylon moriforme* CBS 123579
ca . 30.8 kb

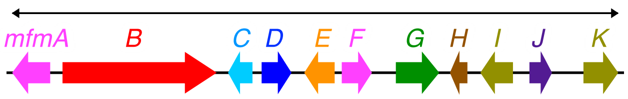

Gene	Sequence ID[a]	Amino acids (base pairs)	Protein homologue (origin)	Similarity/Identity (%)	Proposed function
mfmA	KAI1452412.1	515 (1879)	TropD (*Talaromyces stipitatus*)	73/60	cytochrome P450 monooxygenase
mfmB	KAI1452413.1	2493 (7766)	AndM (*Emericella variecolor*)	44/61	non-reducing polyketide synthase (SAT-KS-AT-PT-ACP-CMeT-TE)
mfmC	KAI1452414.1	377 (1192)	MpaDE (*Penicillium roqueforti*)	52/67	metallo-hydrolase
mfmD	KAI1452415.1	442 (1469)	PhnF (*Penicillium herquei*)	54/70	DMATS-type prenyltransferase
mfmE	KAI1452416.1	401 (1493)	Fma-MT (*Aspergillus fumigatus*)	35/49	methyltransferase
mfmF	KAI1452417.1	493 (1482)	Hkm5 (*Aspergillus hancockii*)	28/44	cytochrome P450 monooxygenase
mfmG	KAI1452418.1	604 (2149)	PatE (*Aspergillus clavatus*)	43/60	glucose-methanol-choline family oxidoreductase
mfmH	KAI1452419.1	242 (841)	MacJ (*Penicillium terrestre*)	36/54	Pyr4-family terpene cyclase
mfmI	KAI1452420.1	472 (1605)	StrL (*Stachybotrys* sp.)	26/48	transcription factor
mfmJ	KAI1452421.1	344 (1100)	VirL (*Trichoderma virens*)	42/59	short-chain dehydrogenase/reductase
mfmK	KAI1452422.1	462 (1714)	BasR (*Aspergillus nidulans*)	41/59	transcription factor
ocdTC[b]	XP_022481694.1	243 (868)	MacJ (*Penicillium terrestre*)	33/53	Pyr4-family terpene cyclase
psetPT[b]	XP_031913546.1	433 (1302)	PhnF (*Penicillium herquei*)	57/72	DMATS-type prenyltransferase

[a]Sequence IDs are as designated in the NCBI database. [b]The sequences of these genes were manually revised (see Table S3).

Table S3. Revised DNA and protein sequences. Highlighted in magenta are the regions predicted for an intron in this study.

Gene	Revised DNA sequence	Prevised protein sequence
ocdTC	atgccgtaccacctccccttaagcccgatcgatcgagctattcagcctccagtgtactacttgcaagtccaagattcactaatcttgagcgtatcggttttctggaccatcgc<br>ctatgtcctctacgtacgccaggggtaccgcgacaagtcctatggcatgccactttttgccctgtacgttgttgggatatgcccaggatatgttaccgatcttggtgattgacta<br>tcatatagggcgggaaatatagcatgggaatttctctttggcgtggcgatgcccacgtctgtagcccaggtggtttgcttcgtaccatggttggttattgacgtctttatcgtcca<br>caccacatggaagtatggagcgcgccagttcaaacagtctcctgtagtcgccaaaaaccttgggctcgtcttggttttcggggtcagctttgtgactgcgtccttctacttctt<br>catcaagaccgtcggactggatgcggcttcgttttaccttggttactccgaccagctcctaattagtatcacgtccgtggcacagcttctgaggcgaaataacacccttgga<br>cactcctggggtatttggtaagttgtacctagacaagtcgaggaatgatatggcatcagcatatatgcgctctaactaacggttcatatacaggttctgtcgaacatttggca<br>catttctctctattgtattgttctgctggcggtactttcactatcctcatagctaccctagggtcgcagagccgatagtcgttttcttcttcgtggcgtcagaagtttttgatgttttatac<br>gcatttatatatgcacatatcgaacgggtagaaaagggcgctatagaagagaaaagcagataa	MPYHLPLSPIDRAIQPPVYYLQVQDSLILSVSVFW<br>TIAYVLYVRQGYRDKSYGMPLFALAGNIAWEFLF<br>GVAMPTSVAQVVCFVPWLVIDVFIVHTTWKYGAR<br>QFKQSPVVAKNLGLVLVFGVSFVTASFYFFIKTVG<br>LDAASFYLGYSDQLLISITSVAQLLRRNNTLGHSW<br>GIWFCRTFGTFLSIVLFCWRYFHYPHSYPRVAEPI<br>VVFFFVASEVFDVLYAFIYAHIERVEKGAIEEKSR*
psetPT	atgtccgtcaccgttgaactacccacattccagtctcttgggcgcgagagcccggcctcagaaggcctgccctactggtggctgactagtggacgcgatcttgctcgcat<br>gctccaagaggcacagtaccctgaagatacacagcgccagttcctcctctttttcagagacacgatctgcccccagctcggaggccgccctgaagctgattcactccgc<br>agtggcgtcggctgggatggcaacccccttcgaatacagttttgaactcaagagctctggcaaggagcaagcagttcggtttgtcgtagacgtcagcccactacgacccg<br>ccgatgatgccaacccccctcagcatcaagaattttgagattgtccttgacgcattagcgaagaagactccaggattcgacgacacctggtacgcatccctgcggaagtg<br>gttcgtgcattccgatcgctcgaccgaggaacagaagagtcttgtcgcacaggcagggtaccagatgcccatgatcctcgggtttgacattcgccgacgtctccccagc<br>ccagatgccatcccggtcatggcgaaggtctatttcccaccctgtttcacggcggtggcggaaggaattacacgatgggaggccgtgcgacgtggcgtgtatcaactgc<br>caaacatcgaatcccatcccaacatccttcgctctctggagctcattcaacaatacctggacagcaagccgaaagagtatgaaaacggtccgcgctatctggcgaccg<br>actttgtcaccaccgaccaggcgcgtctgaagatctacatgcgacatcccgccgaatcattcgaggatatctgggattactataccctcgggggaaggatcccaggctt<br>agatgaggataaggagaaattccgcgagctcatgtcgctcaccgcctataatccagaccctacgccggcacaggacggtggccaaccacactataccgcggtgcag<br>agaaaaatgacggcgatttacttctccctctccacggataacccaacgcccgcgccgaagatctgcttctaccctgcgaactttgcagccaatgatgaggtcataggtgc<br>aggtgtggatcagtggttgcaaaaatatggatgggatgacggaagtaagccgatgaaggagaaagtccgcagtgtcttgtaagtggtccacttcaatttccttgctgctg<br>agcttttgctgttatatgaacatacactaaagcatgcataaatagtactcatcgtaatctgtccgataagaagggtatattcacctttctggggatcgggagaaaggaggat<br>ccaaccaagaaagagctcagcatgcaggtttacgtgactggggaattgtatacaaccccacgaatttag	MSVTVELPTFQSLGRESPASEGLPYWWLTSGRD<br>LARMLQEAQYPEDTQRQFLLFFRDTICPQLGGR<br>PEADSLRSGVGWDGNPFEYSFELKSSGKEQAVR<br>FVVDVSPLRPADDANPLSIKNFEIVLDALAKKTPG<br>FDDTWYASLRKWFVHSDRSTEEQKSLVAQAGYQ<br>MPMILGFDIRRRLPSPDAIPVMAKVYFPPCFTAVA<br>EGITRWEAVRRGVYQLPNIESHPNILRSLELIQQY<br>LDSKPKEYENGPRYLATDFVTTDQARLKIYMRHP<br>AESFEDIWDYYTLGGRIPGLDEDKEKFRELMSLT<br>AYNPDPTPAQDGGQPHYTAVQRKMTAIYFSLSTD<br>NPTPAPKICFYPANFAANDEVIGAGVDQWLQKYG<br>WDDGSKPMKEKVRSVFTHRNLSDKKGIFTFLGIG<br>RKEDPTKKELSMQVYVTGELYTTPRI*

Table S4. Primers used in this study.

Primer	Sequence (5′ to 3′)
mfmA-F	TCGAGCTCGGTACCCTGATTGAAGCCATGGACAAG
mfmA-R	CTACTACAGATCCCCTATACCTGCTGGTTTTTCACC
mfmB-F	TCGAGCTCGGTACCCGATGACTCAATTCGGCACTTCG
mfmB-R	CTACTACAGATCCCCACTCAATCACTCAAACCAAC
mfmC-F	TCGAGCTCGGTACCCATGGTGACTAAGCCAACAACCC
mfmC-R	CTACTACAGATCCCCGGTGTCTAGCTACACTTTAAGCTCG
mfmD-F	TCGAGCTCGGTACCCATGACTGCTGCAGTCCAATCC
mfmD-R	CTACTACAGATCCCCGGTACATTTAGAAGCGTGGTGTTAC
mfmE-F	TCGAGCTCGGTACCCGAGCATGGCATCCACTAC
mfmE-R	CTACTACAGATCCCCTATTGATCAAGTTCGGCCTC
mfmF-F	TCGAGCTCGGTACCCAAGATGTGGTCGCTGATTCC
mfmF-R	CTACTACAGATCCCCTCCGCGCATCTCGGTGTATCTAAC
mfmG-F	TCGAGCTCGGTACCCATGTATATGCTCCGTCCTAGC
mfmG-R	CTACTACAGATCCCCATTCTATCTAGTACTTCGTCTGC
mfmH-F	TCGAGCTCGGTACCCATGTTCTCCAATCTCCATCTCCC
mfmH-R	CTACTACAGATCCCCGGCGAATACCACCAATGATTACTTC
mfmJ-F	TCGAGCTCGGTACCCATGGTTTCTTCAAAGGAGCTACCG
mfmJ-R	CTACTACAGATCCCCTAAGATTCAATTTCCTTTCATGGAC
ocdTC-F	TCGAGCTCGGTACCCATGCCGTACCACCTCCCCTTAAG
ocdTC-R	CTACTACAGATCCCCTTTATCTGCTTTTCTCTTCTATAGCGC
psetPT-F	TCGAGCTCGGTACCCATGTCCGTCACCGTTGAACTAC
psetPT-R	CTACTACAGATCCCCGCTGCTAAATTCGTGGGGTTG
mfmD_G257Y-F	GAGCACGCTATCTGTCAACAGATTTCGTC
mfmD_G257Y-R	TTGACAGATAGCGTGCTCCACCCGCAAG
mfmD_Y357C-F	CAAAGTCTGTTTTTACCCGGCTCGTG
mfmD_Y357C-R	GTAAAAACAGACTTTGGGGATCGGATATG
psetPT_Y251G-F	TCCGCGCGGTCTGGCGACCGACTTTG
psetPT_Y251G-R	GCCAGACCGCGCGGACCGTTTTCATACTC
psetPT_C348Y-F	AAGATCTACTTCTACCCTGCGAACTTTGC
psetPT_C348Y-R	GTAGAAGTAGATCTTCGGCGCGGG
InF-linker-F1	GCTCGCGAGCGCGTTCCACTGCATCATCAGTCTAG
InF-linker-R1	AACGCGCTCGCGAGCAAGTACCATACAGTACCGCG
InF-linker-F2	TCGCGTGCGCGTTTACCCATCATGGTGTTTTGATC
InF-linker-R2	TAAACGCGCACGCGACATTAATCCGGATCCTTTCC
InF-pAdeA_XbaI-F	GCAGGTCGACTCTAGCCCATCATGGTGTTTTGATC
InF-pAdeA_XbaI-R	TAGTAGATCCTCTAGGTAAGATACATGAGCTTCGG
InF-pAdeA_SpeI-F	TAGAGGATCTACTAGTCAAGAGCAGAATGTGAACG
InF-pAdeA_SpeI-R	AATCCATATGACTAGTGATACATGAGCTTCGGTG
InF-pUSA_BamHI-F	TTATAGGAAAGGATCCCCATCATGGTGTTTTGATC
InF-pUSA_BamHI-R	TGACTCTAGAGGATCGTAAGATACATGAGCTTCGG

Table S5. Plasmids constructed in this study and PCR conditions for the amplification of the inserts for the plasmid constructions.

**Plasmid**	**Inserts**	**Primer 1**	**Primer 2**	**PCR Template**	**Vector**
pPyrG-mfmA	*mfmA*	mfmA-F	mfmA-R	gDNA	pPyrG digested with *Sma*I
pTAex3-mfmB	*mfmB*	mfmB-F	mfmB-R	gDNA	pTAex3 digested with *Sma*I
pTAex3-mfmC	*mfmC*	mfmC-F	mfmC-R	gDNA	pTAex3 digested with *Sma*I
pTAex3-mfmD	*mfmD*	mfmD-F	mfmD-R	gDNA	pTAex3 digested with *Sma*I
pTAex3-mfmE	*mfmE*	mfmE-F	mfmE-R	gDNA	pTAex3 digested with *Sma*I
pTAex3-mfmF	*mfmF*	mfmF-F	mfmF-R	gDNA	pTAex3 digested with *Sma*I
pTAex3-mfmG	*mfmG*	mfmG-F	mfmG-R	gDNA	pTAex3 digested with *Sma*I
pTAex3-mfmH	*mfmH*	mfmH-F	mfmH-R	gDNA	pTAex3 digested with *Sma*I
pTAex3-mfmJ	*mfmJ*	mfmJ-F	mfmJ-R	gDNA	pTAex3 digested with *Sma*I
pTAex3-ocdTC	*ocdTC*	ocdTC-F	ocdTC-R	gDNA	pTAex3 digested with *Sma*I
pTAex3-psetPT	*psetPT*	psetPT-F	psetPT-R	gDNA	pTAex3 digested with *Sma*I
pPyrG-mfmA+C	*PamyB-mfmC-TamyB*	InF-pUSA_BamHI-F	InF-pUSA_BamHI-R	pTAex3-mfmC	pPyrG-mfmA digested with *Bam*HI
pPyrG-mfmA+C+E	*PamyB-mfmC-TamyB*<br>*PamyB-mfmE-TamyB*	InF-pUSA_BamHI-F<br>InF-Linker-F1	InF-Linker-R1<br>InF-pUSA_BamHI-R	pTAex3-mfmC<br>pTAex3-mfmE	pPyrG-mfmA digested with *Bam*HI
pPyrG-mfmA+C+E+F	*PamyB-mfmC-TamyB*<br>*PamyB-mfmE-TamyB*<br>*PamyB-mfmF-TamyB*	InF-pUSA_BamHI-F<br>InF-Linker-F1<br>InF-Linker-F2	InF-Linker-R1<br>InF-Linker-R2<br>InF-pUSA_BamHI-R	pTAex3-mfmC<br>pTAex3-mfmE<br>pTAex3-mfmF	pPyrG-mfmA digested with *Bam*HI
pPyrG-mfmA+C+D	*PamyB-mfmC-TamyB*<br>*PamyB-mfmD-TamyB*	InF-pUSA_BamHI-F<br>InF-Linker-F1	InF-Linker-R1<br>InF-pUSA_BamHI-R	pTAex3-mfmC<br>pTAex3-mfmD	pPyrG-mfmA digested with *Bam*HI
pPyrG-mfmA+C+D+E	*PamyB-mfmC-TamyB*<br>*PamyB-mfmD-TamyB*<br>*PamyB-mfmE-TamyB*	InF-pUSA_BamHI-F<br>InF-Linker-F1<br>InF-Linker-F2	InF-Linker-R1<br>InF-Linker-R2<br>InF-pUSA_BamHI-R	pTAex3-mfmC<br>pTAex3-mfmD<br>pTAex3-mfmE	pPyrG-mfmA digested with *Bam*HI
pAdeA-mfmF+G	*PamyB-mfmF-TamyB*<br>*PamyB-mfmG-TamyB*	InF-pAdeA_XbaI-F<br>InF-Linker-F1	InF-Linker-R1<br>InF-pAdeA_XbaI-R	pTAex3-mfmF<br>pTAex3-mfmG	pAdeA digested with *Xba*I
pAdeA-mfmF+G+H+J	*PamyB-mfmH-TamyB*<br>*PamyB-mfmJ-TamyB*	InF-pAdeA_SpeI-F<br>InF-Linker-F1	InF-Linker-R1<br>InF-pAdeA_SpeI-R	pTAex3-mfmH<br>pTAex3-mfmJ	pAdeA-mfmF+G digested with *Spe*I
pAdeA-mfmF+G+H	*PamyB-mfmH-TamyB*	InF-pAdeA_SpeI-F	InF-pAdeA_SpeI-R	pTAex3-mfmH	pAdeA-mfmF+G digested with *Spe*I
pAdeA-mfmG+H	*PamyB-mfmG-TamyB*<br>*PamyB-mfmH-TamyB*	InF-pAdeA_SpeI-F<br>InF-Linker-F1	InF-Linker-R1<br>InF-pAdeA_SpeI-R	pTAex3-mfmG<br>pTAex3-mfmH	pAdeA digested with *Xba*I
pAdeA-mfmG+H+J	*PamyB-mfmJ-TamyB*	InF-pAdeA_SpeI-F	InF-pAdeA_SpeI-R	pTAex3-mfmJ	pAdeA-mfmG+H digested with *Spe*I
pAdeA-mfmG+F	*PamyB-mfmG-TamyB*<br>*PamyB-mfmF-TamyB*	InF-pAdeA_SpeI-F<br>InF-Linker-F1	InF-Linker-R1<br>InF-pAdeA_SpeI-R	pTAex3-mfmG<br>pTAex3-mfmF	pAdeA digested with *Xba*I
pAdeA-mfmG+F+J	*PamyB-mfmJ-TamyB*	InF-pAdeA_SpeI-F	InF-pAdeA_SpeI-R	pTAex3-mfmJ	pAdeA-mfmG+F digested with *Spe*I
pAdeA-mfmF+H	*PamyB-mfmF-TamyB*<br>*PamyB-mfmH-TamyB*	InF-pAdeA_SpeI-F<br>InF-Linker-F1	InF-Linker-R1<br>InF-pAdeA_SpeI-R	pTAex3-mfmF<br>pTAex3-mfmH	pAdeA digested with *Xba*I
pAdeA-mfmF+H+J	*PamyB-mfmJ-TamyB*	InF-pAdeA_SpeI-F	InF-pAdeA_SpeI-R	pTAex3-mfmJ	pAdeA-mfmF+H digested with *Spe*I
pAdeA-mfmD+H	*PamyB-mfmD-TamyB*<br>*PamyB-mfmH-TamyB*	InF-pAdeA_XbaI-F<br>InF-Linker-F1	InF-Linker-R1<br>InF-pAdeA_XbaI-R	pTAex3-mfmD<br>pTAex3-mfmH	pAdeA digested with *Xba*I
pAdeA-mfmD+ocdTC	*PamyB-mfmD-TamyB*<br>*PamyB-ocdTC-TamyB*	InF-pAdeA_XbaI-F<br>InF-Linker-F1	InF-Linker-R1<br>InF-pAdeA_XbaI-R	pTAex3-mfmD<br>pTAex3-ocdTC	pAdeA digested with *Xba*I
pAdeA-HR-mfmD	*PamyB-mfmD-TamyB*	InF-pAdeA_XbaI-F	InF-pAdeA_XbaI-R	pTAex3-mfmD	pAdeA-HR digested with *Xba*I
pAdeA-HR-psetPT	*PamyB-psetPT-TamyB*	InF-pAdeA_XbaI-F	InF-pAdeA_XbaI-R	pTAex3-psetPT	pAdeA-HR digested with *Xba*I
pTAex3-mfmD (G257Y)	1st fragment of *mfmD* (G257Y)<br>2nd fragment of *mfmD* (G257Y)	mfmD-F<br>mfmD-G257Y-F	mfmD-G257Y-R<br>mfmD-R	pTAex3-mfmD	pTAex3 digested with *Sma*I
pTAex3-mfmD (Y357C)	1st fragment of *mfmD* (Y357C)<br>2nd fragment of *mfmD* (Y357C)	mfmD-F<br>mfmD-Y357C-F	mfmD-Y357C-R<br>mfmD-R	pTAex3-mfmD	pTAex3 digested with *Sma*I
pTAex3-mfmD (G257Y/Y357C)	1st fragment of *mfmD* (G257Y/Y357C)<br>2nd fragment of *mfmD* (G257Y/Y357C)<br>3rd fragment of *mfmD* (G257Y/Y357C)	mfmD-F<br>mfmD-G257Y-F<br>mfmD-Y357C-F	mfmD-G257Y-R<br>mfmD-Y357C-R<br>mfmD-R	pTAex3-mfmD	pTAex3 digested with *Sma*I
pTAex3-psetPT (Y251G)	1st fragment of *psetPT* (Y251G)<br>2nd fragment of *psetPT* (Y251G)	psetPT-F<br>psetPT-Y251G-F	psetPT-Y251G-R<br>psetPT-R	pTAex3-psetPT	pTAex3 digested with *Sma*I
pTAex3-psetPT (C348Y)	1st fragment of *psetPT* (C348Y)<br>2nd fragment of *psetPT* (C348Y)	psetPT-F<br>psetPT-C348Y-F	psetPT-C348Y-R<br>psetPT-R	pTAex3-psetPT	pTAex3 digested with *Sma*I
pTAex3-psetPT (Y251G/C348Y)	1st fragment of *psetPT* (Y251G/C348Y)<br>2nd fragment of *psetPT* (Y251G/C348Y)<br>3rd fragment of *psetPT* (Y251G/C348Y)	psetPT-F<br>psetPT-Y251G-F<br>psetPT-C348Y-F	psetPT-Y251G-R<br>psetPT-C348Y-R<br>psetPT-R	pTAex3-psetPT	pTAex3 digested with *Sma*I
pAdeA-HR-mfmD (G257Y)	*PamyB-mfmD* (G257Y)-*TamyB*	InF-pAdeA_XbaI-F	InF-pAdeA_XbaI-R	pTAex3-mfmD (G257Y)	pAdeA-HR digested with *Xba*I
pAdeA-HR-mfmD (Y357C)	*PamyB-mfmD* (Y357C)-*TamyB*	InF-pAdeA_XbaI-F	InF-pAdeA_XbaI-R	pTAex3-mfmD (Y357C)	pAdeA-HR digested with *Xba*I
pAdeA-HR-mfmD (G257Y/Y357C)	*PamyB-mfmD* (G257Y/Y357C)-*TamyB*	InF-pAdeA_XbaI-F	InF-pAdeA_XbaI-R	pTAex3-mfmD (G257Y+Y357C)	pAdeA-HR digested with *Xba*I
pAdeA-HR-psetPT (Y251G)	*PamyB*-psetPT (Y251G)-*TamyB*	InF-pAdeA_XbaI-F	InF-pAdeA_XbaI-R	pTAex3-psetPT (Y251G)	pAdeA-HR digested with *Xba*I
pAdeA-HR-psetPT (C348Y)	*PamyB*-psetPT (C348Y)-*TamyB*	InF-pAdeA_XbaI-F	InF-pAdeA_XbaI-R	pTAex3-psetPT (C348Y)	pAdeA-HR digested with *Xba*I
pAdeA-HR-psetPT (Y251G/C348Y)	*PamyB*-psetPT (Y251G/C348Y)-*TamyB*	InF-pAdeA_XbaI-F	InF-pAdeA_XbaI-R	pTAex3-psetPT (Y251G+C348Y)	pAdeA-HR digested with *Xba*I

Table S6. *Aspergillus oryzae* transformants constructed in this study.

**Strain**	**Host strain**	**Plasmids used for transformation**
A. oryzae/mfmB	*A. oryzae* NSARU1	pTAex3-mfmB
A. oryzae/mfmBAC	*A. oryzae/mfmB*	pPyrG-mfmA+C
A. oryzae/mfmBACE	*A. oryzae/mfmB*	pPyrG-mfmA+C+E
A. oryzae/mfmBACEF	*A. oryzae/mfmB*	pPyrG-mfmA+C+E+F
A. oryzae/mfmBACDE	*A. oryzae/mfmB*	pPyrG-mfmA+C+D+E
A. oryzae/mfmBACDEFGHJ	*A. oryzae/mfmBACDE*	pAdeA-mfmF+G+H+J
A. oryzae/mfmBACEFGHJ	*A. oryzae/mfmB*	pPyrG-mfmA+C+E, pAdeA-mfmF+G+H+J
A. oryzae/mfmBACDFGHJ	*A. oryzae/mfmB*	pPyrG-mfmA+C+D, pAdeA-mfmF+G+H+J
A. oryzae/mfmBACDEGHJ	*A. oryzae/mfmBACDE*	pAdeA-mfmG+H+J
A. oryzae/mfmBACDEFHJ	*A. oryzae/mfmBACDE*	pAdeA-mfmF+H+J
A. oryzae/mfmBACDEFGJ	*A. oryzae/mfmBACDE*	pAdeA-mfmF+G+J
A. oryzae/mfmBACDEFGH	*A. oryzae/mfmBACDE*	pAdeA-mfmF+G+H
A. oryzae/mfmBACEFDH	*A. oryzae/mfmBACEF*	pAdeA-mfmD+H
A. oryzae/mfmBACEFD+ocdTC	*A. oryzae/mfmBACEF*	pAdeA-mfmD+ocdTC
A. oryzae/mfmBACEFD	*A. oryzae/mfmBACEF*	pAdeA-HR-mfmD
A. oryzae/mfmBACEF+psetPT	*A. oryzae/mfmBACEF*	pAdeA-HR-psetPT
A. oryzae/mfmBACEFD (G257Y)	*A. oryzae/mfmBACEF*	pAdeA-HR-mfmD (G257Y)
A. oryzae/mfmBACEFD (Y357C)	*A. oryzae/mfmBACEF*	pAdeA-HR-mfmD (Y357C)
A. oryzae/mfmBACEFD (G257Y+Y357C)	*A. oryzae/mfmBACEF*	pAdeA-HR-mfmD (G257Y+Y357C)
A. oryzae/mfmBACEF+psetPT (Y251G)	*A. oryzae/mfmBACEF*	pAdeA-HR-psetPT (Y251G)
A. oryzae/mfmBACEF+psetPT (C348Y)	*A. oryzae/mfmBACEF*	pAdeA-HR-psetPT (C348Y)
A. oryzae/mfmBACEF+psetPT (Y251G+C348Y)	*A. oryzae/mfmBACEF*	pAdeA-HR-psetPT (Y251G+C348Y)

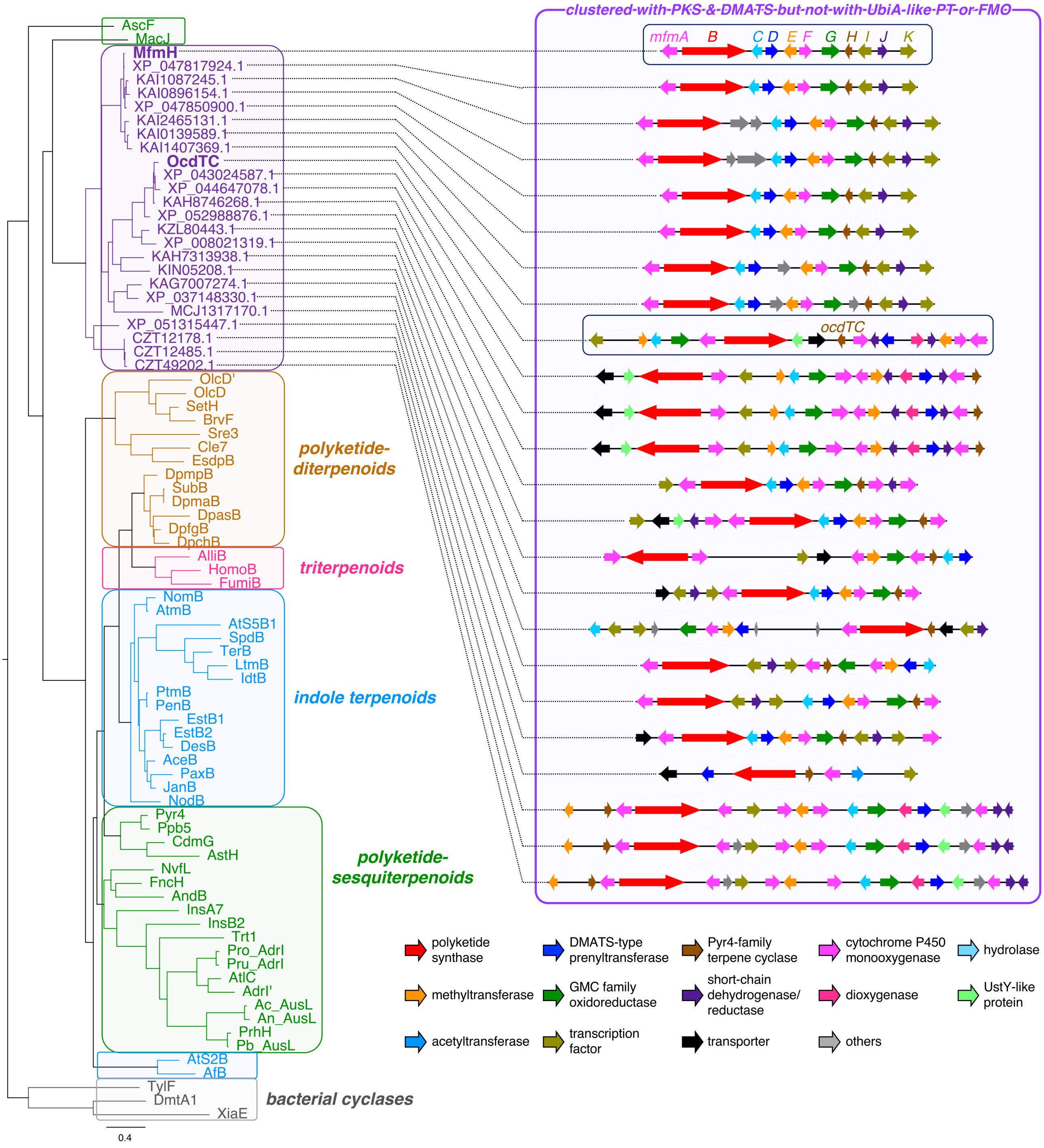

Figure S1. Phylogenetic analysis of the characterized Pyr4 homologues and those clustered with a PKS and a dimethylallyltryptophan synthase (DMATS)-type prenyltransferase identified in this study, along with their associated BGCs. Some of the gene sequences were manually revised upon creating the phylogenetic tree (refer to Table S1 and Supplementary Data for their sequences). The BGCs focused on in this study are boxed.

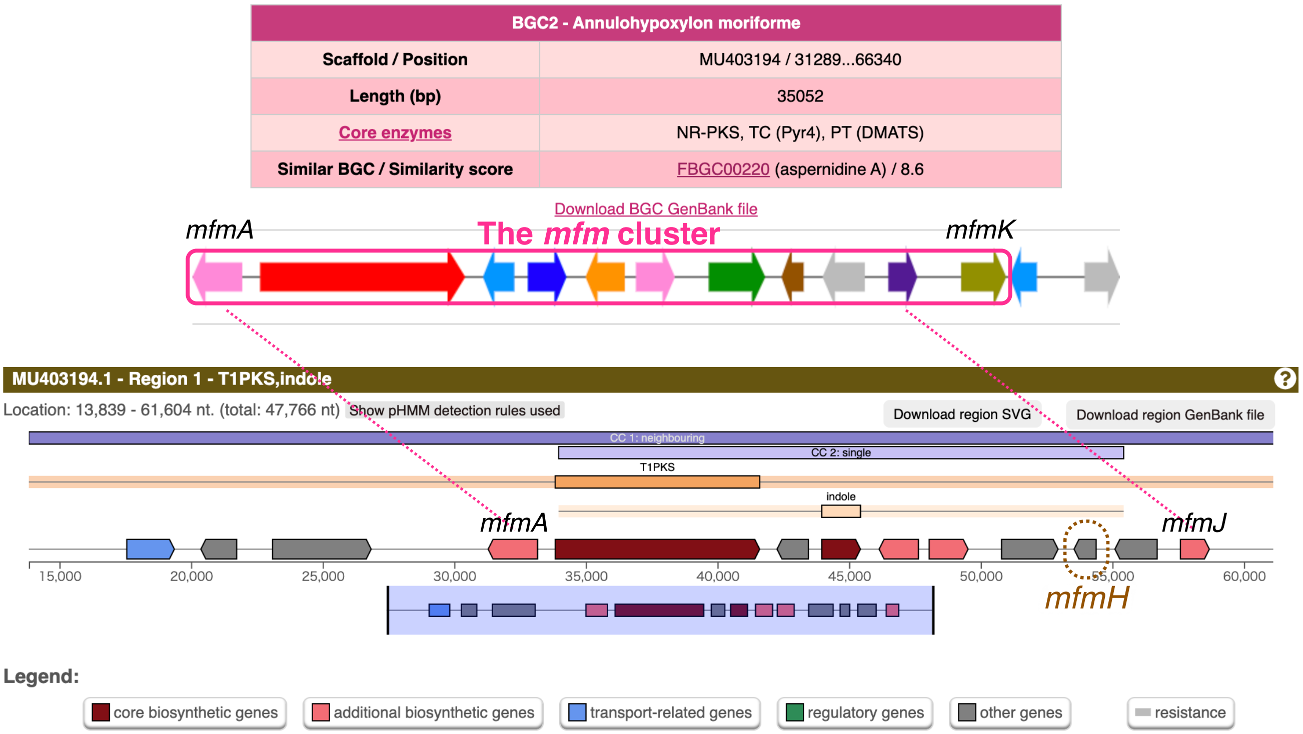

Figure S2. Comparison of BGC extractions by FunBGCeX (top) and antiSMASH (bottom). Genomic regions corresponding to the *mfm* cluster is shown. In the antiSMASH analysis, the terpene cyclase gene *mfmH* is classified as “other genes.”

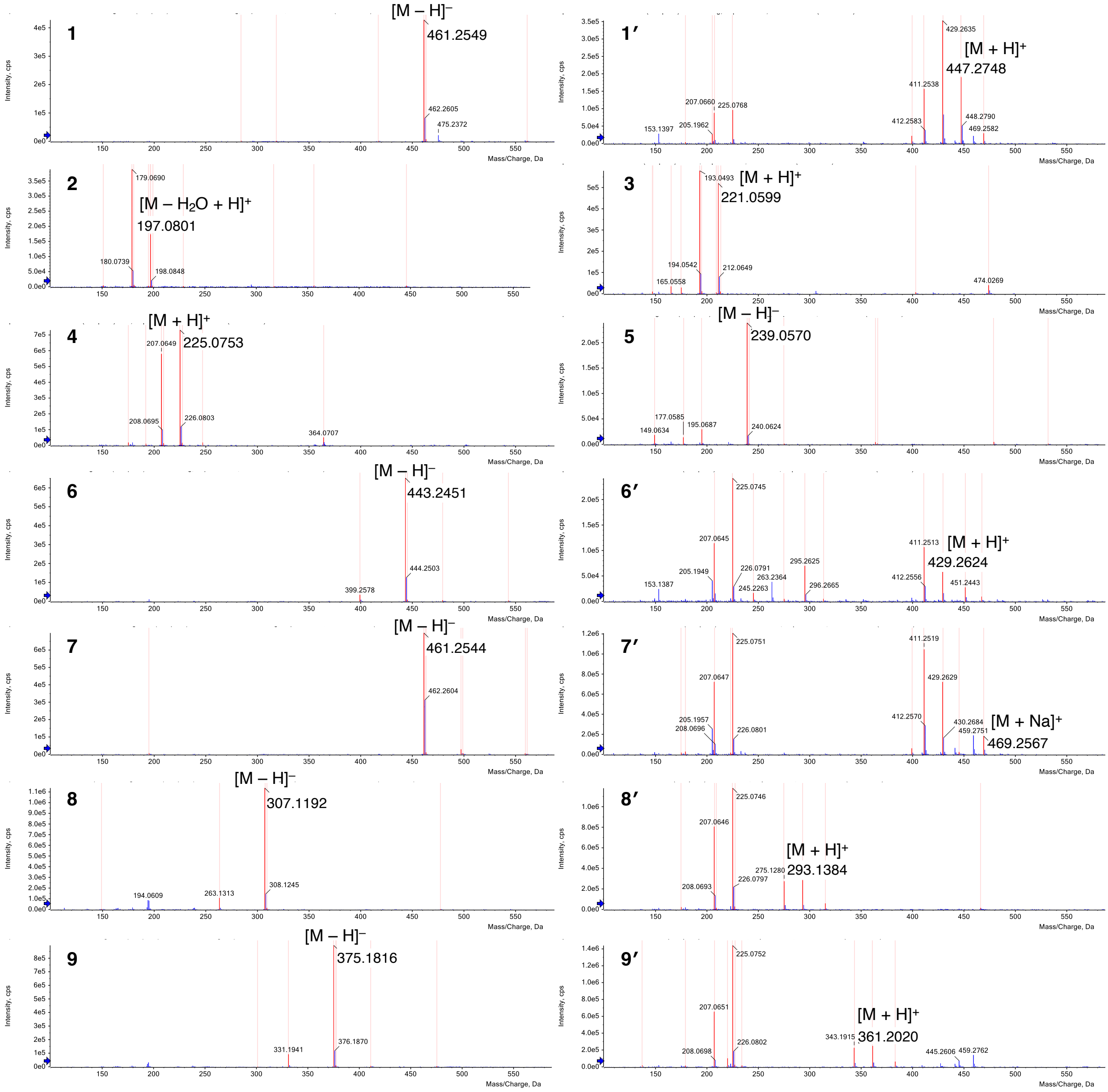

Figure S3. MS spectra of metabolites detected or isolated in this study.

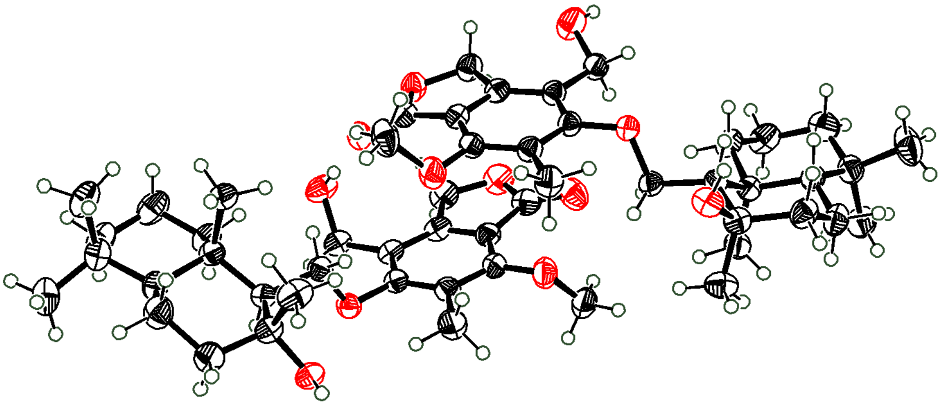

Figure S4. X-ray crystal structure of compound **1′** (with 50% probability of thermal ellipsoid).

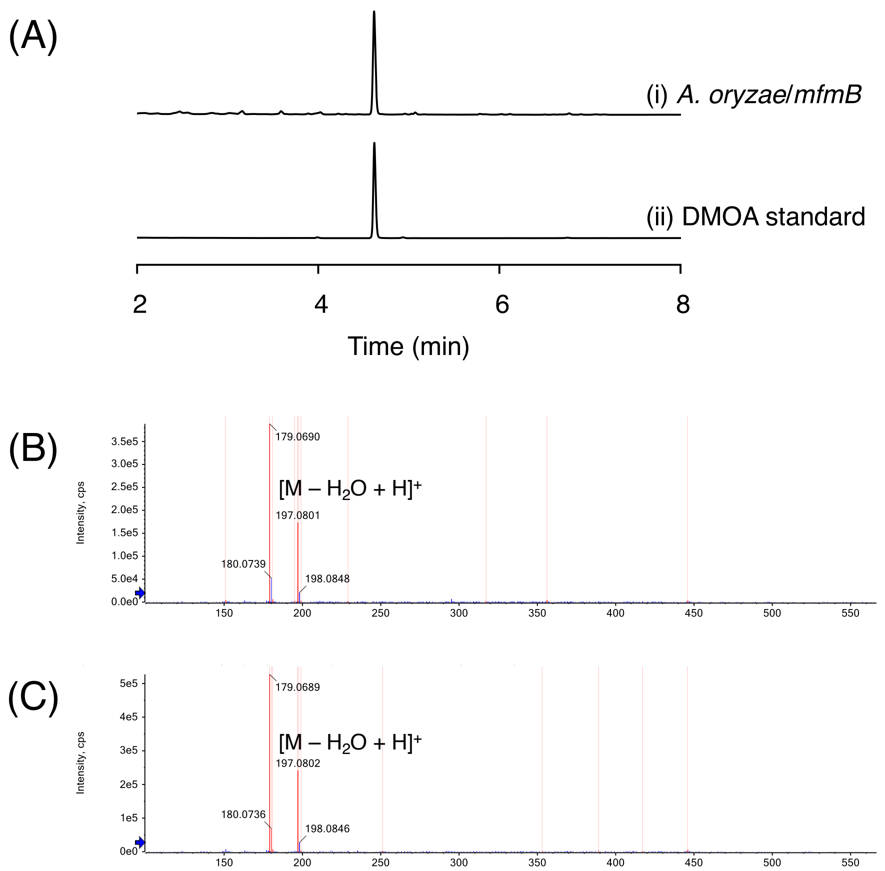

Figure S5. (A) HPLC analysis of (i) the metabolites from the *A. oryzae* transformant expressing *mfmB* and (ii) the DMOA standard. (B, C) MS spectra of (B) **2** detected in the *A. oryzae* transformant and (C) the DMOA standard.

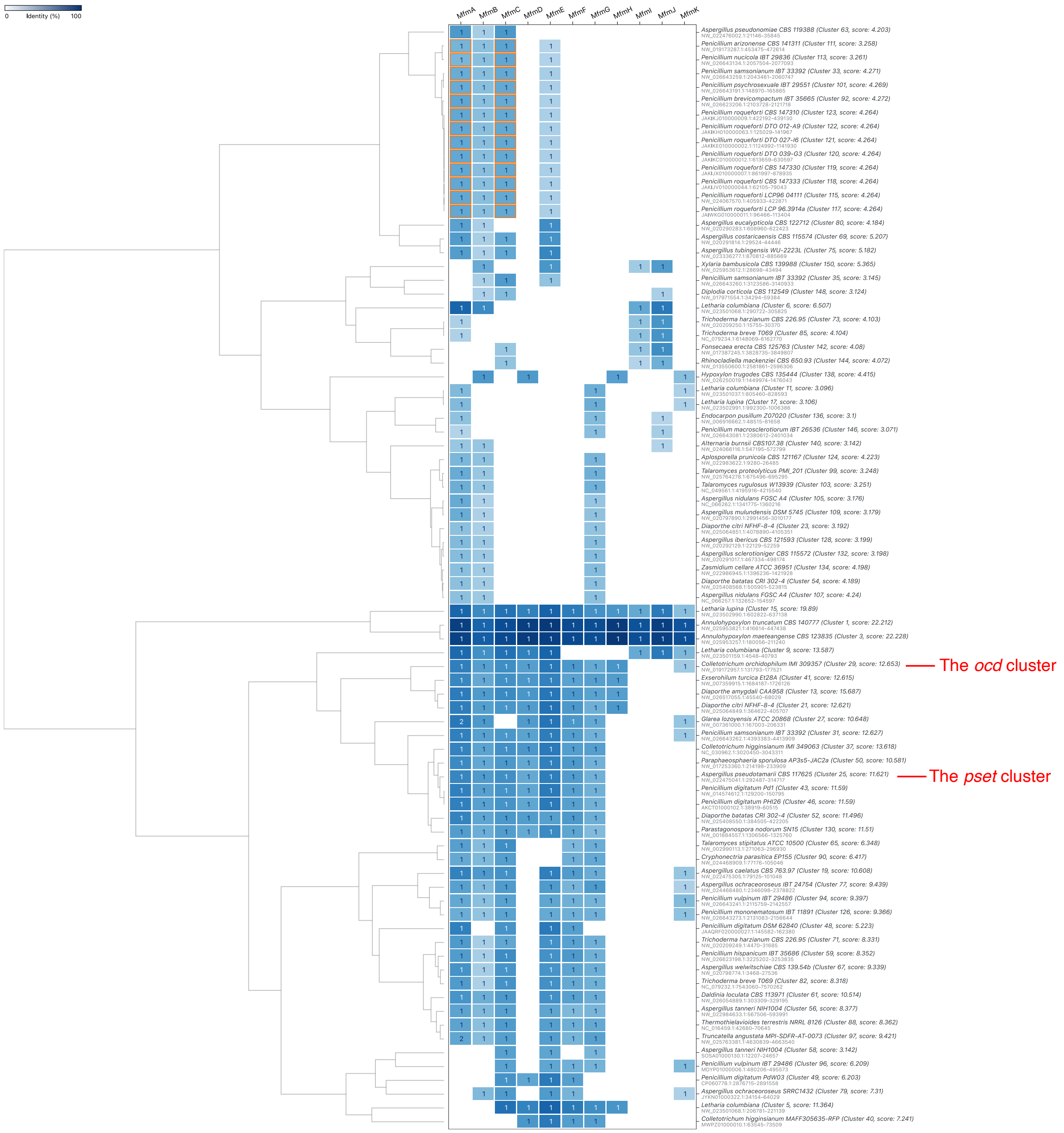

Figure S6. Result of the cblaster analysis against the RefSeq database using the sequences of MfmA–K as queries.

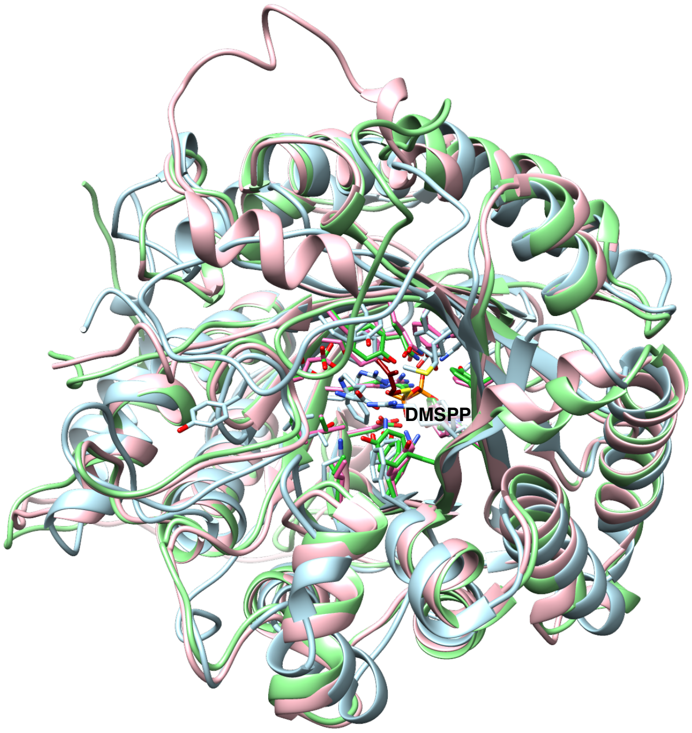

Figure S7. Superimposition of the modeled structures of MfmD (pink) and PsetPT (light green) and the crystal structure of FgaPT2 (light blue) in the complex with DMSPP (PDB: 3I4X).

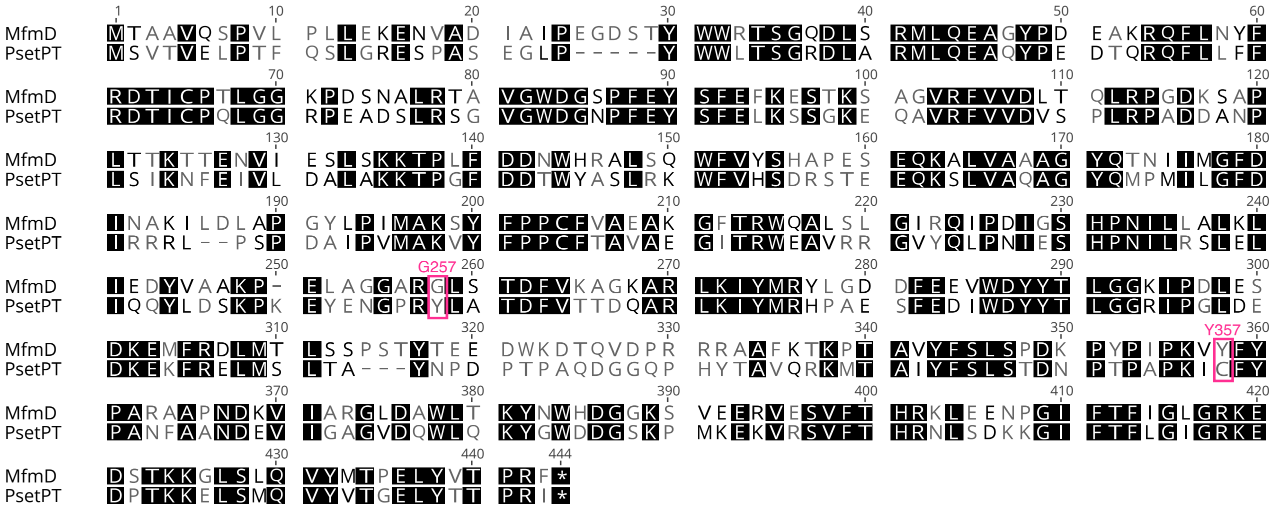

Figure S8. Sequence alignment of MfmD and PsetPT. The amino acid residues important for prenyl donor selectivity are boxed.

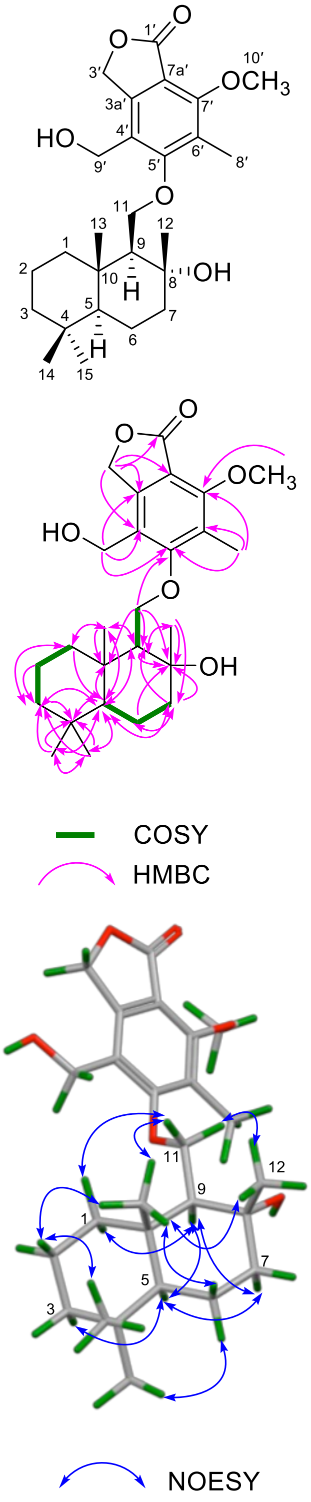

position	δC, type	δH, mult. (*J* in Hz)
1	40.1, CH_2	1.24 (α), m
		1.84 (β), dt (12.8, 3.0)
2	18.4, CH_2	1.50 (α), dquin (14.2, 3.2)
		1.64 (β), m
3	41.7, CH_2	1.21 (α), td (13.8, 4.1)
		1.42 (β), dt (13.1, 3.5)
4	33.2, C	
5	55.7, CH	1.05, dd (12.2, 2.0)
6	20.2, CH_2	1.72 (α), m
		1.29 (β), m
7	43.9, CH_2	1.59 (α), td (13.1, 3.7)
		1.91 (β), dt (12.5, 3.2)
8	73.4, C	
9	61.0, CH	1.88, t (4.7)
10	37.9, C	
11	72.6, CH_2	4.11, dd (9.9, 4.4)
		4.01, dd (9.9, 5.1)
12	24.9, CH_3	1.23, s
13	16.2, CH_3	0.85, s
14	21.5, CH_3	0.82, s
15	33.4, CH_3	0.91, s
1′	168.7, C	
3′	68.5, CH_2	5.34, d (15.4)
		5.28, d (15.4)
3a′	146.8, C	
4′	123.6, C	
5′	162.2, C	
6′	125.6, C	
7′	158.2, C	
7a′	112.9, C	
8′	10.0, CH_3	2.25, s
9′	56.9, CH_2	4.81, t (12.7)
		4.51, t (12.7)
10′	62.3, CH_3	4.04, s

^{1}H NMR: 600 MHz, ^{13}C NMR: 150 MHz (in $CDCl_3$)

Figure S9. NMR data of **1′**.

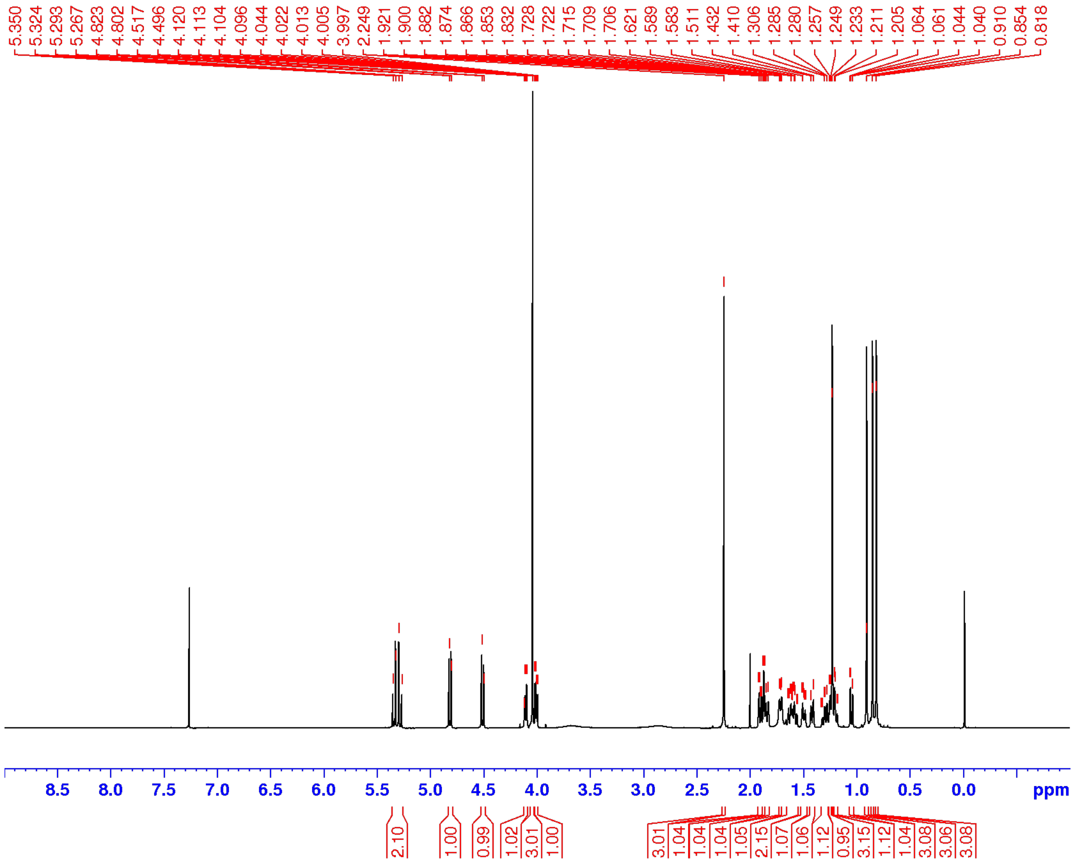

Figure S10. ^{1}H NMR spectrum of **1′** in $CDCl_3$ at 600 MHz.

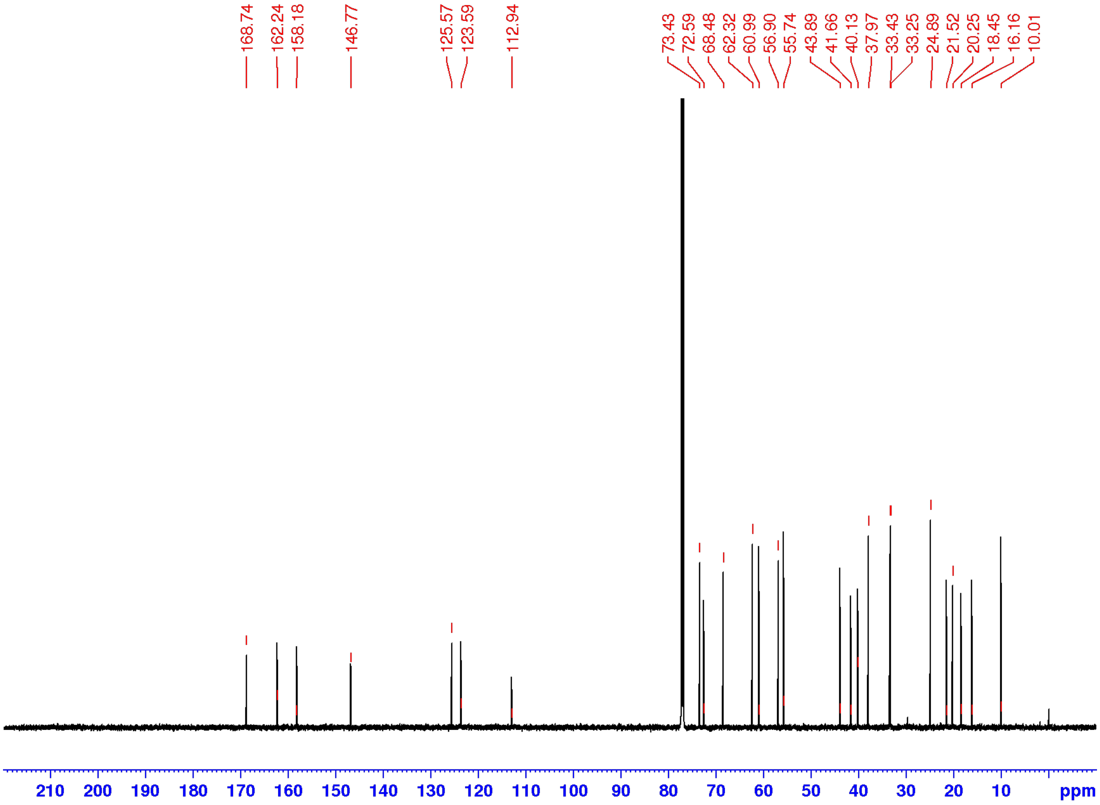

Figure S11. ^{13}C{^{1}H} NMR spectrum of **1′** in $CDCl_3$ at 150 MHz.

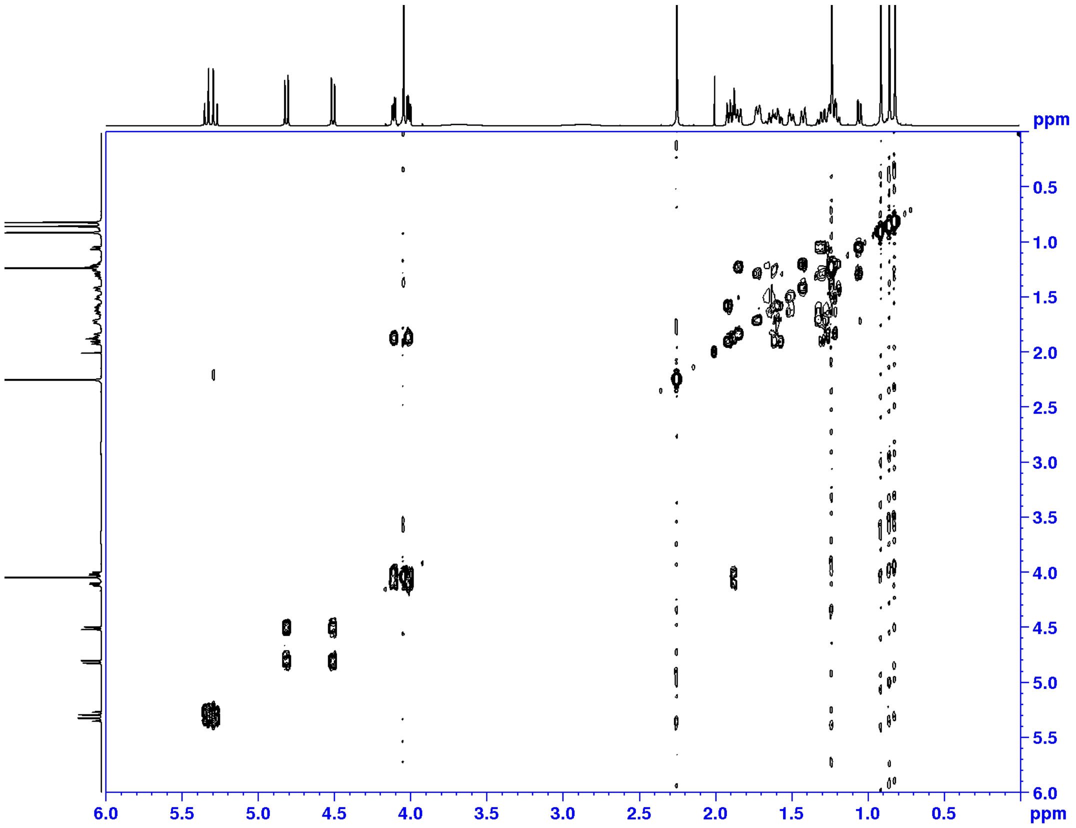

Figure S12. ^{1}H-^{1}H COSY spectrum of **1′** in $CDCl_3$.

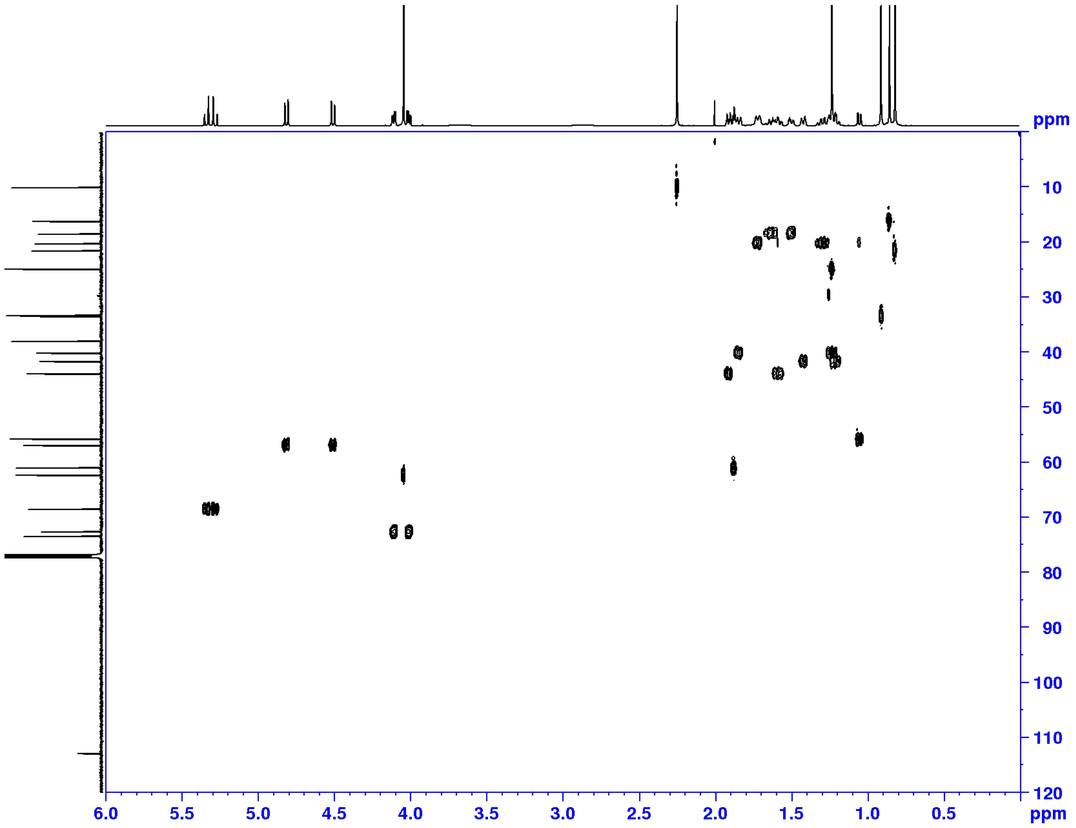

Figure S13. HSQC spectrum of **1′** in $CDCl_3$.

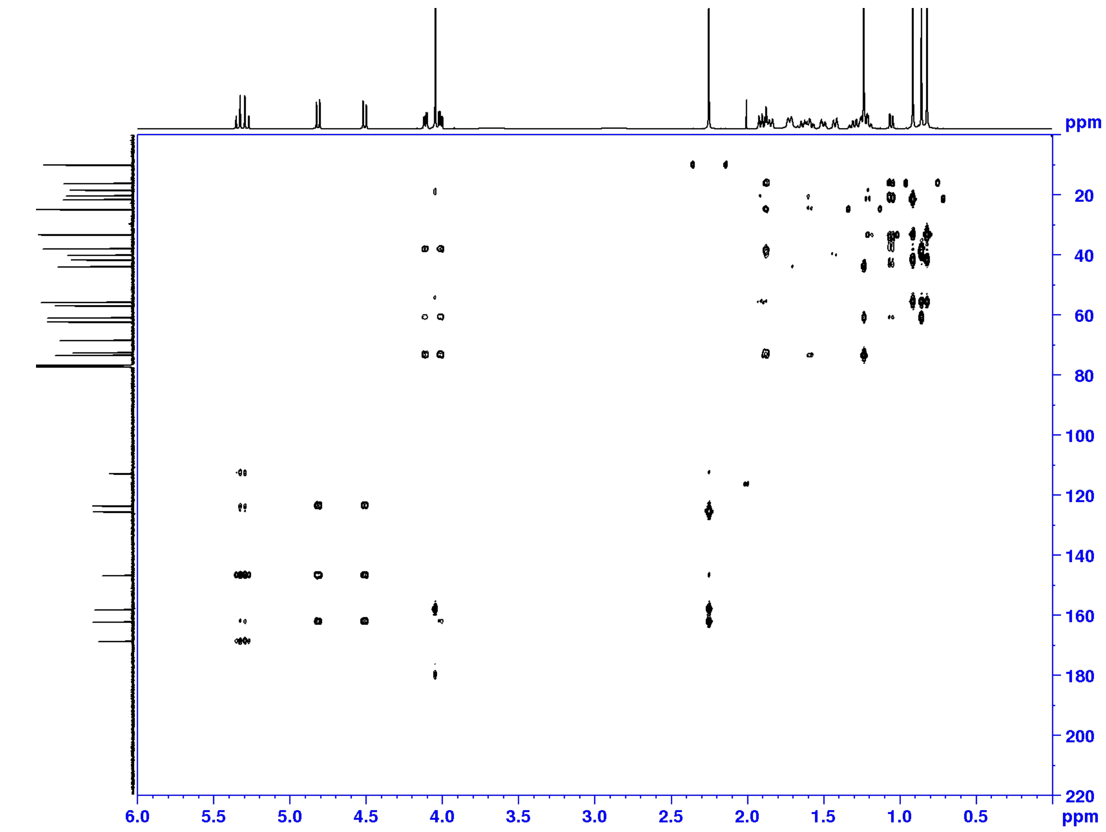

Figure S14. HMBC spectrum of **1′** in $CDCl_3$.

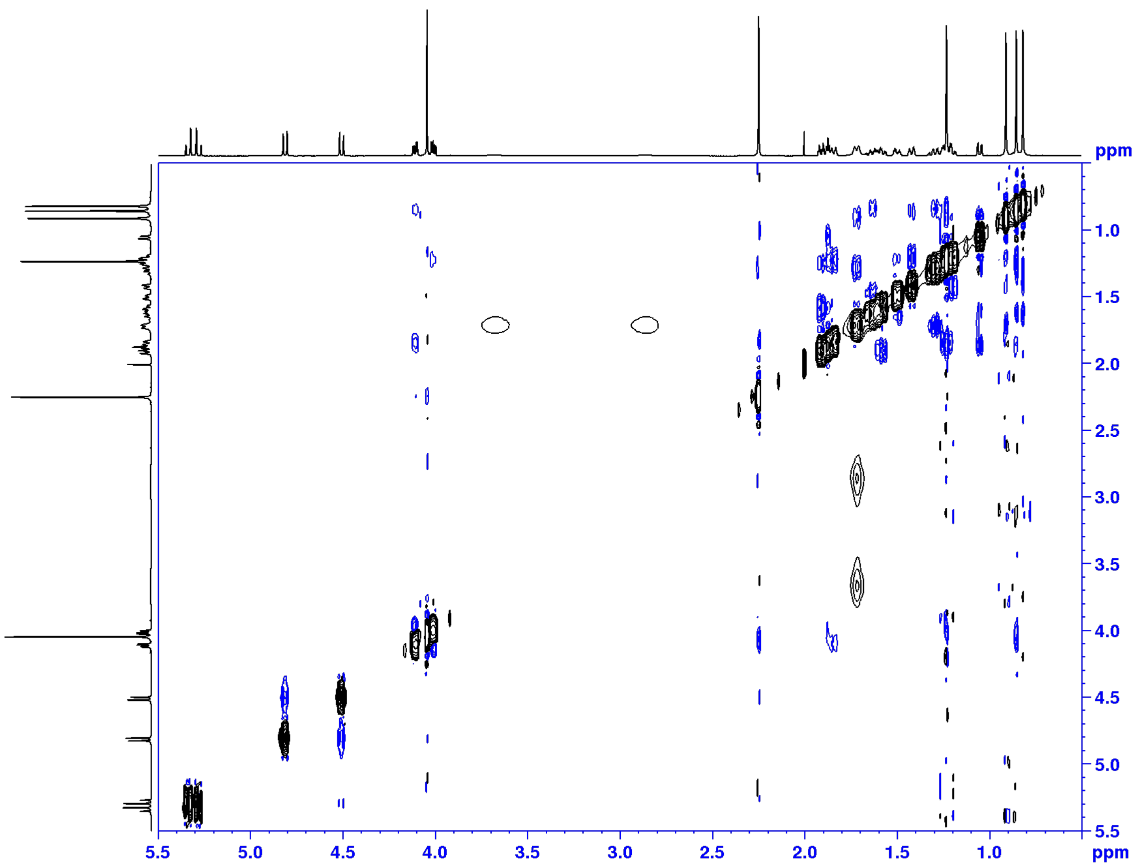

Figure S15. NOESY spectrum of **1′** in $CDCl_3$.

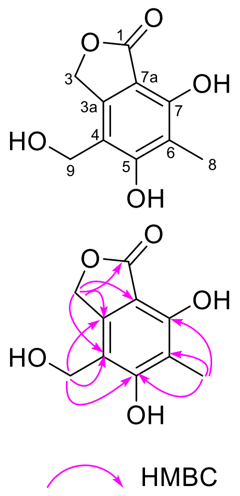

position	δ_C, type	δ_H, mult. (J in Hz)
1	170.4, C	
3	68.9, CH_2	5.29, s
3a	144.4, C	
4	114.1, C	
5	159.5, C	
6	111.6, C	
7	153.6, C	
7a	102.8, C	
8	8.5, CH_3	2.03, s
9	56.8, CH_2	4.56, s
OH		9.11, s
OH		9.53, brs

^{1}H NMR: 600 MHz, ^{13}C NMR: 150 MHz (in DMSO-d_6)

Figure S16. NMR data of **3**.

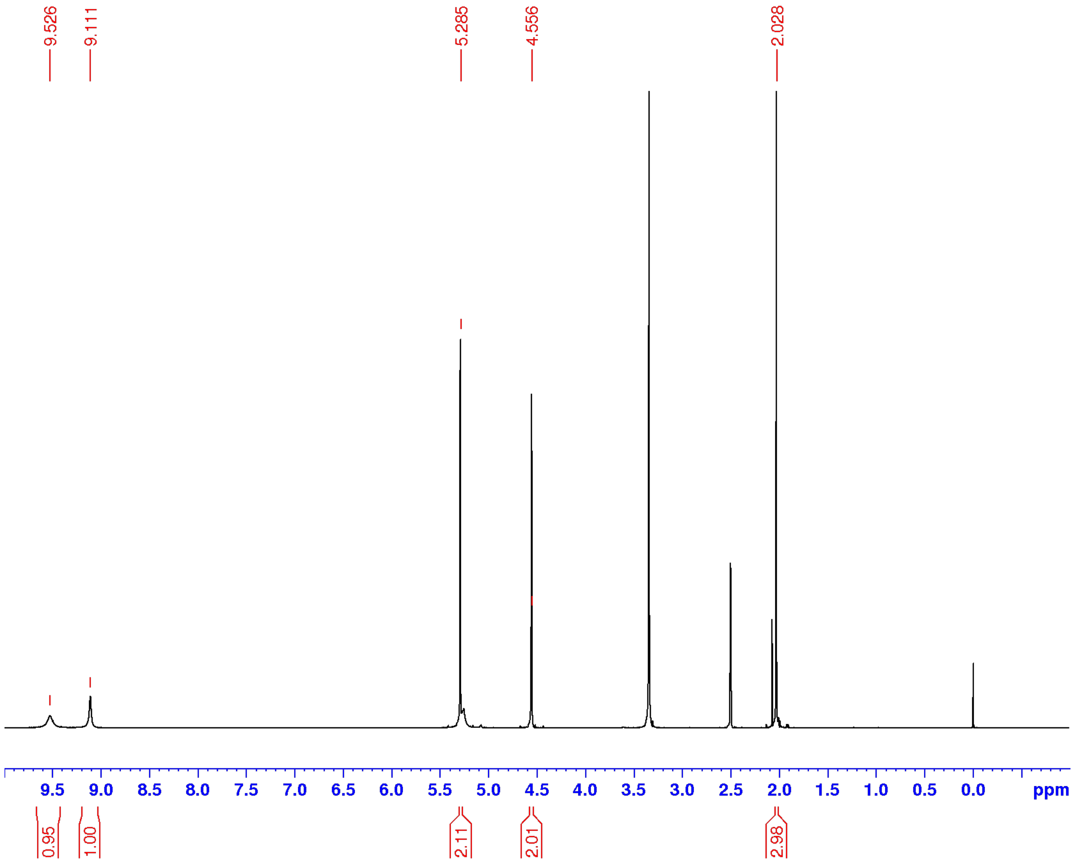

Figure S17. ^{1}H NMR spectrum of **3** in DMSO-d_6 at 600 MHz.

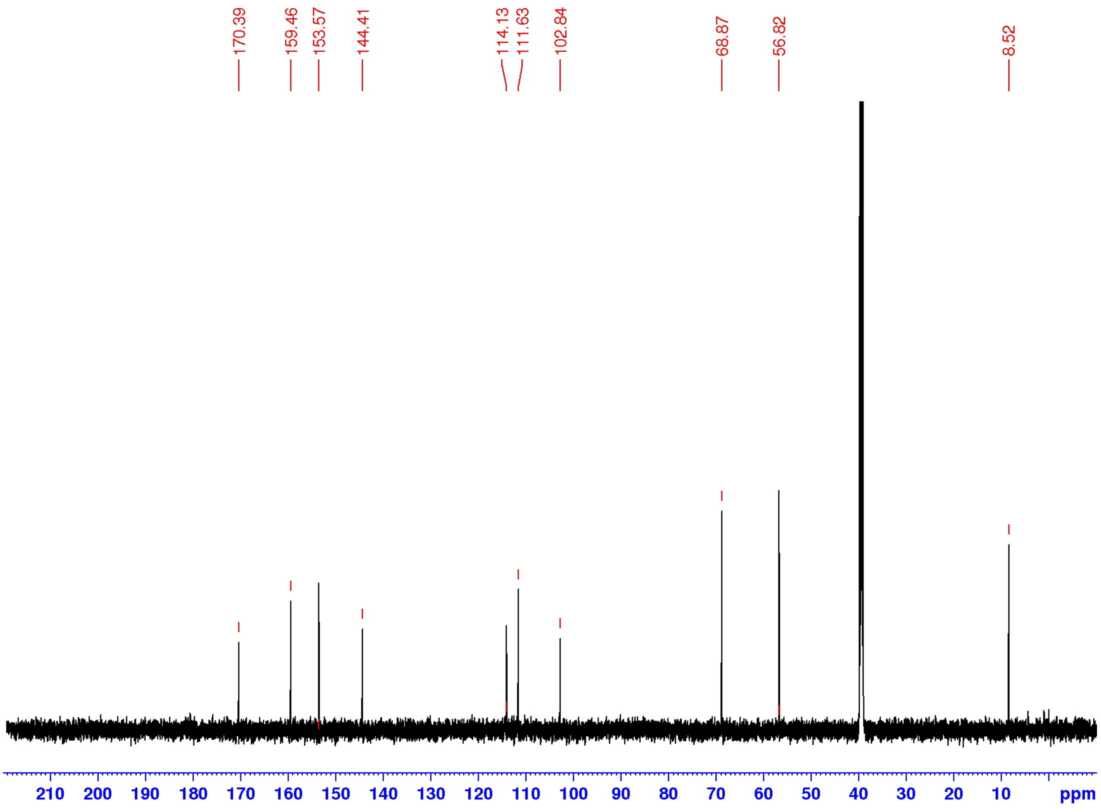

Figure S18. $^{13}C\{^{1}H\}$ NMR spectrum of **3** in DMSO-d_6 at 150 MHz.

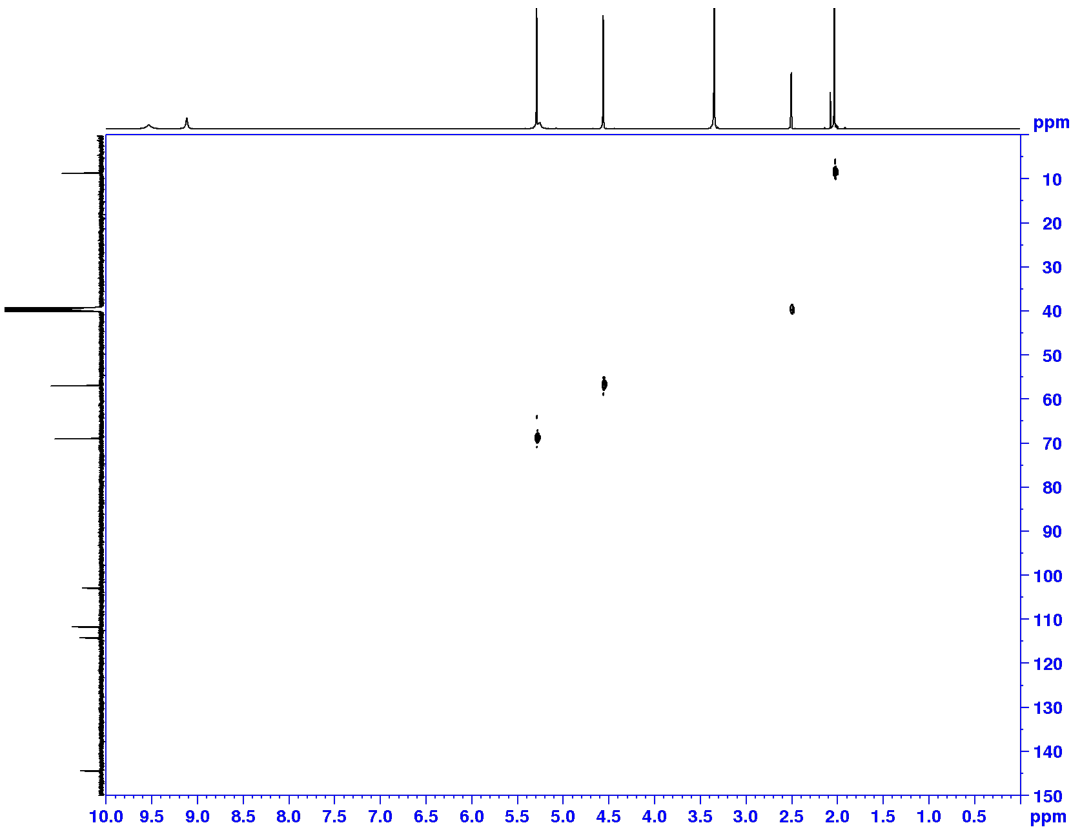

Figure S19. HSQC spectrum of **3** in DMSO-d_6.

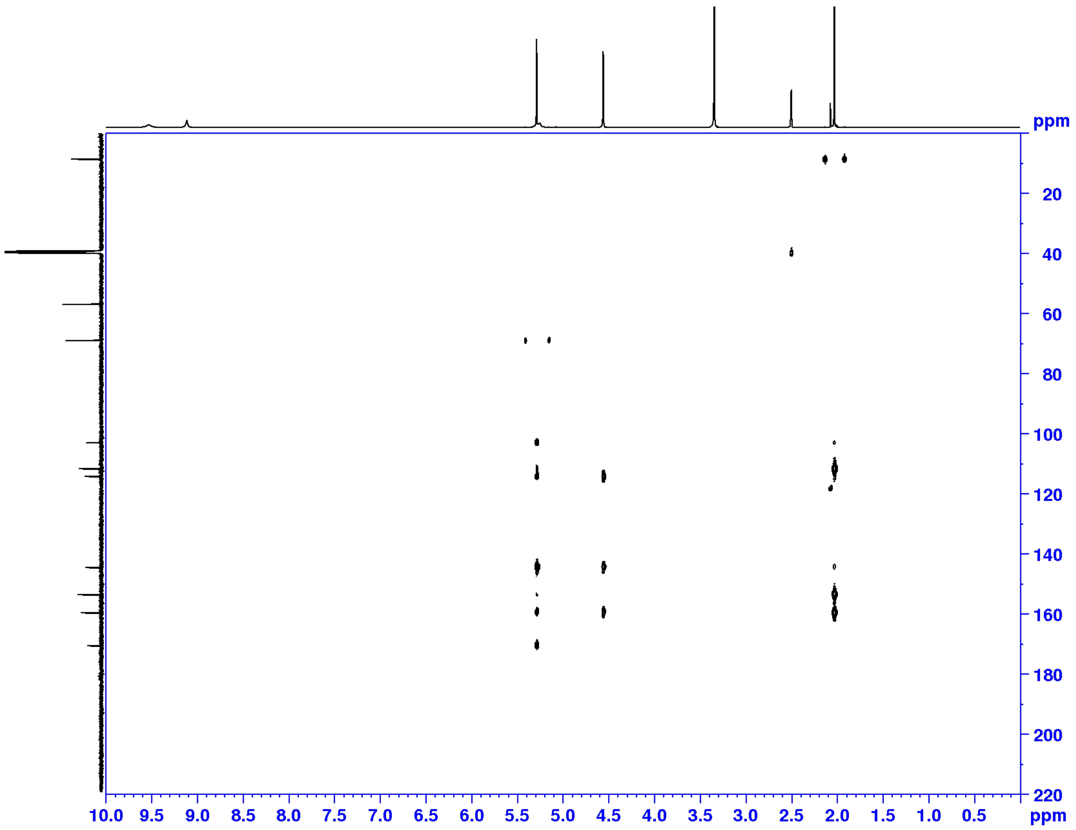

Figure S20. HMBC spectrum of **3** in DMSO-d_6.

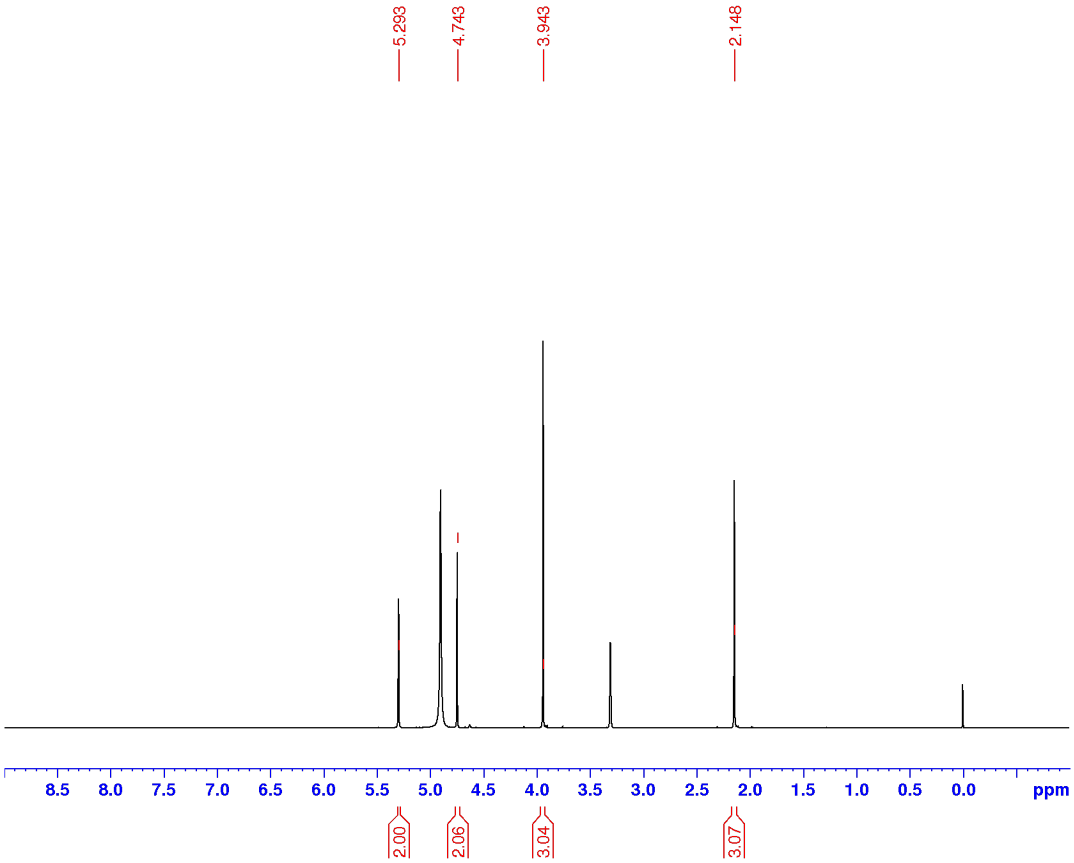

Figure S21. ^{1}H NMR spectrum of **4** in CD_3OD at 400 MHz.

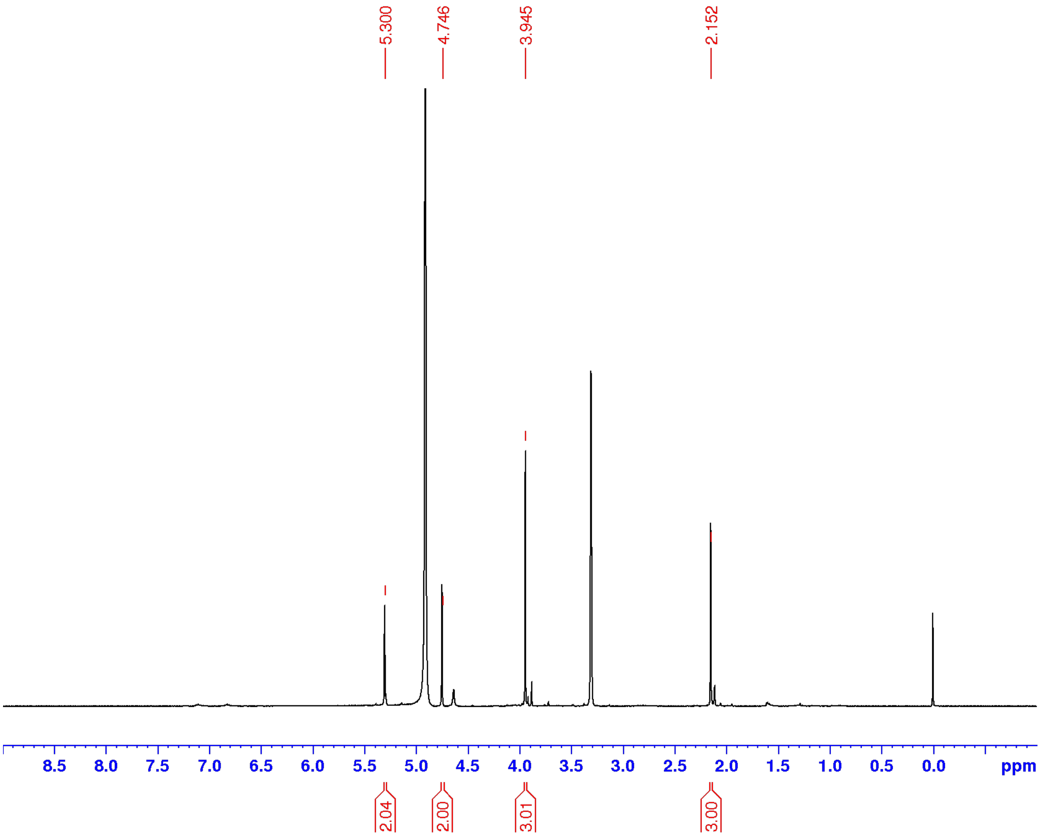

Figure S22. ^{1}H NMR spectrum of **4** (from the reduction of **5**) in CD_3OD at 400 MHz.

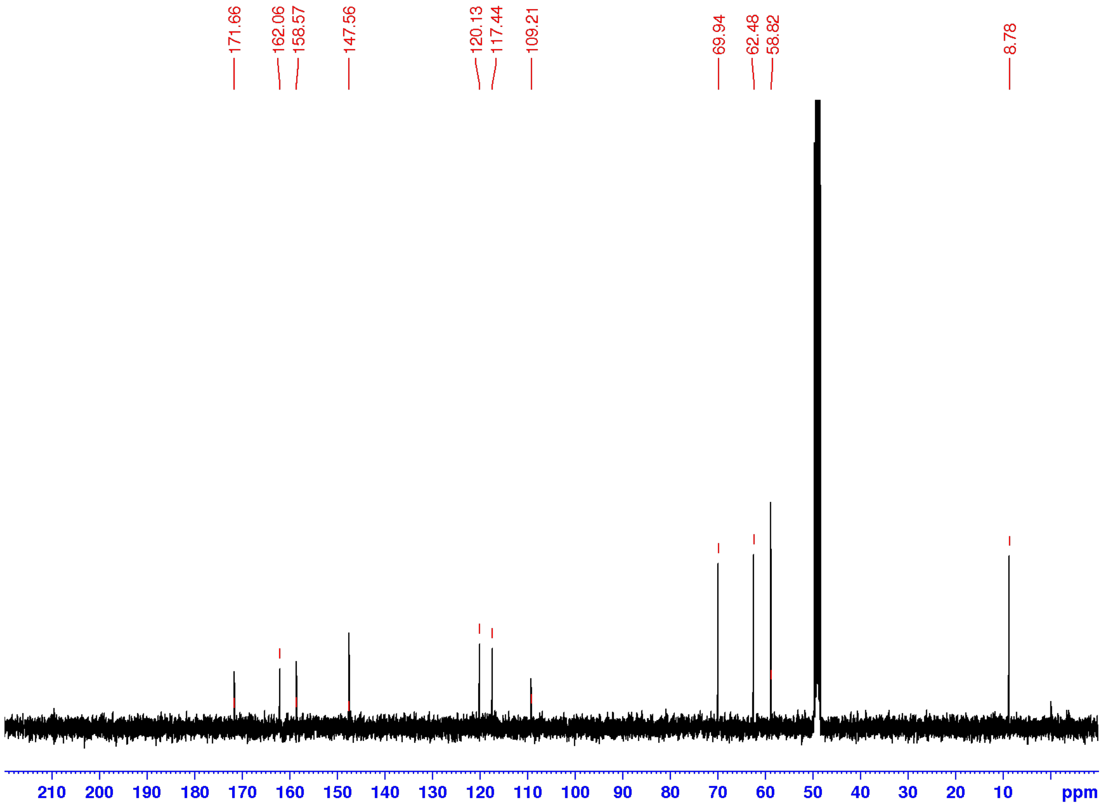

Figure S23. $^{13}C\{^1H\}$ NMR spectrum of **4** in CD_3OD at 100 MHz.

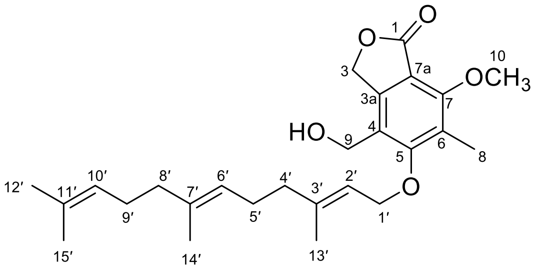

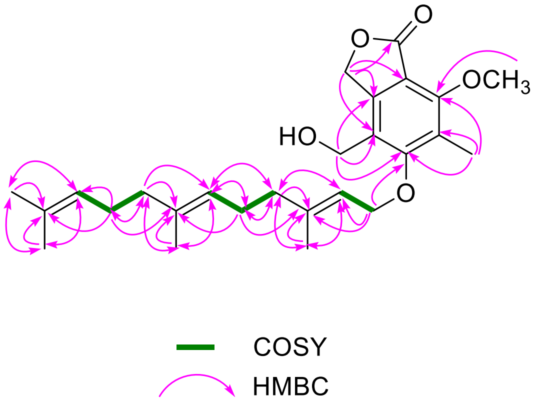

position	δC, type	δH, mult. (*J* in Hz)
1	168.9, C	
3	69.5, CH2	5.40, s
3a	147.9, C	
4	125.9, C	
5	161.8, C	
6	125.9, C	
7	158.1, C	
7a	113.8, C	
8	9.8, CH3	2.23, s
9	57.9, CH2	4.81, s
10	62.2, CH3	4.00, s
1′	71.5, CH2	4.48, d (7.1)
2′	127.0, CH	5.59, tq (7.1, 1.2)
3′	142.2, C	
4′	40.2, CH2	2.10, brt (6.7)
5′	27.0, CH2	2.16, m
6′	124.6, CH	5.16, tq (7.0, 1.1)
7′	136.0, C	
8′	40.4, CH2	1.99, brt (7.5)
9′	27.4, CH2	2.08, m
10′	125.1, CH	5.11, tsept (7.1, 1.2)
11′	131.7, C	
12′	25.8, CH3	1.65, s
13′	16.5, CH3	1.70, s
14′	16.1, CH3	1.62, s
15′	17.7, CH3	1.59, s

^{1}H NMR: 600 MHz, ^{13}C NMR: 150 MHz (in acetone-*d*6)

Figure S24. NMR data of **6′**.

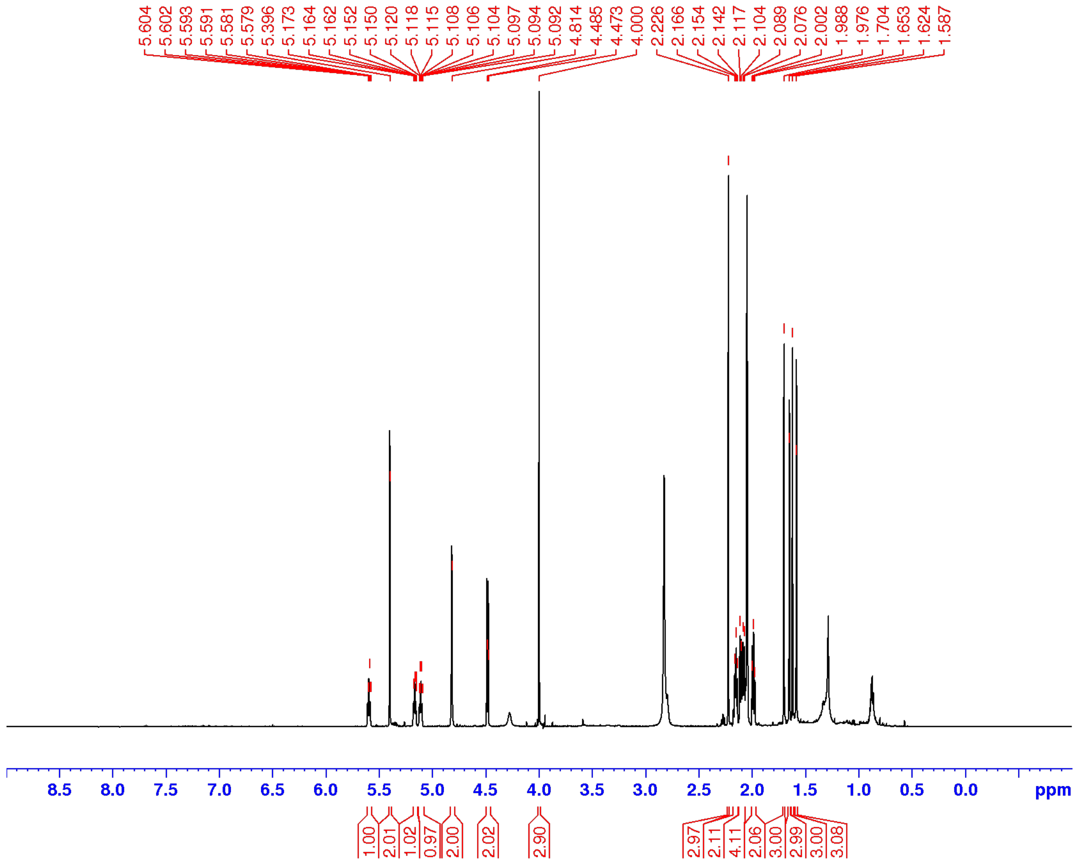

Figure S25. ^{1}H NMR spectrum of **6′** in acetone-d_6 at 600 MHz.

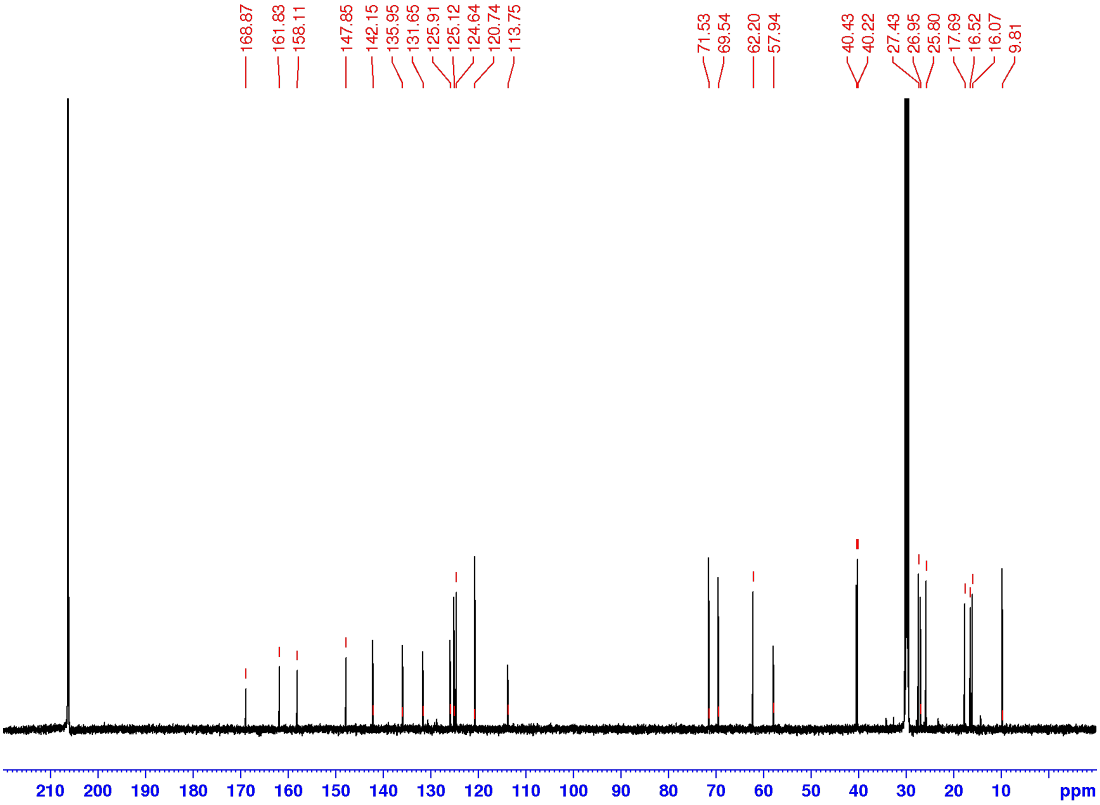

Figure S26. $^{13}C\{^{1}H\}$ NMR spectrum of **6′** in acetone-d_6 at 150 MHz.

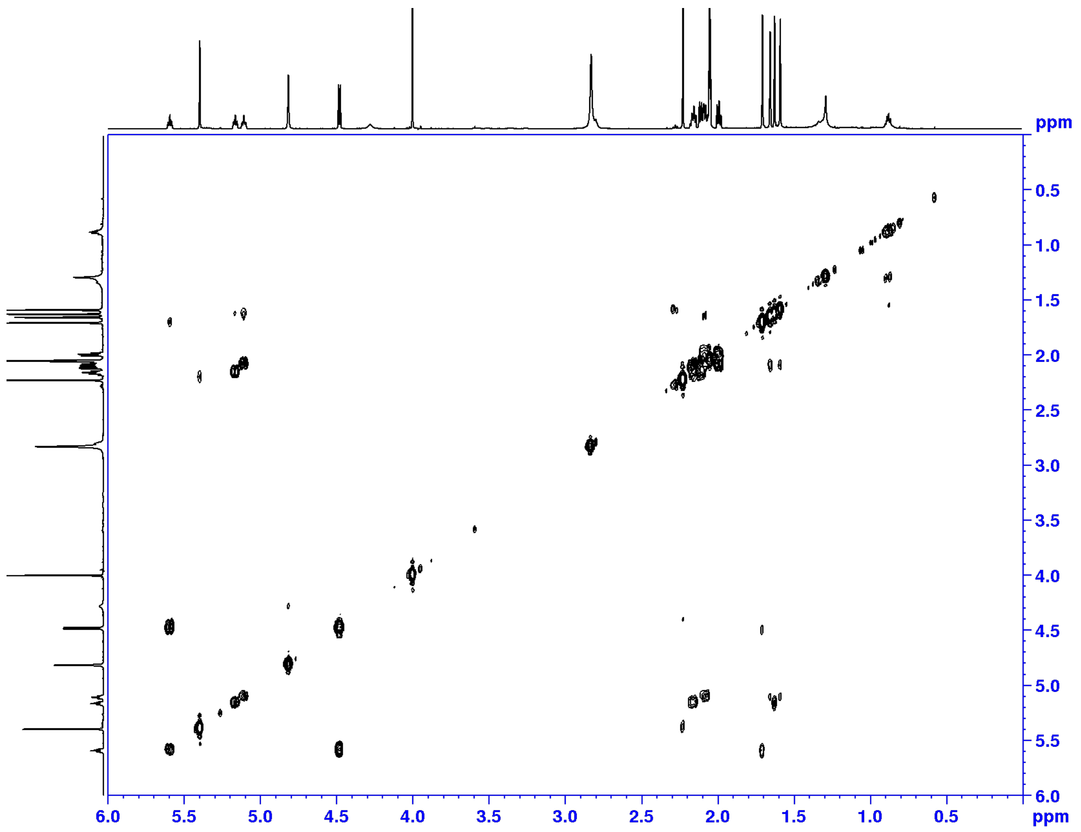

Figure S27. ^{1}H-^{1}H COSY spectrum of **6′** in acetone-d_6.

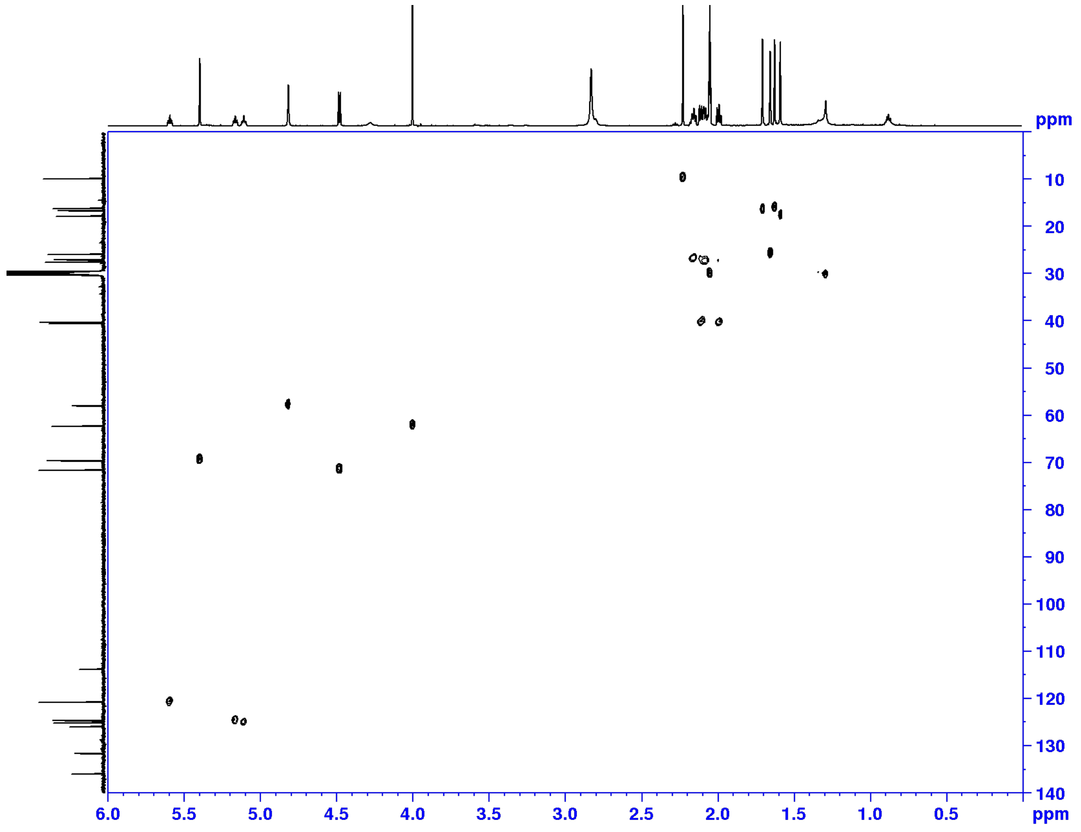

Figure S28. HSQC spectrum of **6′** in acetone-d_6.

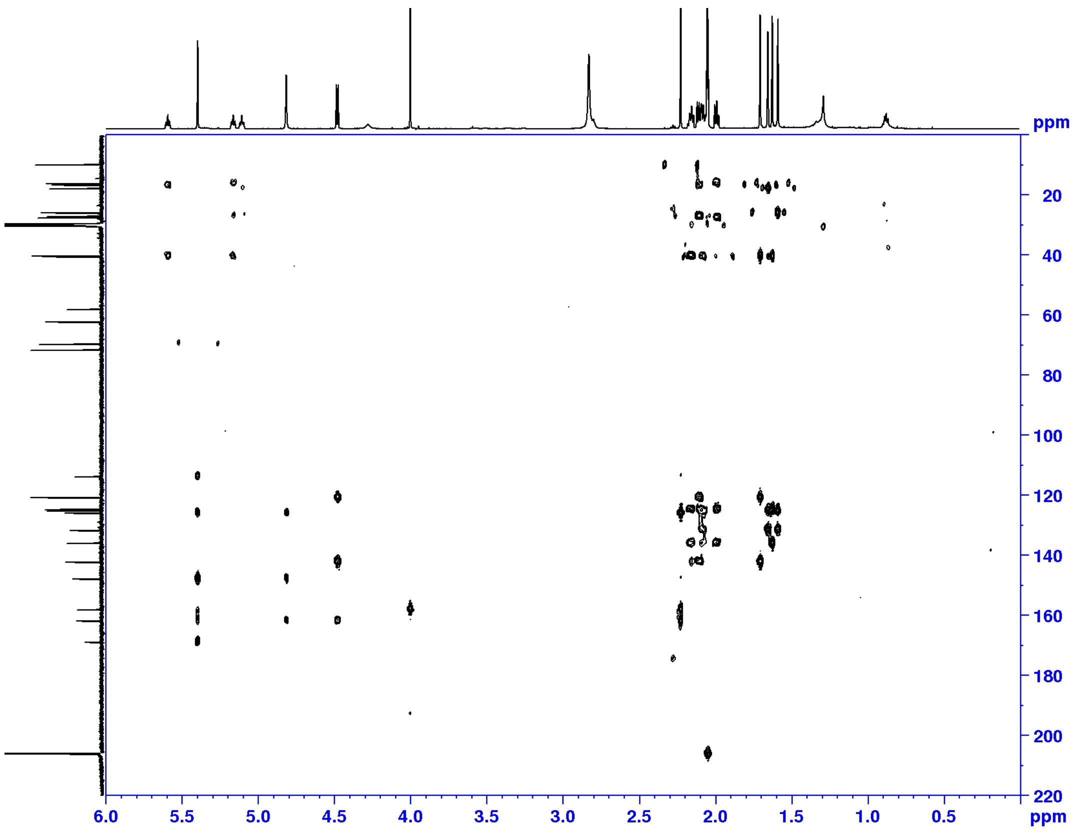

Figure S29. HMBC spectrum of **6′** in acetone-d_6.

.

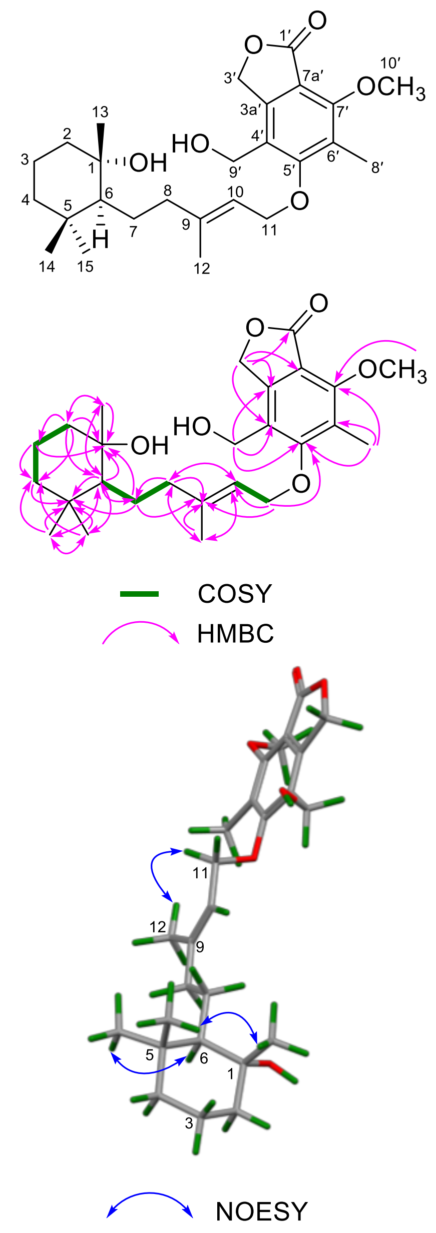

position	δ_C, type	δ_H, mult. (*J* in Hz)
1	73.6, C	
2	44.2, CH_2	1.38 (α), overlapped
		1.69 (β), overlapped
3	21.2, CH_2	1.49, m
4	42.4, CH_2	1.21 (α), td (12.6, 5.3)
		1.36 (β), dt (11.8, 3.2)
5	36.1, C	
6	57.3, CH	1.19 (α), t (4.4)
7	25.5, CH_2	1.69, m
		1.40, m
8	43.5, CH_2	2.34, ddd (13.9, 9.7, 5.5)
		2.13, ddd (13.9, 9.6, 5.3)
9	143.9, C	
10	119.9, CH	5.58, tq (7.1, 1.0)
11	71.5, CH_2	4.48, d (7.1)
12	16.7, CH_3	1.70, brs
13	23.7, CH_3	1.14, s
14	21.8, CH_3	0.84, s
15	33.3, CH_3	0.96, s
1′	168.9, C	
3′	69.6, CH_2	5.40, s
3a′	147.8, C	
4′	126.0, C	
5′	161.8, C	
6′	126.0, C	
7′	158.1, C	
7a′	113.7, C	
8′	9.9, CH_3	2.23, s
9′	58.0, CH_2	4.82, s
10′	62.2, CH_3	4.00, s

^{1}H NMR: 600 MHz, ^{13}C NMR: 150 MHz (in acetone-d_6)

Figure S30. NMR data of **7′**.

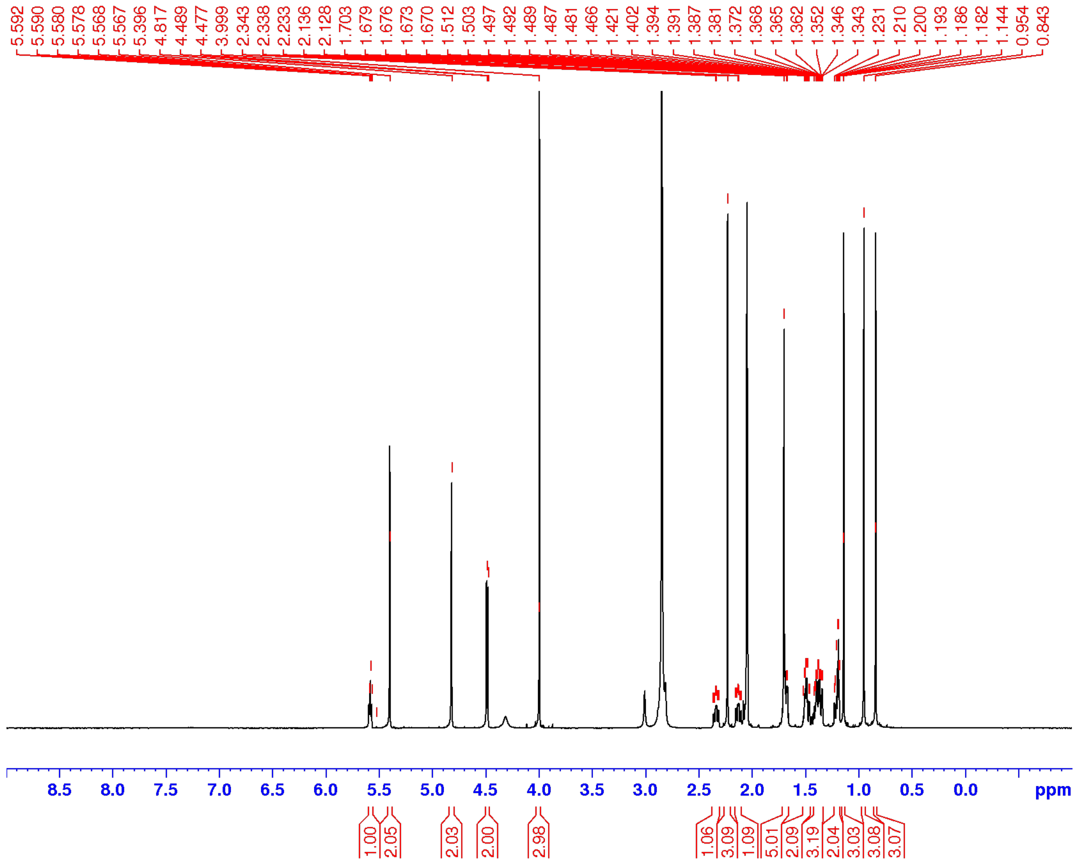

Figure S31. ^{1}H NMR spectrum of **7′** in acetone-d_6 at 600 MHz.

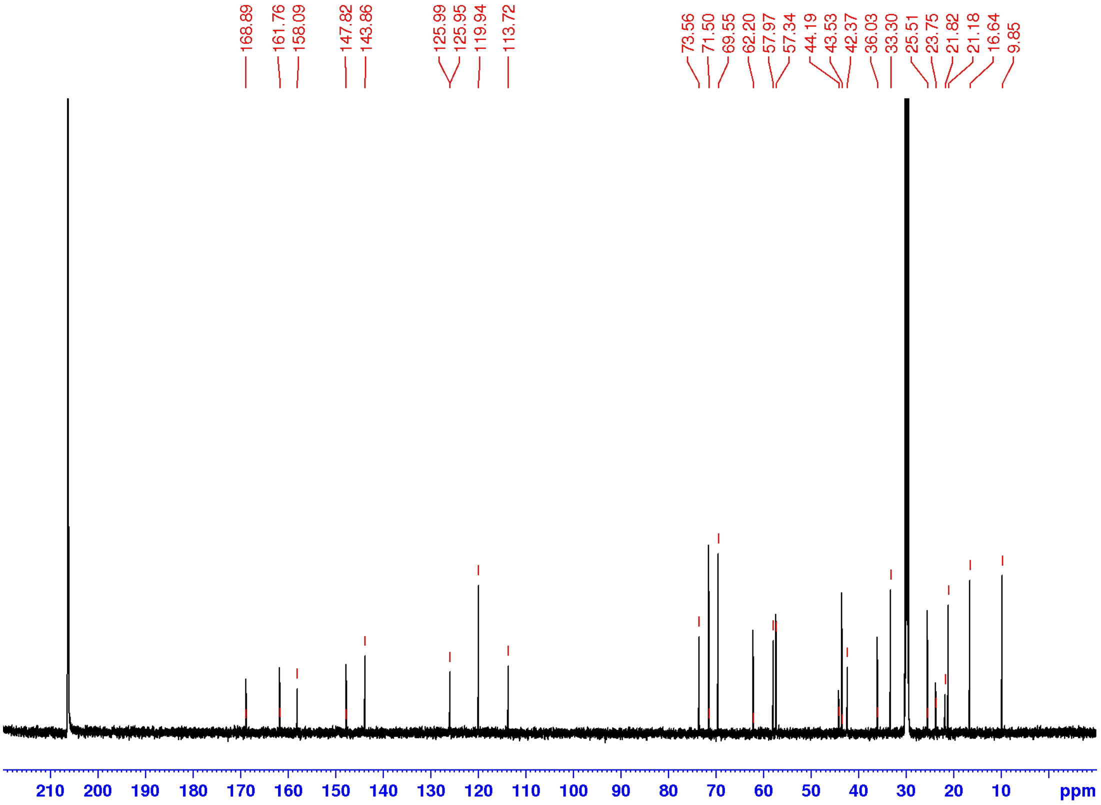

Figure S32. $^{13}C\{^1H\}$ NMR spectrum of **7′** in acetone-d_6 at 150 MHz.

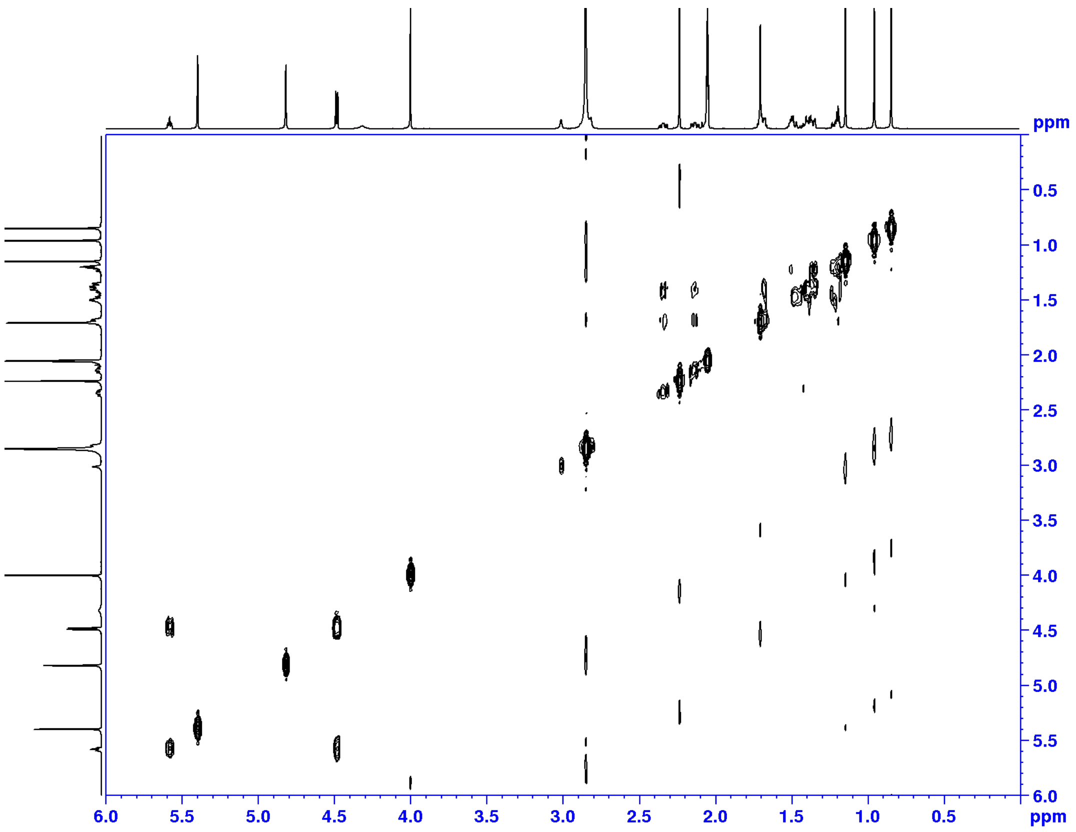

Figure S33. ^{1}H-^{1}H COSY spectrum of **7′** in acetone-d_6.

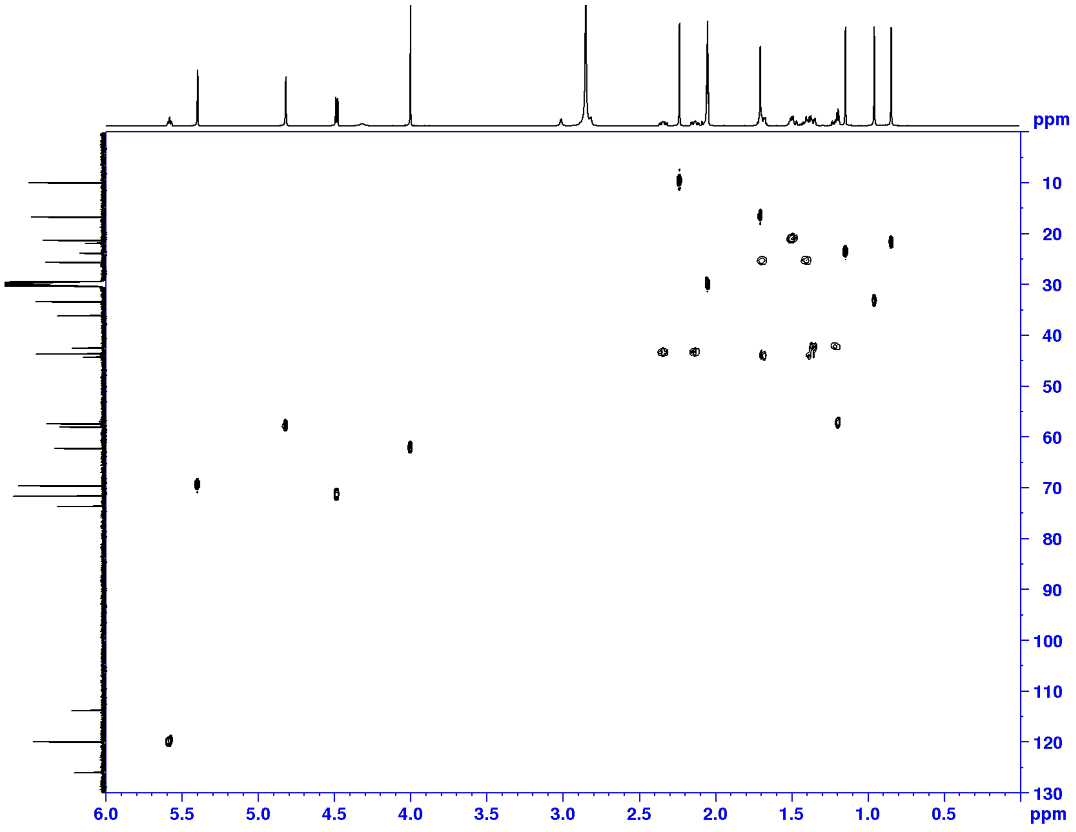

Figure S34. HSQC spectrum of **7′** in acetone-d_6.

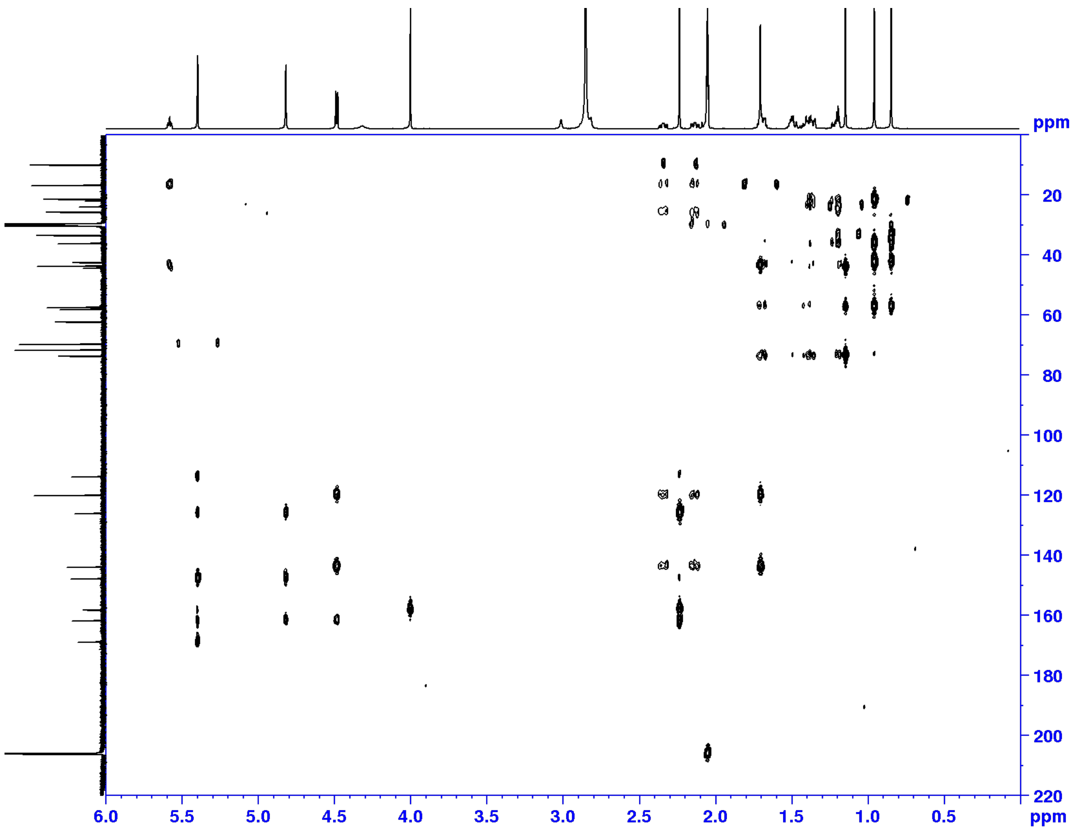

Figure S35. HMBC spectrum of **7′** in acetone-d_6.

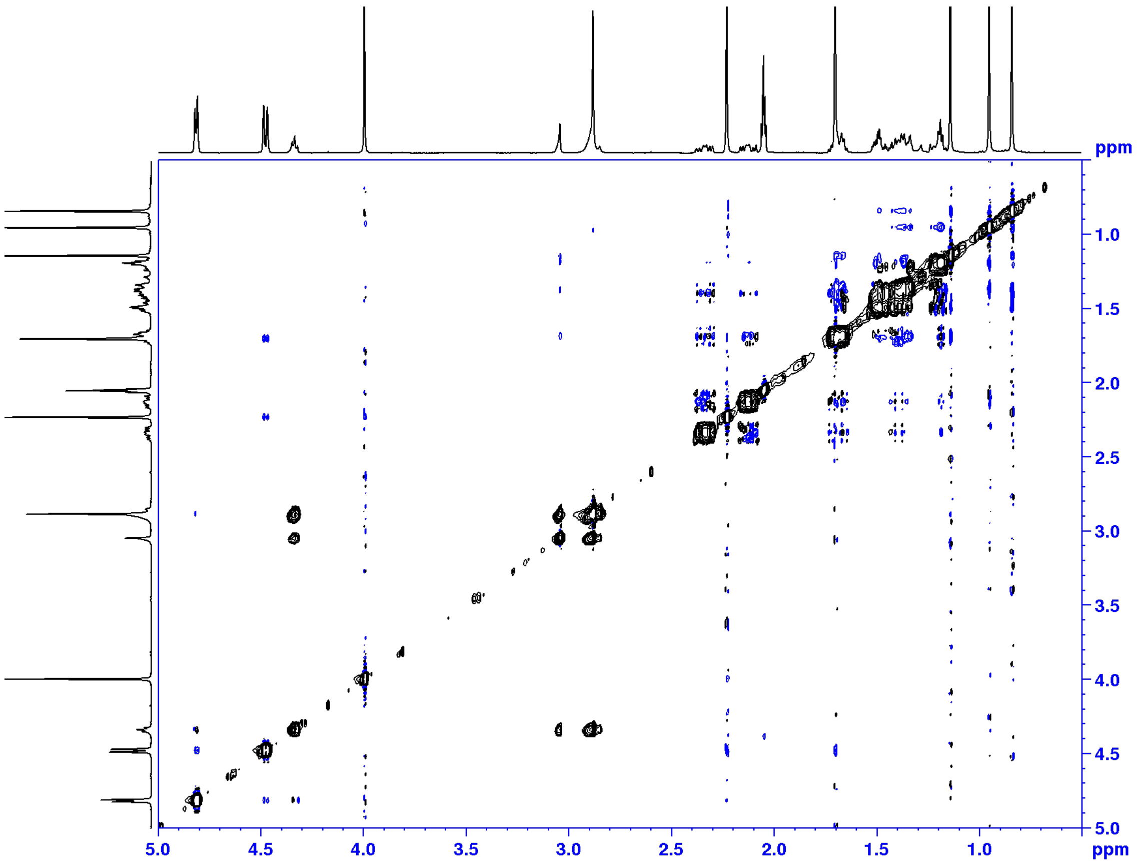

Figure S36. NOESY spectrum of **7′** in $CDCl_3$.

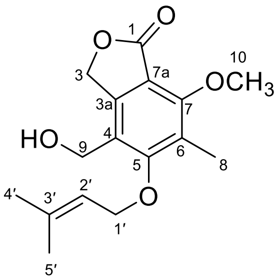

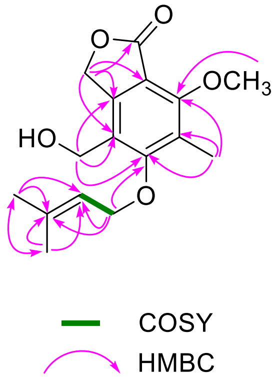

Figure S37. NMR data of **8′**.

position	δ_C, type	δ_H, mult. (J in Hz)
1	171.1, C	
3	70.5, CH_2	5.42, s
3a	148.5, C	
4	126.1, C	
5	162.9, C	
6	126.9, C	
7	158.9, C	
7a	114.0, C	
8	9.8, CH_3	2.24, s
9	58.0, CH_2	4.75, s
10	62.6, CH_3	3.99, s
1′	72.0, CH_2	4.44, d (7.2)
2′	120.9, CH	5.56, tsept (7.3, 1.3)
3′	139.9, C	
4′	25.9, CH_2	1.80, s
5′	18.1, CH_2	1.70, s

^{1}H NMR: 600 MHz, ^{13}C NMR: 150 MHz (in CD_3OD)

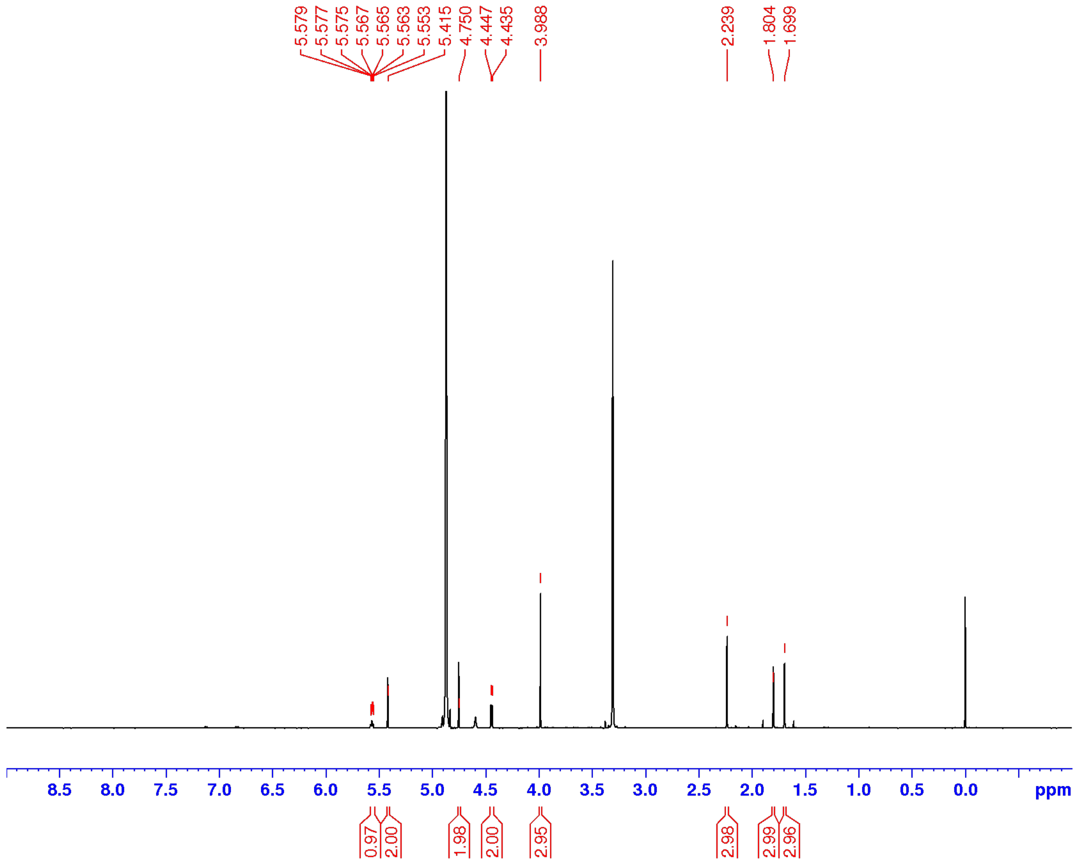

Figure S38. ^{1}H NMR spectrum of **8′** in CD_3OD at 600 MHz.

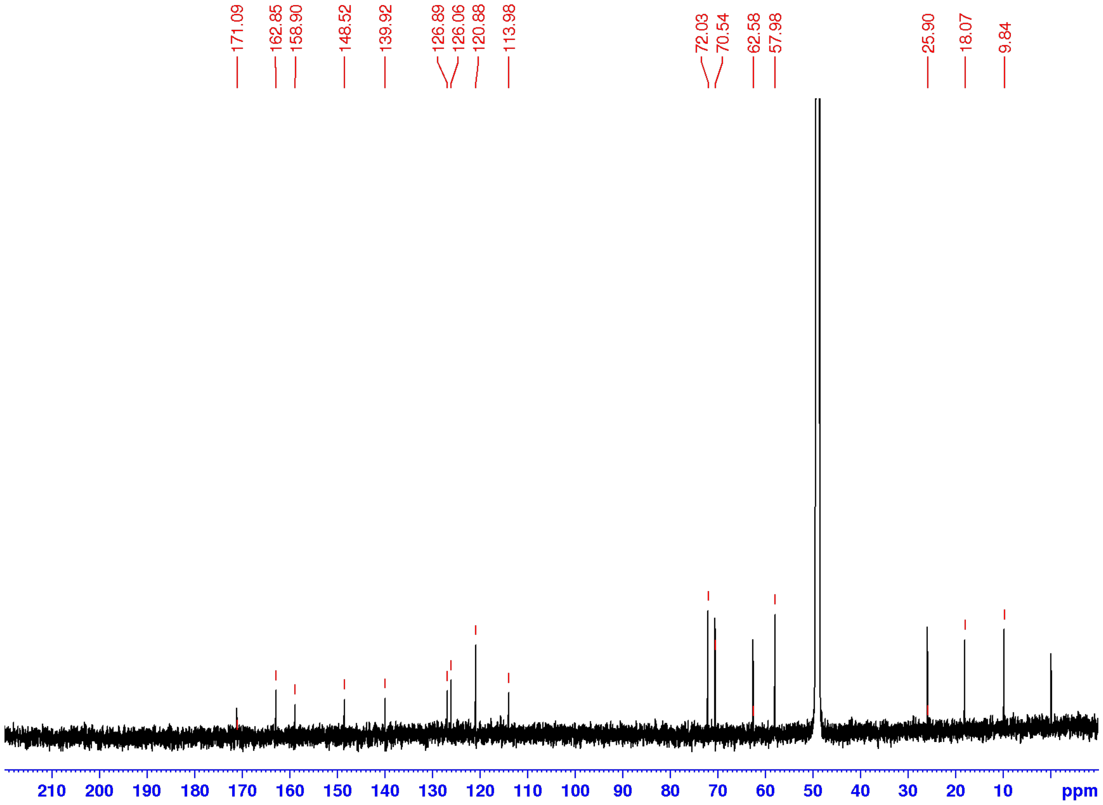

Figure S39. ^{13}C{^{1}H} NMR spectrum of **8′** in CD_3OD at 150 MHz.

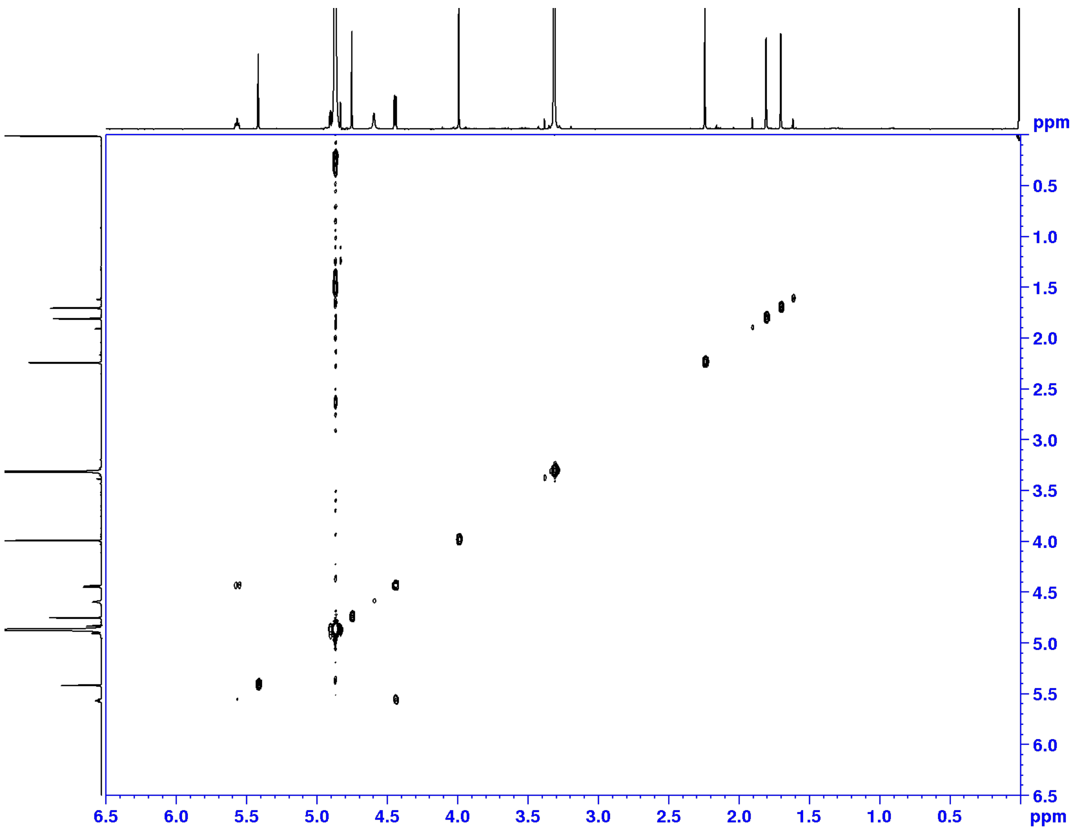

Figure S40. ^{1}H-^{1}H COSY spectrum of **8′** in CD_3OD.

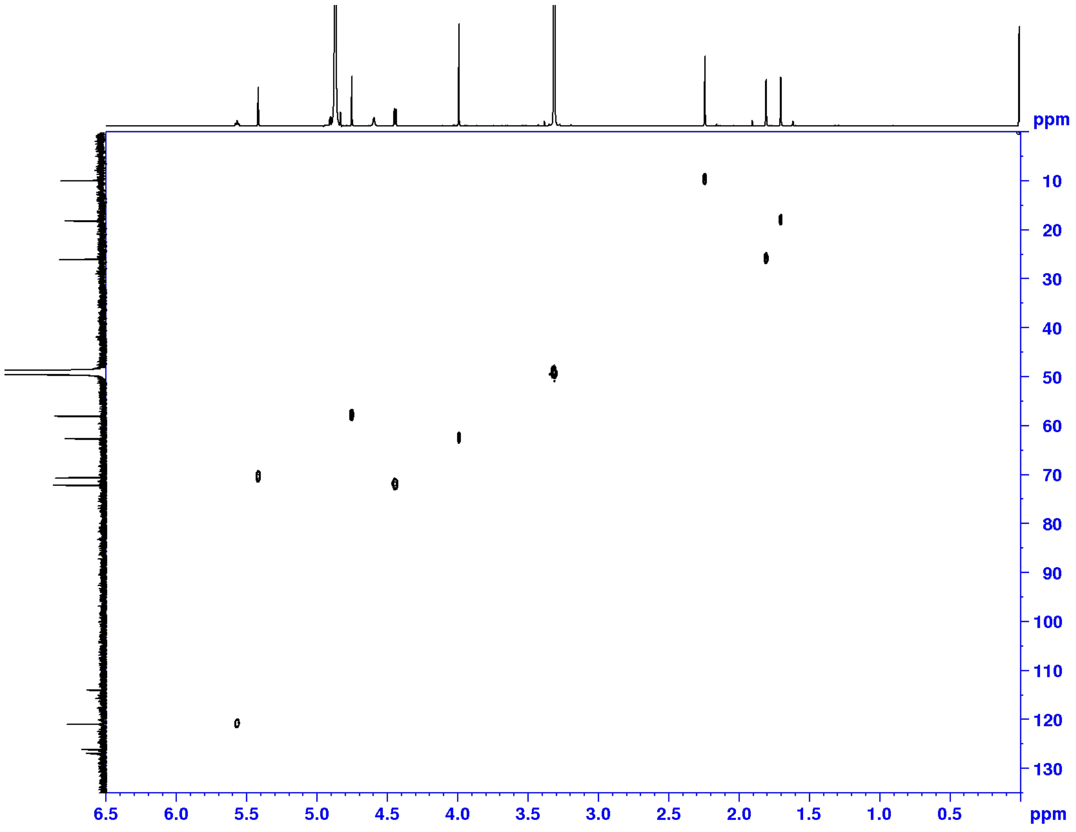

Figure S41. HSQC spectrum of **8′** in CD_3OD.

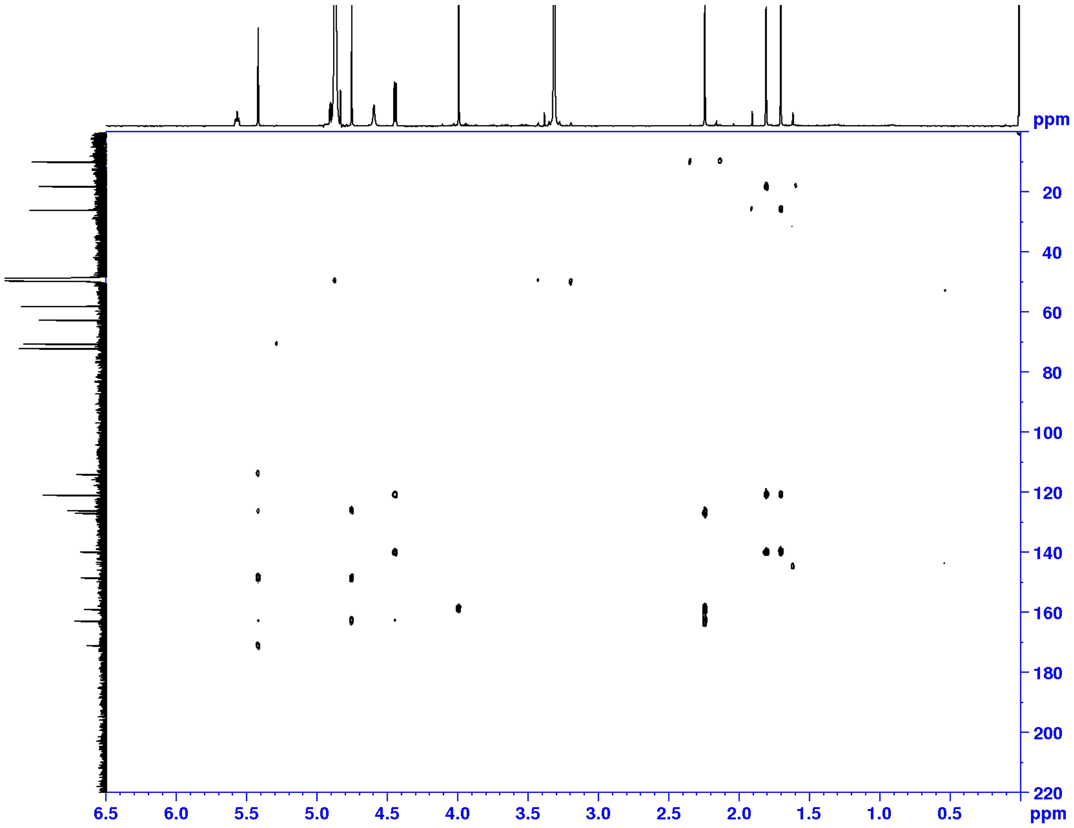

Figure S42. HMBC spectrum of **8′** in CD_3OD.

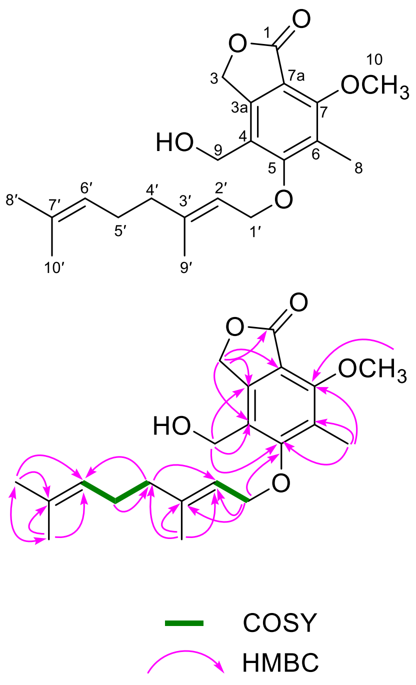

position	δ_C, type	δ_H, mult. (J in Hz)
1	168.9, C	
3	69.5, CH_2	5.40, s
3a	147.9, C	
4	125.9, C	
5	161.8, C	
6	125.9, C	
7	158.1, C	
7a	113.8, C	
8	9.8, CH_3	2.23, s
9	57.9, CH_2	4.81, d (5.2)
10	62.2, CH_3	4.00, s
1′	71.5, CH_2	4.48, d (7.1)
2′	120.7, CH	5.58, brt (7.2)
3′	142.2, C	
4′	40.2, CH_2	2.11, brt (7.5)
5′	27.1, CH_2	2.13, m
6′	124.7, CH	5.13, brt (7.2)
7′	132.1, C	
8′	25.8, CH_3	1.67, s
9′	16.5, CH_3	1.70, s
10′	17.7, CH_3	1.61, s
9-OH		4.28, t (5.2)

^1H NMR: 600 MHz, ^{13}C NMR: 150 MHz (acetone-d_6)

Figure S43. NMR data of **9′**.

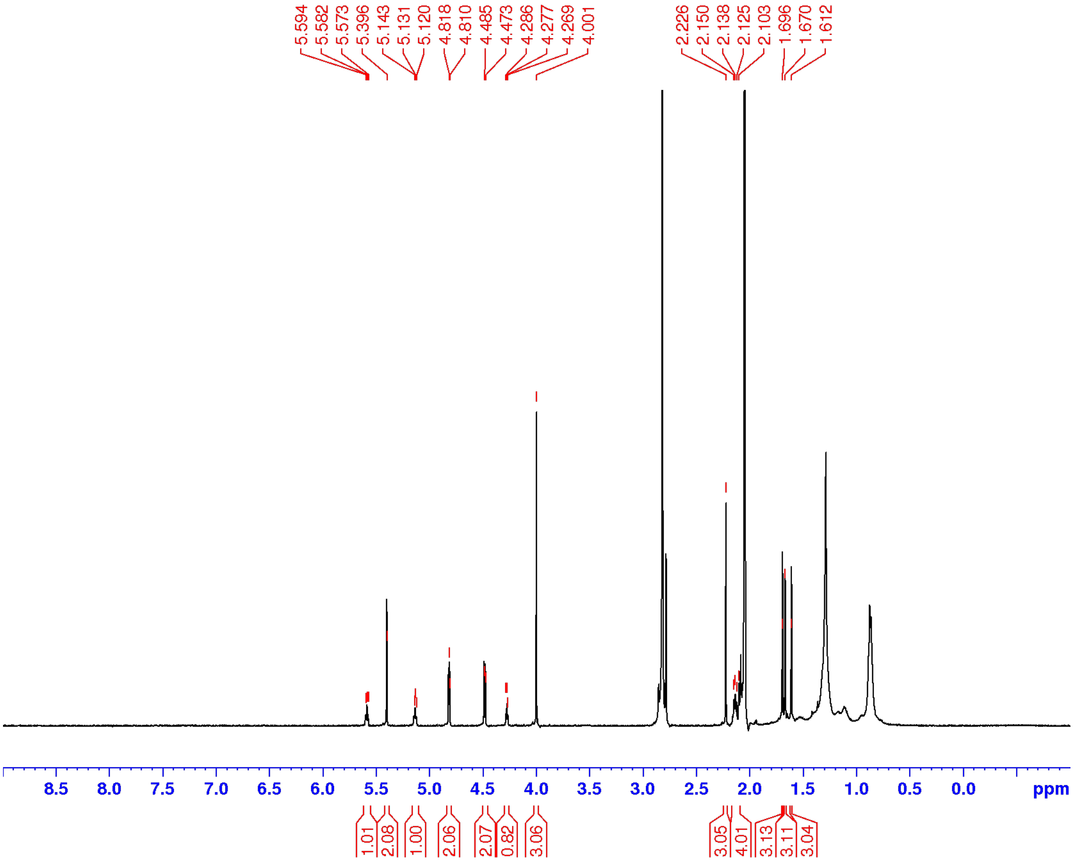

Figure S44. ^{1}H NMR spectrum of **9′** in acetone-d_6 at 600 MHz.

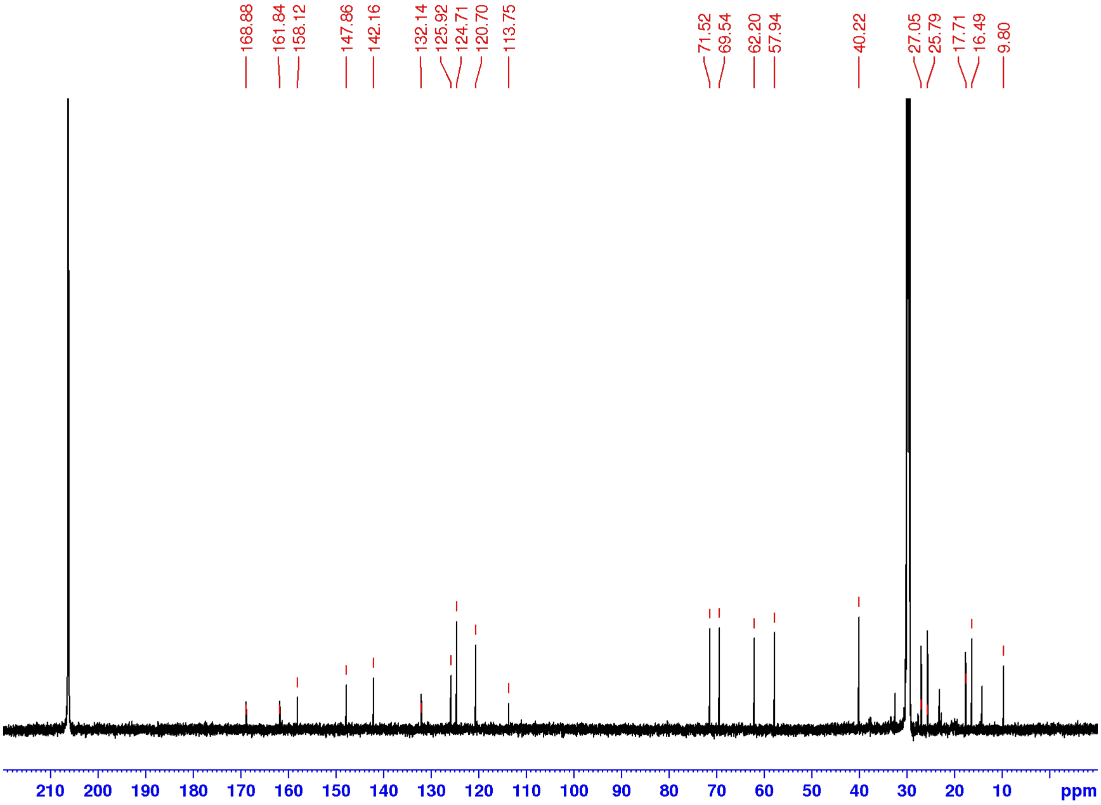

Figure S45. $^{13}C\{^1H\}$ NMR spectrum of **9′** in acetone-d_6 at 150 MHz.

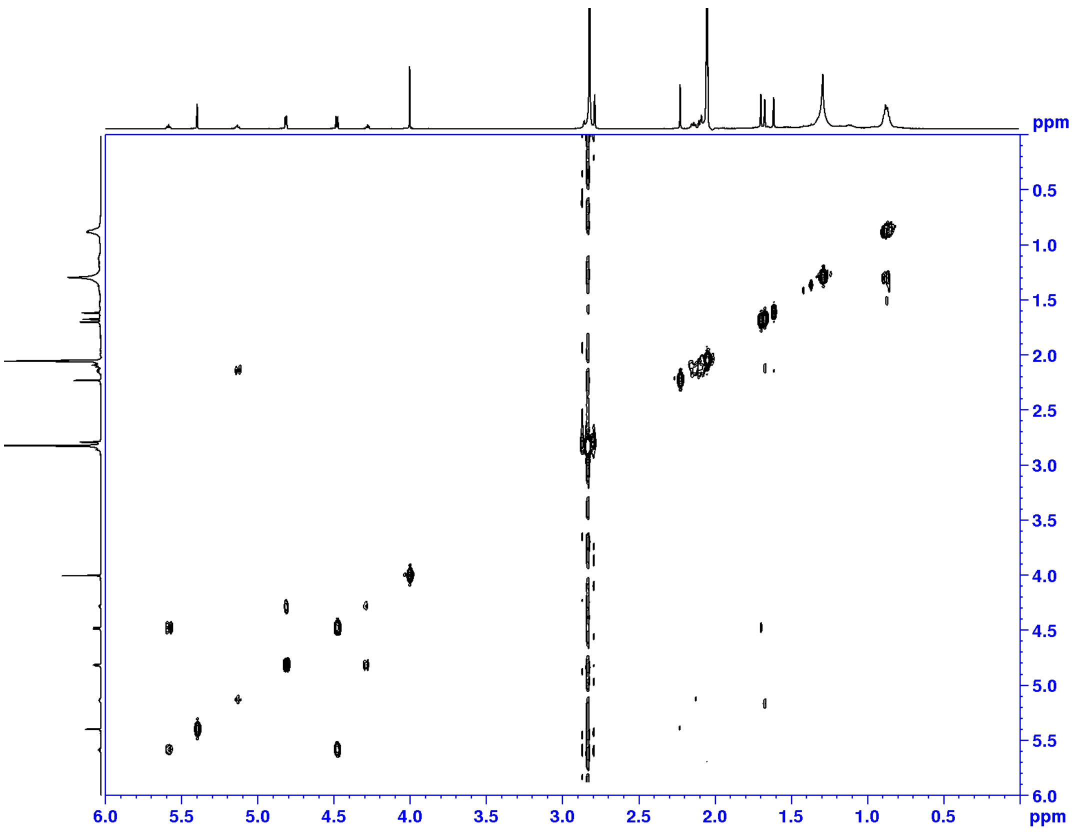

Figure S46. ^{1}H-^{1}H COSY spectrum of **9′** in acetone-d_6.

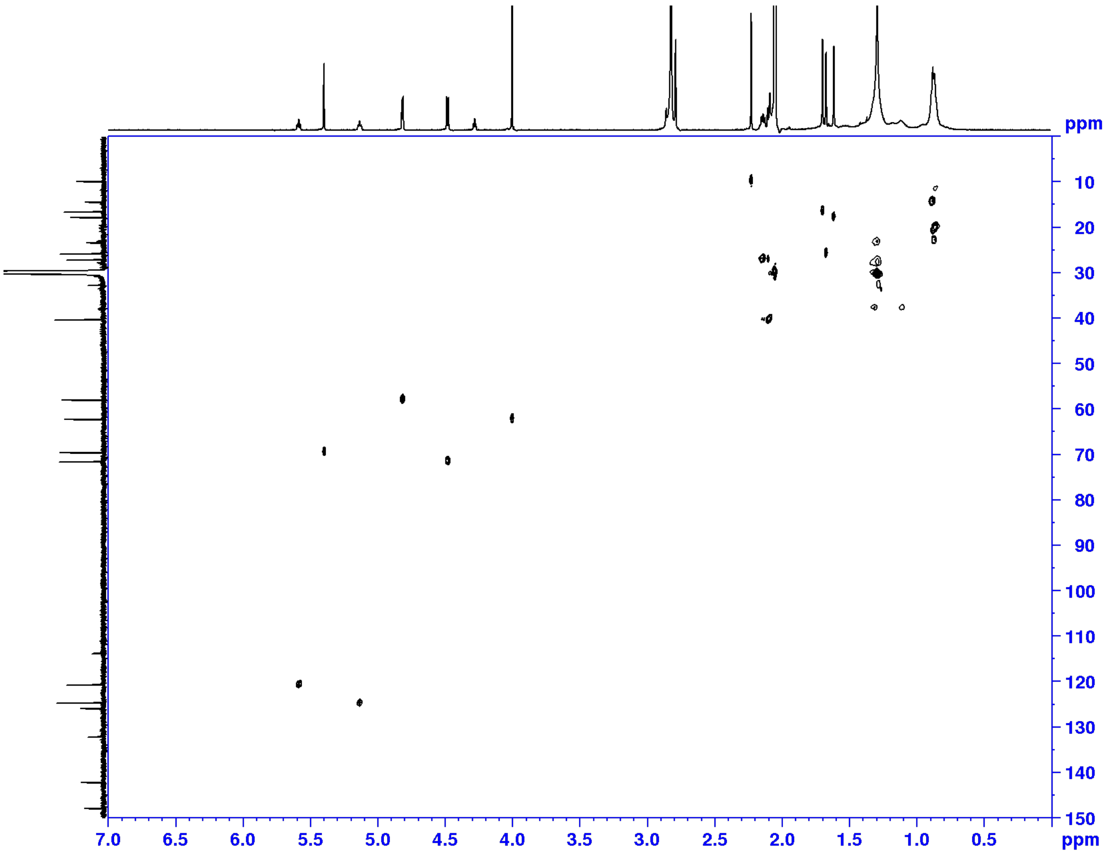

Figure S47. HSQC spectrum of **9′** in acetone-d_6.

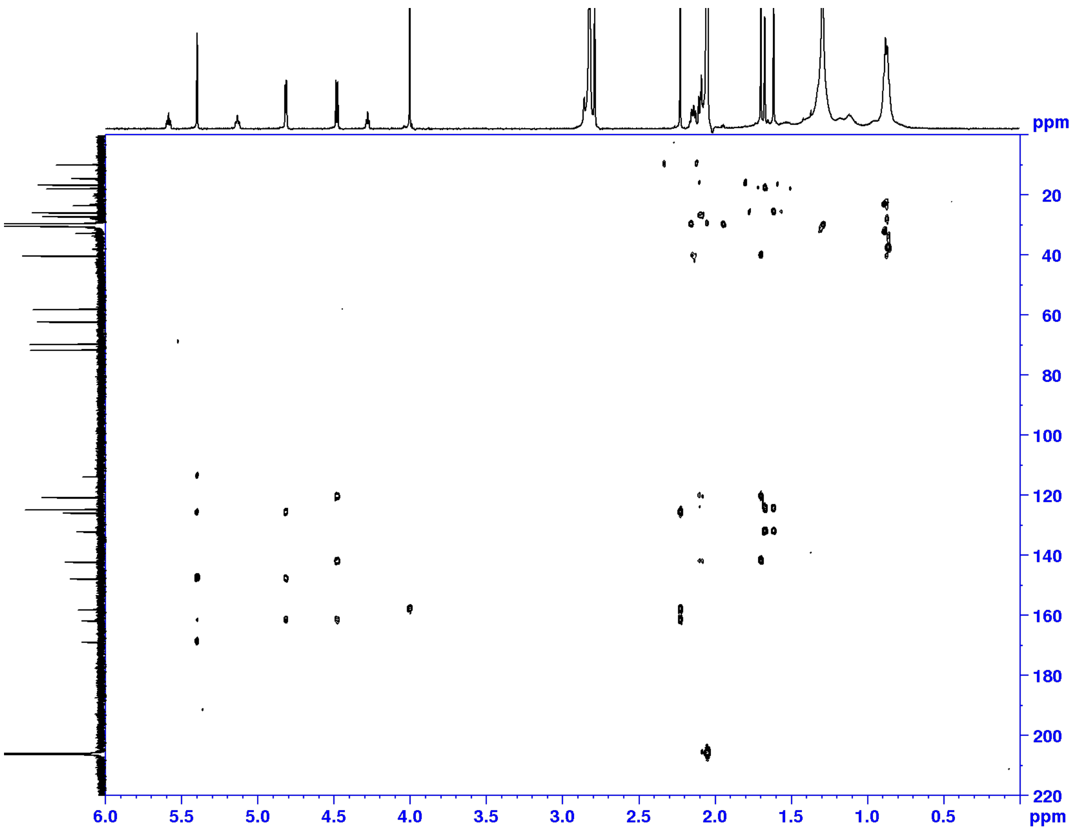

Figure S48. HMBC spectrum of **9′** in acetone-d_6.

Supplementary Data

Sequences of known Pyr4 homologues.

```
>Pru_AdrI
MEKSTLLSAVLKHRDALASVAEFLRILAGICWTLNYFSMLRTSQKDKIPSTGIFPLCNDIGWEFIYAFIYPKASAHWEGGVRVWFLVHCI
VIFFIIKNAHNEWDYFPLIQRNLYFLYGIVTIGFAIGQYSFAREVGPDLGFFYGGVLCQTLASLGPIAQILSRNSTRGASLLTWLLRAVA
TFGGFIKLTIYYLTGNAAGPWFESPMCKFYIGLTLILDFTYPICYYVIRRQELVNDEGDKKKKTKSGKAA
>Pro_AdrI
MEESSLLSAILDHRDALASVAEFLRILAGICWTLNYFSMLRTSRKDKIPSTGIFPLCNDIGWEFIYAFIYPTASAHWEGGVRVWFLVHCI
VIIFIIKYAHNEWDHFPLIQRNLYFLYGVVTIGFAIGQYSFAREVGPDLGFFYGGVLCQTLASLGPIAQILSRNSTRGASLLTWLLRAIA
TFGGFIKLTIYYLTGNAAGPWFESPMCKFYIGLTLVLDFTYPICYYVIQRQELANAQKEKKEKSK
>AdrI’
MDASNMSAVLEYRDELASVAESLRLLSGVCWTLNYFSMMYISGRDKVPNTNVFAITNDMAWEFMYAFIHPAASAHWEGGVKVWFLVHCAV
VLYILKFAPNEWDDVPIVKRNIYLIYTVSFLGFLAGQWAFAAEVGPDLGFFYGGVLCQTLASLGPNCQLLARNSIRGASLLTWSLRAVAT
FGGFIKLTIYYLTDVPAGPWFESPMCKFYIGLTLVLDFVYPFLYFSVWRQEAARTPSGKKIQ
>AtlC
MGDSTILSAVSEYRDELGAVAEFLRKLAGICWTLNYFSMMYVSGRDKIPNTGIFPLCNDIAWEFVYAFVHPAASAHWEGGVKIWFLVHLA
VVSYILKFAPNEWDDVPIMKNHIYLIYLVVTLGFTAGQLSFAAEVGPDLGFFYGGVLCQTLASLGPICQLLGRNSTRGASILTWLLRGIA
TFGGFIKLTIYFICDTPAGPWFESPMCKFYIGLTLTLDIIYPCLYHVIRRQERRIAVEKKKVK
>Trt1
MPSIISDPQAYDIMLRLLQFSCWSLSYINTVRTTLSDQLPSVSFMSICCDVAWEFVYAFVYPIASSHWAGGIRIWFAMHCVMLFIVAKYA
PNDWDHVPLMKRFARLAYVAITIGFMAGHLALASEIGPALGFFWSGALCQITASLGSLCLLVCRGSTRGASIKTCYFRIIATVGGFTKMS
IRHIWHLADEPWFDSPLCWFYIAITLTLDAIYPVFFFYFRAIEHPKKDSERKVE
>An_AusL
MSQLTISKIIEEPFSALSLSEMLKILAALGWSTNYLAMVYRTQADKLPAIAVLPLCCDIAWEFTYAWIYPQASGHWQGVVRVWFFLHTAV
LAATLRYAPNDWAGTPLGESRGRLVLLYAAVIAAFAAGQLCLALEMGGALGFHWGGALCQFLSSSAAVGQLLTRGHTRGASLLIWGARAI
STAGGFVKLCIRFQHQVDGAPWLDSPMCWFYIGIVLSLDASYPVLYQLTRRHEEASGGGKSGKVKNW
>Pb_AusL
MEEPLTVAAIFRGPFNILAISEVLKVVAAVGWSVNYIGMVHRAWKDQIPSIGILPLCCDIGWEFVYAWMFPDFSSHWQGVVRVWFFLHSA
VLLVTLKVSPNDWANTPLAHRHIVFIYIFVTIVFGAGQYALAAEIGPALGFHWGGALCQFLSSSGGIAQLLSRGHTRGASYLIWFARAIS
TFAGFIKLCIRFQHNVDGAPWLDSPMCWFYIVTVLSFDAAYPFLYFSMRKFETPAPQREARIKKQ
>Ac_AusL
MSHLTVSKILEDPFSALSLSEMLKILAALGWSTNYLAMAHRTHADRLPAIAVLPLCCDIAWEFTYAWIYPQASGHWQGVVRVWFFLHTAV
LAATLRYAPNDWAGTPLGKSRARLVLLYVAVIGAFAAGQLCLALEMGGALGFHWGGALCQFLSSSGAVGQLLTRGHTRGASLVIWGARAI
STAGGFVKLCIRFQHQVDGAPWLDSPMCWFYIGIVLSLDASYPVLYQLTRRHEEASGRGNSGKVKNR
>PrhH
MEEPLTVAAIFRDPFNILAISEVLKVVAAVGWSVNYIGMVHRAWKDQIPSIGILPLCCDIGWEFVYAWMFPDFSSHWQGVVRVWFFLHSA
VLLVTLKVSPNDWVHTPLGHRHIVFIYIFVTLVFGAGQYALAAEIGPALGFHWGGALCQFLSSSCGIAQLLSRGHTRGASYLIWFARAIS
TFAGFIKLCIRFQHNVDGAPWLDSPMCWFYIVTVLSFDAAYPFLYSSMRKLETPALRKESRIKNQ
>InsB2
MSASDVFIGPWDLHTIDISLRWVAFICWSLNYISLLGTAIRDRTPSMALLALCSDTGWEIVYGFIFPEASRHFGSGVRVWLLLHVPVVYV
MLKFGADEWDHNPLVKKNLPLVYVALTFGFGAAQMALANEIGPDLGFFFGGVFCQTLLIFSHLCQLLSRGSTRGASYSIWFFRCVGFIAG
FSKLILLDLHGHNEVPWLGSPICWFYMAASVVLDIIYPVCLYFMRREEQSGEQRKNKVD
>InsA7
MSSTPLSLDNIIEYPIFDYVAITSRALCGICWVITYVLMIHKSFQQRTYSIPIITLLFNIGWEFIYTLVYGDTDVRSQGIVRLWPLFNLA
VLVVTIKFAPNEWEHSPLVRRNIPLIFVLGLAWSLSGHWALASEMGPGLAFYWSGAGCQILTSVGLLCQLLCRGSTRGSSYLIWFMRLLG
SSCGYVNRFIMASRGLDKFPFFKSPVMKWYMVTVIILDAAYGVCFHIINRQERSRPVTARGKWM
>Pyr4
MDGWSDLSSAPPQYREVAGIADWALLAQGLGWSINYLAMIYHSYKDRTYGMAILPLCCNFAWEFVYSVIYPSHNSAERAVLTTWMILNLF
VMYTAIKFAPNEWQHAPLVRQCLPWIFPVAIAAFTAGHLALAATVGVSKAANWGAFLCFELLTSGAVCQLMSRGSSRGASYTIWLSRFLG
SYIGGIFLHVRETHWPQEFGWISHPFVTWHGLMCFSLDIAYVTFLWRIRRQEHRSQRKKAL
>Ppb5
MDGWSDISSAPAGYKDVVWIADRALLAQGLGWSINYLAMIYQSRKDRTYGMAILPLCCNFAWEFVYTVIYPSQNPFERAVLTTWMVLNLY
LMYTTIKFAPNEWQHAPLVQRILPVIFPVAIAAFTAGHLALAATVGVAKAVNWSAFLCFELLTAGAVCQLMSRGSSRGASYTIWVSRFLG
SYIGSIFMHVRETHWPQEFDWISYPFVAWHGIMCFSLDISYVGLLWYIRRQERQGQLKKAM
>AstH
MDWFIGAIPPPEYQAVAWFANVATIIETIGWAINYGDSIHRSVKDRTYGMAIFPMCLNLSWEIVYTLVYPCNDTYQWIMIVTWLLLNPGI
MLCAIRYAPNEWQHAPLVRENIPLIFALTMVACILGQLAAAATLGPGVAATVVACFCYLLLTVGSLCQLIIRGSSRGTSYTMWASRFIGN
LAAGFNAHFRVTYWPQVFGFLDTALMKWFVATTTIVELCYIFVLRHIRDKEAASHNTTNLADKKR
>CdmG
MDYFYGTSPPPEYERYASIVDAATLVQGFLWALNYGEASYRSIKDRTYGMAIFPLCCNYAWELVYTVIYSSQNKYERIIMTTWLILNSIM
MGFTIKFAPNEWRHAPLVQRNIPFIFLAGVAAFVIAQLALAATVGPGLAMNWVAALCYLLLTIGSLCQLMTRGSSRGVSYTMWLSRFVGT
YVGVICVYFRYNYWPQNFSWVDEPIMKCFSGISLAVEIVYGVTLWHIRKQERHHIVEKSK
>AndB
MQPITQIPLTFDTVVNLLGSASGIGWILNYILMTYYSFRDKTYSMSMLPLCCNIAWEFVYGILCPSSTFVVRPVILSWLVLNCLVVYAAI
KYSPNEWAHAPLVQRHLPLLFTVGIAACTGFHIALIRKFDPATAFLWSARSCQVLLSIGGLFQLLCRSSTKGGSYVLWLSRFLGSICGVL
```

KMTLMWKYGESRFPWLDDPLTAYCIALWIISDVLYGVVFYSLRSKELAGAGKAKAI
>FncH
MKTLLKTLSLINSAGWMINYIGMVYLSFKDQTYSMAIIPLCCNVAWEIVYGIIQPSSIIVPRKVILTWLALNCAVMYAAIKFAPTEWSHA
PLVMENLPLIFAIGVAVFIAGHLALAAELGPQTAFLWSARACQTLLSVGALLQLLGRGSTRGASFLVWFSRFFGSACGVVRLSVMYLRGM
HSFAWFNSPLTMWCVVLFLLSDMLYGVCFYYIRGRELNPPV
>NvfL
MDPWIALSSPLTSQTVEYVANTLRIMCAISWNISYMSMAYYSFRDKTYGNALIPLCNNIAWEFVYSFIHCPKLTFVRIENTGWFLLNIVV
MYAAIKYSENEWKHAPLVQRNLPFIFAVGISAMIAGHLALAAQIGPRIAFVWSAKGCQLVLSTGALSQLLSRGSTRGGSYVVWLSRYLGT
VFIDVMVTIRHVYRAAGPSWPSSPLLLWFMAVFHLLDWTYGFCFYHIRRQELVSEAAEKDKDK
>SetH
MDMNSMDFSKAPPGFQEVRWISNILLTTLASGWLVCYIATIRTAFRDRACWMPLIPLSCNLSWELVYVFLYPPPRLPIVTFWLMLNLVVV
YVAVRFASEPGQHTAAASKRSLAVFFVLGALFWGAGHVALISQVGPLTAFYYGGLGCQIMTSATALCRLVDRGCTTGASYIIWVARVIGT
SSALLGVYFRAHYWPELWAWAVNPLMHWCTAAFVILDGLYGVCFWYIRQSETSQQKVLDAKKD
>BrvF
MDDMDFSKAPPSFQEVRWISTILLTTLASGWLVCYISTIAKAFRDRACWMPIIPLSCNLSWELVFVFLYPPPRLPIVTFWLSLNLAVVFV
AVKFASGFNKQSAVFSNGALQRGAFFVLATIFWGAGHVALISQVGPLTAFYYGGLCCQIMTSSAALDKLLKAGHTTGASYTIWISRVIGT
SSALLGVYFRAYYWPEVWAWASNPLMYWTTAAFIILDGWYGVAFWNIRRSEKQQQKRHATKKN
>OlcD
MESLDFTKAPPEFQEVKYLSDILLVTLASGWLVCYFATIRTAFCDRACWMPLLPLSCNVAWELVFITLYPPPGSPILGFWLLVNLGVIYS
ALRFAPSKASHLAVEKHYLPVLFVLAVGFWAWGHLALIEQLNPLPAFYYGGMACQLMTSAAALSGLVDQGSTQGASYTIWLSRVIGTSSA
LAGLFFRAHYWPSLWAWADNELMRWLAAAFGILDGVYGVQFWRLRRSENQLTIDHAHRKTE
>OlcD’
MDSLDFTKAPQSFQSVKWISDTLLFAMAAGWLTCYIATIRTARRDGACWVPIVPVSCNLAWELVYAILYPPPRLLILTAWFTLNLVVVST
ALKYSPESRGSSPLRGYRLHVCFVIVTALWAAGHICLAKVVGPLTAFYYGGLACQIMTSATALCGLVRSKRSSGASWLIWASRVIGTGSA
IAGVLFRAHYWPELWAWTMNPLIYWAAAAFAIFDGAYGFCFWSVRQAEVRAGIKDD
>Cle7
MEEGWDFDSSPPSFKQVQPLLLTLFSLSGTGWLINYITTIRTAYRDRTPGVSLVALTNNLAWELVFAILHPPPLPVAKVILRSWLFVDIF
VIYTTAKFARSSSISSNAPLLHRYLHLFVLFGILGFFSGHWALSVLLSPIKAFYWSGMMCLVVMSGTALGILVQRGHTRGMSYGMWFSRF
VGSIFAVASLFLRSTYWPQVWGWSDNILMRWFAGAFVVLDGLYGVCFWYTRRVEQRQRDKVA
>Sre3
MDAFDLSSAPAEFRAIKPICDMLVIFTGATWVINYIVTVRQIFRDRVCVMPLVCLCCNVAWEITVVLIHRPPYFLLDVFFAMWLSVNMVI
VYGSIKVSMEKQLPSPLMHKHLPLIIALTILGFILGYHALAKMLGPTKAVWWGGMLSQVVMSADCLGQILHRGSTHGASWAMWTSRVLGS
YSAIAGVWVKSWFWPQQWDWFDNALTRWITGISLIMDIMYGCIFWFIWQTEKGAKVQKA
>EsdpB
MDEFDFNAAPPEFQEIRWMVASIFLISGTGWIVNYVTTIRTALRDRTSGVTLLSLCNNLAWETVFAVIHRPPHLIAALVITVWLLVNIYV
IYVSVKFARESQDVSPLLRRHLPVVTLLGFVGFLTGHIALSMHLGPTKALYWGGMICQVTLSASALGLLIQRGHTRGASPAMWLSRFIAS
SFGVPGLFVRAVYWPSAWGWADNILMRWLSGVFFLLDLSYGAIYYHISRSEHEVGASGSKMSKRE
>SubB
MNAADISRAPPGYLEVAWIADTCKLLMGLGWTTNYAGMIYKSLKDRTYGMALMPLCCNFAWELTYAVIYPFGSRQDKFTHYFGLMLNCGV
MYTAVKNAEREWTHAPLVRRNLPFIFIICIAAWTTAHLALALQIGPSHAQAFSAYGCQLLLSVGALCQLLSRGSSRGASYFLWFCRFFGS
LVLIPQDVLRYQYWRQDHEYMGSPLYIWFVSIFLLLDGSYALCLWYVRRFESEQEEAKKAKSI
>DpmaB
MNAADISRAPPGYLEVAWIADTCKLLMGLGWTTNYAGMIYKSLKDRTYGMALMPLCCNFAWELTYAVIYPFGSRQDKFTHYFGLMLNCGV
MYTAVKNAEREWTHAPLVRRNLPFIFIICIAAWTTAHLALALQIGPSHAQAFSAYGCQLLLSVGALCQLLSRGSSRGASYFLWFCRFFGS
LVLIPQDVLRYRYWRQDHEYMGSPLYIWFVSIFLLLDGSYALCLWYVRRFESEQEEAKKAKSI
>DpfgB
MEVADPSRAPPEYKDVAWIADTCKLLMGIGWTTNYVGMIYKSLKDETYAMALMALCCNFAWELTYALIYPFGSDLEMYVHFSGLMLNCGV
MYTAVKNAHREWGHSPLVLRNLPLIFIICVSGFMSGHVALAAQVGPSLAQAWSAYGCQLLLSVGGLCQLLCRGHSRGASYFLWFSRFFGS
LVLVPQDILRYKYWRVDHEYMGSPLYIWFVCIFLLLDGSYGICLWYVRRFERQTAVAHKKKK
>DpchB
MNVADISQAPEAYRDVVWIADTCKLIMGIGWTANYVGMIRKSLKDQTYAMALLPLCCNFAWELTYAIMYAFTTSLEKYVHFSGLLLNCGV
MYTAVKNAPREWEHAPLVQRNLRLIFVLAVAGFASAHVVLAKQVGPELGQAWSAYACQLLLSVGGLCQLLCRGHSRGASYFLWFSRFFGS
LVLVPQDIIRYTYWKEAHEFMGSPMYIWFVTIFLILDGSYGLCLWYVRRFEQQNPAAGKLKK
>DpmpB
MNIVPLSQAPPEFLEVAWLADACKLLMGVGWTANYIGMIYKSIKDRTYGMALMPLCCNFAWELVYALILPFDSGMEKWVHVTGLAFNCGV
MYTAIKFAPGEWAHARLVQRHLTWIFIASVAGWMSAHLALAAQLGPSLAQAWSAYGCQLLLSVGGLCQLLCRGHSRGTSYLLWFSRFFGS
LVLIPQDILRYKYWRRDHEWMKSPLYLWFVSIFLILDGSYGILLWYVRRFERETAEAENRKRR
>DpasB
MDVHDLTRAPPEYLEVVWVTDVCKLVMAVGWLSNYIGMIAKSIKEQTYSMALMPLCCNFAWEFTYFFIYPYKVPMERNIHTLAFLLNCGV
MYTAVRYGAREWGHAPLVQRNLPVIFVVCIACWVSAHVAFAEQYGPSLAQAVSGFACQILLSAGGTCQLLCRGHSRGASYKLWLARFMGS
FALILPNMLRYKYWRDDHQYIGSPLYIWFLGMFLFLDGSYGFVLWYVRRHEREQVLVAKPKVQ
>JanB
MDGFDVSQAPREYQAVKPLADLFVLGMGLGWVINYVGMVYTSFKERTYGMAIMPLCCNIAWEIVYCVFHPSKSRVELGVFAMGLLINFGV
MYAAIIFSSREWSHAPLVERNLPWIFCIGVLGFLTGHLALAAEIGPSLAYSWGAVVCQLLLSVGGLCQLLCRGSTRGASYTLWLSRFLGS
CCTVGFASLRWMYWPQSFAWLNSPLVLWSLAVFLMVDGSYGVCFWYVEQYEKSVLMGRATKAM
>AceB
MDGFDASQAPPEYLAVKPLADLFVLGMGLGWVINYVGMVYVSFKERTYGMAIMPLCCNIAWEVVYSLIYPSKSLVERGVFATGLLINLGV
MYAAISFSPQEWSHAPLVERNLPWIFALGTIGFLTGHLALAAEIGHSLAYSWGAVVCQLLLSIGGLCQLLCRSSTRGASYTLWYGLSRFL

GSCCTVGFASLRWMYWPESFAWLNSPLVLWSLAVFVMVDGSYGVCYWYVKGYEKSVGIVRARKIT
>PenB
MDGFDVSQAPPEYRAVEPIANIFVLGMGLGWLINYVGMIYQSFKDETYGMAIMPLCCNIAWEIVYSLIYPSKSLIEQGVFIAGLTINIGV
MYAAIKFAPKEWSHAPLVMRNLSLIFFLATLGFLTGHLALAAEIGHSLAYSWGAVVCQLLLSVGGLCQLLCRGSTRGASYTLWLSRFLGS
SCTVAFASLRWMYWPESFSWLNSPLVLWSLALFLTVDGSYGLCYWYVRQYELSLKEAEGRKSK
>PtmB
MDGFDVSQAPPEYRSVEPIANLFVLGMGLGWLINYVGMIYQSFKDETYGMAIMPLCCNIAWEIVYSLIYPSKSLTEQGVFIAGLTINIGV
MYAAIKFAPKEWSHAPLVMRNLSLIFFLATLGFLTGHLALAAEIGHSLAYSWGAVVCQLLLSVGGLCQLLCRGCTRGASYTLWLSRFLGS
SCTVGFASLRWMYWPESFSWLNSPLVLWSLALFLTVDGSYGICYWYVRQYELSLKEAEGRKSK
>EstB1
MDGYDVSQAPPEYHAVKPLVDSLILGMGLGWMINYLGMVYQSFREETYGMAIIPLCCNIAWEIVYAVIYPSKDSHERGVFFGGLIINLAI
IYAAIRFSPNEWAHAPLVRDNLRWIFLVGILVFLTGHLALVAEMGFSLAYLWSAAFCQVVLSLGGLCQLLCRNRTRGASYLLWGSRTFGT
FSGVVALFIRWKYWPESFEWLNSPLMLWCLTVSLLFDGSYGVCFWYVKRSEEMLLAQRKER
>EstB2
MDGFDVSQAPPEYHAVKPIADLFILGMGLGWVINYIGMVYRSFQEKTYGMAIIPLCCNIAWEIVYAVIYPSQSVAERGVFLAGLLINFAI
IYAAIRFSPNEWAHAPLVRDNLPWIFLVGISGCLTGHLALAAEIGPSLAYSWGAVICQLLLSIGGLCQLLCRNRTRGASYLLWLSRFLGS
CCTVGFASLRWKYWPESFEWLNSPLVLWSLAVFLLVDGSYGIFFWHVKRYEKMTLSQQKAK
>DesB
MDGFDVSQAPPEYHAIKPLADLFIFGMGLGWVINYVGMVYRSFQEKTYGMAIIPLCCNIAWEFVYAVIYPSQNVAELGTSIVGLLINFAI
IYAAIRFSPNEWAHAPLVRDNLTWIFLLLIFGFLTGHLALAAQIGPSLAYSWGAMVCQLLLSVGGLCQLLSRHRTRGASYVLWSRFLGSC
CVVGLASLRWKYWPESFEWLNSPLVLWCLAVFLLVDGSYGIFFWHVKRHEEFLLSQKKEK
>PaxB
MDGFDVSQAPPEYQAIKPLADLFVVGMGVGWIINYIGMVYISFKHETYGMSIMPLCCNIAWELVYCLVFPSKSPVERGVFWMGLLINFGV
MYAAITFSSREWGHAPLVERNISLIFFVATMGFLSGHVALALEIGPALAYSWGAVICQLLLSVGGLSQLLCRGSTRGASYTLWASRFLGS
TCTVGFAGLRWMYWSEAFGWLNSPLVLWSLVVFLSIDGFYGICFWYVDRNEKSLGISGPKKAN
>AtmB
MDGFGSSQAPAAYREVEWIADVFVIGMGIGWIINYVGMVYGSLKGRTYGMAIMPLCCNIAWEIVYGLIYPSKTLYEQGVFLSGLTINLGV
IYTAIKFGPKEWTHAPLVMHNLPLIFMLGILGFLTGHLALAAEIGPALAYNWGAAFCQLLLSVGGLCQLISRGSTRGASYTLWLSRFLGS
FSVVISAWLRYKYWPQAFSWLGKPLILWCLFAWLVVDGSYGVCFYYVKRYERRIGHDSDRKTV
>NomB
MEGFGSPQAPDAYREVAWIADIFVVGMGIGWVINYIGMVYGSLKDRTYGMAIMPLCCNIAWEIVYGVVYPSTTVAEQGVFVCGLAINLGV
IYTAIKFGPKEWTHAPLVMHNLPLIFMLGIIGFLTGHLALAAEIGPALAYNWGAAFCQLLLSIGGLCQLITRGSTRGASYTLWLSRFLGS
FSVVISAWMRYKYWPQAFSWLGRPLLMWCFFVWLVVDSSYGVCLYYVQRYERRVGLGADRKTV
>NodB
MDGFDRSNAPVEYQRVEWISDIFVFGMGVCWLINYAGMIYTSLQEQTYSMAPLALCCNFAWEMVYGLIYPSKSRIEQGVFLAGLVVNLGV
MYTAIRFAPNEWAHAPLVMNNITLIFALGVLGSLTGHLALAAEIGPALGYSWGAVACQLLLSVGGFCQLLGRSSSRGASYTLWLSRFIGS
GCVVGFAILRYMYWSEAFNWLNSPLVLWSLGVFIAVDSLYGICLWNVKKYEHGQERSNARKAQ
>LtmB
MDGFSNMEQAPLAYQEVQWLAETFVTFMGLGWLINYVLMIWHSRRGEPSSMALIPLCNNIAWELVYTIIYPSPNKVELAAFIAGVTLNFL
IMTSAARSARSEWSHSPTMAKHAGLIIVAGILMCFTGHVALAMEIGPALAYSWGAVICQLALSIGGVCQLLQQHSTGGTSWKLWSSRFLG
SCCAVGFAFLRWRYWPEAYGWLASPLILWSLATFLVADLTYGVCLLL
>TerB
MDGFTDSQAPPAYEEVKWLADTLVAFMGIGWLINYGAMIRHSYQGRTYCMGIIPLCNNIGWELVYTLVHPSSNRVELAVFAAGVTLNVII
MFAATRSAKTEWQHSPLVANHTPLIFLGGTFVCFAGHVALAAEIGPALAYSWGAVICQLVLSIGGVCQLLQRNTTRGTSVTLWLSRFLGS
CCTVGFAFLRWRYWPEAFAWLAGPLVLWSLATFVLADITYGVCLYLISQTETSSTKRSKLK
>IdtB
MDGFSILHEPPAAYKEVKWMADTFVAGMGLGWIVNYALMIRFSWKGRPHCMALLPLCNNIAWELTYTIVYPSANRVELLVFAIGLTLNFF
IMVGARRSARVEWRHSPLLSEHAGFILLVGTLLCFTGHVALAMEIGPGLAYSWGAVVCQLALSIGGLFQLLQRNSTAGTSWTLWSSRFLG
SCCTVAFAGLRCKYWPEVFGWLASPLVLWSLVTFLLADSAYGFCLYRVSHAETKARKKH
>SpdB
MDGFNNLQPPKEYQQIQWIADIFVTLMGVGWIVNYATMIWHAYTESTYSMALLPLCSNIGWELTFVLVYPSPNPYEFGVFASLLTLNLGI
MITAARAAPAEWAHSPLVANYAVPIFLIGILICFGGHVCLAAQIGPGLAYSWGAFICQLGLSVGAICQLLQRNSTRGTSWIMWLGRFLGS
CCTVVFAALRWKYWPEVFSWLGSPLMLWGVGVFFASELAYGLCFYLVQKSEVETKKSIKAQ
>AtS5B1
MDGFDHSTAPPEYNELKWLADIFVIGMAVGWVAHYVEMIHISFKDQTYCMTIGGLCINFAWEIIFCTMYPAKGFVERVAFLMGISLDLGV
IYAGIKNAPNEWHHTAMVRDHMPLVFAATTICCLSGHMALTAQVGPAQAYTWGAIACQLFISIGNVFQLLSRGNTRGASWTLWTSRFFGS
TSAIGFALVRYIRWWEAFSWLNCPLVLWSVVMFFLFEILYGALFYSVKRQEERSQCGIKHKER
>AtS2B
MDAFDLSTAPPEFASWATTLYACNIYTNFIWLYVYYGMIYRSYKDKSFAMPLISQCLNIAWEIVFGFLFSQDHWFITLSFQAAVISNCGV
IYAAIKYGAPEWNRSPMIQRNLPWIYIGGTLLAIAGHLALATELGMVRACFQGAIVCQAILSVGYVCQLLVRGSTRGFSLNLWFFRFTGS
LVMVPEFYIRVNYWPDAFSWLGEPFMLWCCFIYLGFDLAYPVLFWYIQRREKEEALAKSIKSL
>AfB
MDSFDLANAPPEIRAYATPIILLNLYTNASWLYVYFGMVYRSVKDKSYAMPLYSQCLNIAWEITYGYIYGDDWMLFATFLVTFPTDCLVI
WAAIYHGAKEWDRSPLVQRNLLWYYVIGTGIAVALHMCAASELGVEKAFFAGAIGCQAVLSVGYLGNLIQMGSTRGFSMHLWFFRFTGSL
TLVPEFYLRVKYWPERFGFLGQPLMLWCCAVFLGFDLVYGILFWYIRRQERETGMLLADGRKRK
>MacJ
MCFFALEEWAAANRDYENTPAPYWHVKSVPDGFTAISGILWSISYILMAKKAFKDRSYAMPLHCLCLNITWEAVYGFVYGPGLLNQVVFA
QWMIVDVVLFYAILRSAPYAWKQSPLVAQHLAGIIVVGCVICLWLHLAIAATFIPSIGRQVVFMTAWPMQVLINFSSIAQLLSRGNTLGH

SWGIWWTRMLGTIAAACCFFWRIHYWPERFGYAWTPYGKFLLLGSIGSDMVYAAVYVYVQRIEKQLDSLVNTKAQKAR
>AscF
MAFGVEPPEHVTPWFKPVYEATFQFGGVAWTLCYILIAREGMRTKSYGMPLFALANNFAWEMVYALWVVDNAFEKTAMTIWMLIDTPIIY
SILKHGVLEWQHAPMVSRNLKSILVGLIALCAAAHWSWQSWWIGNEMGKRDDLEGADLTQMAYWAVSMCQFLVSTMSLAMLCVRGHSGGV
SWMIWLSRFLGTLIGLNMNYAWAYYTWPEAHEYFMSAPAVFVWGVTTVCDIIYGFVLYHVKSNERELSDGRKVAAEADDEQVGGWSKMKT
GKN
>AlliB
MDPTDANGQVTFESREAPLSVAVLGTGIAVCWLANYVGMLCKSYQDRTYAMALMPLCCNFAWELVHGFITPEKNGMWPAVYVCWSGLNVA
VIYTAMKFAPNEWEHAPLVQRNIRYIFAISIVGWITVHLALVIHMGPHLAQGWGAMVCQLLLSAGALCQLLVRGSTRGTSFFLWATRFIG
TSLCLPHEILRYHYGYTDVIFNSPLGWWGTAAFFILDVSYGICLWNIYKYEETHQIKGGGRQKSI
>HomoB
MAQIDETYSSFGLSDDEPWVLTFLRVCTCVGWLANYMGMIYKSFRDRTYGMALMPLCCNIACEFVYGVIHAPDCPLYQIIFLCWFVLNCA
VIVAAIKFAPREWRHAPLVQQNIGWIFALSIVFWTTAHLAIVAQVGATEGAAWSSWFCQLFLSAGCLCQLIVRGSSRGTSLWIWFARFFG
TALSLPHEVLRYKHGYTNVVWHSPLGWWGGAAFFILDGAFGILLWKIQQSEKAVKGLQRGEVGLDGNDKDIKKSLSK
>FumiB
MGLIEGLQAALDYLENPTALTATSVSMCLCWLATYAGMVRKSFQDRSYSMPLMPLCCDIAFEFVFSVLYPPTEAPILQYVFGSWMAGSVM
VVFATIRFMPNEWKHAPLVQQNILSILAVTILGWMTAHLALVAQFGADDGSAWGGCLCQLLLGAGSLCQLIVRGSSRGASFFIWLSRFLG
TNLGVLYFTLRFRHGHVIWHSPLAWWSSVIAFLIDSCYGILLWHIRQAEGRDQQQRKAGSEIGPK
>XiaE
MVWLPPFLIPMSEVPPVTVGAADVSDLLFAAVAGPTALGWMVTYVLAIRQARRDGRTGIPAYLIAVNIAWEFSLTFLLEQTPTQRQINFL
WLVFNVFLFAQALRYGPRDYPGLSARTFRWTLAGVLVWASVVVMVGANELHDVDGMYTGMIIQVPLSAAFILMLRRRGSSAGQSMHIAVA
KTVGSLFAGLTAVIVYPSHHLLQVLVPTYVVLDVAYVVLLRRTMLREGRPLWAFRHPGAGVPGGCRLPVR
>DmtA1
MRPHSSKAPRGDMVDGIRMALAAVCGLGWTIAYVLAVRTGVRDKTYCIPLVALAMNICWEFQFVFFRSAEHMSGSNSEVEGAEVFIGIIW
LIVDCGLLYTVFRFGPNEFPYLPRRVFYAGFIVVLGLAYAGIEVLSREFDDGDVVLTSFGMNVAMSGLFLAMLAARQSSRGQSMGIAVAK
FVGTTSTCIAWFFDTSVYPGPWLPYCTVACVLLDVAYIAALSAVLRKERGRSQLAGDRPEIRDIERNASL
>TylF
MSISSTILNNICANSSGTFWLVTYIGIIWRGFKDRSLGMPLVALSANIAWEFLFAFIYIPERQLLHYATITWLFFDIPIVLQCFLYGAYD
THSQFMKKNFRLIFIAAIAICFVILFNLIPELKDTRGVYSGYGQNLMMSILFVDMILRRDDIRGQSLYIATTKLFGTLFAFLTSSFEFFE
LTAGVDVPFNINEFLTAIISSQTYPMTPLIKVIYPIIFVFDALYIVLVYQKSQEKGINPWVRF

Supplementary References

1. J. Tang and Y. Matsuda, *Chem. Sci.*, 2022, **13**, 10361-10369.

2. R. C. Edgar, *Nucleic Acids Res.*, 2004, **32**, 1792-1797.

3. J. Castresana, *Mol. Biol. Evol.*, 2000, **17**, 540-552.

4. M. N. Price, P. S. Dehal and A. P. Arkin, *PLOS ONE*, 2010, **5**, e9490.

5. A. Stamatakis, *Bioinformatics*, 2014, **30**, 1312-1313.

6. D. Darriba, G. L. Taboada, R. Doallo and D. Posada, *Bioinformatics*, 2011, **27**, 1164-1165.

7. T. Fujii, H. Yamaoka, K. Gomi, K. Kitamoto and C. Kumagai, *Biosci. Biotechnol. Biochem.*, 1995, **59**, 1869-1874.

8. F. Jin, J. Maruyama, P. Juvvadi, M. Arioka and K. Kitamoto, *Biosci. Biotechnol. Biochem.*, 2004, **68**, 656-662.

9. L. Chen, J.-W. Tang, Y. Y. Liu and Y. Matsuda, *Org. Lett.*, 2022, **24**, 4816-4819.

10. Y. Matsuda, T. Bai, C. B. W. Phippen, C. S. Nødvig, I. Kjærbølling, T. C. Vesth, M. R. Andersen, U. H. Mortensen, C. H. Gotfredsen, I. Abe and T. O. Larsen, *Nat. Commun.*, 2018, **9**, 2587.

11. X. Wei, T. Matsuyama, H. Sato, D. Yan, P. M. Chan, K. Miyamoto, M. Uchiyama and Y. Matsuda, *J. Am. Chem. Soc.*, 2021, **143**, 17708-17715.

12. D. R. McMullin, B. D. Green, N. C. Prince, J. B. Tanney and J. D. Miller, *J. Nat. Prod.*, 2017, **80**, 1475-1483.
